# Comparative Phylogeography of *Veronica spicata* and *V. longifolia* (Plantaginaceae) Across Europe: Integrating Hybridization and Polyploidy in Phylogeography

**DOI:** 10.3389/fpls.2020.588354

**Published:** 2021-02-01

**Authors:** Daniele Buono, Gulzar Khan, Klaus Bernhard von Hagen, Petr A. Kosachev, Eike Mayland-Quellhorst, Sergei L. Mosyakin, Dirk C. Albach

**Affiliations:** ^1^Institute for Biology and Environmental Sciences, Carl von Ossietzky University of Oldenburg, Oldenburg, Germany; ^2^Faculty of Biology, Altai State University, Barnaul, Russia; ^3^M.G. Kholodny Institute of Botany, National Academy of Sciences of Ukraine, Kyiv, Ukraine

**Keywords:** hybridization, polyploidy, comparative phylogeography, steppe, Europe, macroclimatic niche

## Abstract

Climatic fluctuations in the Pleistocene caused glacial expansion-contraction cycles in Eurasia and other parts of the world. Consequences of these cycles, such as population expansion and subsequent subdivision, have been studied in many taxa at intraspecific population level across much of the Northern Hemisphere. However, the consequences for the potential of hybridization and polyploidization are poorly understood. Here, we investigated the phylogeographic structure of two widespread, closely related species, *Veronica spicata* and *Veronica longifolia*, across their European distribution ranges. We assessed the extent and the geographic pattern of polyploidization in both species and hybridization between them. We used genome-scale SNP data to clarify phylogenetic relationships and detect possible hybridization/introgression events. In addition, crossing experiments were performed in different combination between *V. spicata* and *V. longifolia* individuals of two ploidy levels and of different geographic origins. Finally, we employed ecological niche modeling to infer macroclimatic differences between both species and both ploidy levels. We found a clear genetic structure reflecting the geographical distribution patterns in both species, with *V. spicata* showing higher genetic differentiation than *V*. *longifolia*. We retrieved significant signals of hybridization and introgression in natural populations from the genetic data and corroborated this with crossing experiments. However, there were no clear phylogeographic patterns and unequivocal macroclimatic niche differences between diploid and tetraploid lineages. This favors the hypothesis, that autopolyploidization has happened frequently and in different regions. The crossing experiments produced viable hybrids when the crosses were made between plants of the same ploidy levels but not in the interploidy crosses. The results suggest that hybridization occurs across the overlapping areas of natural distribution ranges of both species, with apparently directional introgression from *V. spicata* to *V. longifolia*. Nevertheless, the two species maintain their species-level separation due to their adaptation to different habitats and spatial isolation rather than reproductive isolation.

## Introduction

The past 30 years have seen an enormous increase in knowledge of how species migrated and evolved, thanks to the analyses of intraspecific genetic variation and progress in the field of phylogeography (Avise, 2000, 2009). Pleistocene refugia and migration corridors have been inferred (e.g., Hewitt, 2000; Harrison and Noss, 2017) and phylogeographic patterns among species have been compared to infer commonalities in dispersal history and differences, indicative of changes in community composition over time (e.g., Brunsfeld et al., 2001; Muellner-Riehl, 2019). Based on such information, hypotheses about the effect of climate change on biodiversity decline have been established (Keppel et al., 2012; Carvalho et al., 2019). A special situation occurs when species interact, i.e., they hybridize or form polyploids. In most cases, such studies examined the origin of hybrids or polyploids on a narrow spatial scale (e.g., Schönswetter et al., 2007; Balao et al., 2015). Undetected hybrids or structure caused by intraspecific lineages with different ploidy levels can potentially mislead analyses to infer genetically distinct groups or can inflate genetic diversity of particular groups of populations. Further, hybridization and polyploidy have potential large effects on a variety of processes shaping the distribution range of a species (Nieto Feliner, 2014; Arrigo et al., 2016; Paule et al., 2017), for example allowing species to exploit new ecological niches. Likewise, the advantages of polyploids over diploid progenitors are the increased number of alleles to mask the deleterious recessive mutations and lead to heterosis in allopolyploids (Gu et al., 2003), and neo-functionalization or sub-functionalization of duplicated genes copies. These factors are considered important for ecological niche expansion and provision of high flexibility toward environmental challenges (Adams and Wendel, 2005; Birchler et al., 2010), facilitating colonization of new and extreme habitats. In consequence, populations of a species affected by hybridization might have survived in places where the non-hybrid populations of that species normally would not be able to occur (Kadereit, 2015). These factors may be especially prevalent in phylogeographic analyses, in which both diploids and polyploids are widespread and sympatric. Here, we compared phylogeographic patterns of two species able to hybridize and assessed how hybridization and polyploidization might have affected these patterns considering past climatic changes. We have studied two species of the genus *Veronica* L., *Veronica spicata* L., and *Veronica longifolia* L. from *Veronica* subgenus *Pseudolysimachium* (W.D.J.Koch) Buchenau, both are widely sympatric across Eurasia and known to have two different ploidy levels, the polyploids (in our case, tetraploids) being derived by autopolyploidy (Bardy et al., 2011; Albach, unpubl.). The two species, however, differ in ecology. *Veronica spicata* occurs on nutrient-poor, open and sunny dry meadows, dunes, or rocks. It is considered to have been a member of the late-glacial steppe-tundra-community and occurs nowadays in the remnants of this vegetation (Pigott and Walters, 1954). It is highly intolerant of shade and competition (Pigott and Walters, 1954), which makes it endangered in large parts of its western distribution area. In contrast, *V. longifolia* is a species inhabiting nutrient-rich, mesic to moist habitats along rivers, lakes or even ditches or in swamps (Winter et al., 2008).

*Veronica* subgenus *Pseudolysimachium* includes ∼30 species distributed across Eurasia, with several of them used in horticulture, especially cultivars of *V. spicata* and *V. longifolia* (Albach et al., 2004; Kosachev et al., 2019). These are also the most common and widespread species of the group. The Altai region, with 13 reported species, is considered the center of its diversity; also, seven hybrids have been described there based on morphology (Kosachev et al., 2015 and 2016). The northern Balkan Peninsula is the second center of diversity of the group, with six species (Albach and Fischer, 2003). The origin of the subgenus has been estimated to have occurred 12.5 Mya (±4 My), likely caused by a polyploidization event (Meudt et al., 2015), thus what we consider diploids in this study are in fact ancient tetraploids. Nevertheless, more recent polyploidization events have occurred within the last 3 My (confidence interval 4–0.5 Mya) within the subgenus (Meudt et al., 2015). Besides polyploidization, introgressive hybridization events between more or less diverged lineages in the subgenus may have occurred. Hybridization has been reported repeatedly in the subgenus. For example, Kosachev et al. (2016), studied 18 species of *V*. subg. *Pseudolysimachium*, reported an unresolved phylogenetic structure based on cpDNA and ITS1, with most species being polyphyletic, and multiple samples of *V. spicata* and *V. longifolia* showing signs of introgression from the other species. Nevertheless, the ITS1 region at least resolved alleles into six groups with most alleles from *V. longifolia* in one group and those of *V. spicata* in another. Kosachev et al. (2016) also suggested an East Asian origin followed by repeated westwards range expansions across the Pleistocene Eurasian steppes with frequent interspecific hybridization events. Gene flow between *Veronica* species (*V. spicata, V. orchidea* Crantz, and *V. barrelieri* H.Schott) has been shown to be common in the Balkan Peninsula, with genetic isolation restricted to few geographic regions (Bardy et al., 2011). Potential hybridization between species in the subgenus has also been detected in the Altai Mountains using SRAP (Sequence-related amplified polymorphism) markers, albeit with weak support (Kosachev et al., 2019). Bardy et al. (2011) suggested that complete homogenization of the identified groups is prevented by ecological divergence, ploidy differences, and geographic isolation. While not being responsible for speciation, hybridization may have caused the present wide morphological variation by generating several variants, which are morphologically intermediate between the recognized species.

Hybridization in the subgenus, though not specifically between *V. spicata* and *V. longifolia*, has been reported in a number of places across Eurasia, such as in the Balkan Mountains, Eastern Alps, Southern Carpathians (Bardy et al., 2011), Altai Mountains (Kosachev et al., 2019; Khan et al., submitted), and possibly other regions (Kosachev et al., 2016). However, it is still unclear whether hybridization occurred more widely across the Eurasian steppes and between *V. spicata* and *V. longifolia* or not. The intermediate plants between both species inferred to be hybrids have taxonomically been called *V.* × *media* Schrad. Based on crossing experiments between several species of the subgenus, Härle (1932) and Graze (1933) reported that hybridization between plants with the same ploidy level is possible and the fertile hybrids are able to backcross with their parents. Both species, *V. spicata* and *V. longifolia*, have diploid (2*n* = 34) and tetraploid (2*n* = 68) populations (Albach et al., 2008). The two different ploidy levels found in *V. spicata* and *V. longifolia* (2× and 4×), however, do not seem to be related to differences in either geographical distribution, ecological requirements, or morphology (Trávníček et al., 2004). However, in earlier publications, Raitanen (1967) reported that morphologically determined samples of *V. spicata* and *V. longifolia* in Fennoscandia seem to hybridize despite differences in ploidy levels (with *V. spicata* occurring in the region only as 4× and *V. longifolia* mainly as 2×). According to Härle (1932), *V. spicata* and *V. longifolia* are self-incompatible, but Wilson et al. (2000) reported *V. spicata* as self-compatible. Similarly, Scalone et al. (2013) suggested that both species are facultatively outcrossing. Pollination in the subgenus is mostly performed by insects (entomophily) and is not species-specific (i.e., performed by non-specialized and mainly generalist pollinators), providing the possibility of hybridization in natural habitats. Bees (Hymenoptera: Apoidea, mainly small- to medium sized species) seem to be the most frequent visitors of the species, but Lepidoptera (Müller, 1881; Knuth, 1909; Ellis and Ellis-Adam, 1994; Albach and Kosachev, unpublished), flies (Diptera: small-sized Syrphidae and various other groups) and small-sized beetles (Coleoptera: Mordellidae, Nitidulidae, and some other non-specialized anthophilous groups) are also observed as occasional flower visitors (Kampny, 1995; Mosyakin, unpublished). It looks like the guilds of pollinators of *V. spicata* and *V. longifolia* are recruited from among the local faunas of non-specialist (both nectar-collecting and pollen-eating) anthophilous insects and thus may differ considerably in various regions. At the same time, such pollinator flexibility allows opportunities for cross-pollination within and between the two plant species, when they occur in close proximity. In addition, both species have rhizomes and can reproduce vegetatively by underground rhizome systems (Wilson et al., 2000), a trait considered important for the establishment and local-scale spread of hybrid and polyploid populations (Herben et al., 2017).

Here, we investigated the phylogeographic structure of *V*. *spicata* and *V*. *longifolia* species in parallel and estimated the extent of hybridization between both species. We employed genome wide SNPs (single nucleotide polymorphism) to analyze genetic diversity and hybridization/introgression in both *V. spicata* and *V. longifolia.* Furthermore, we conducted crossing experiments to test for the possibility of generating viable offspring from crosses between *V. spicata* and *V. longifolia* individuals of the same as well as different ploidy levels and geographic origins. Finally, we assessed the geographic distribution of ploidy levels in both species. We based our initial hypotheses on the works of Härle (1932) and Graze (1933), and the observation in the wild by Borsos (1967) and Trávníček et al. (2004). Our initial hypotheses were, first, that hybridization between *V. longifolia* and *V. spicata* is restricted to individuals having the same ploidy levels [e.g., *V. longifolia* (2×) × *V. spicata* (2×) and *V. longifolia* (4×) × *V. spicata* (4×)], while different ploidy levels [*V. longifolia* (2×) × *V. spicata* (4×) and *V. longifolia* (4×) × *V. spicata* (2×)] represent an important crossing barrier. Second, we also expected a significant genetic intraspecific structure across the native distribution ranges of the species reflecting ecological and spatial isolation in the wild. Lastly, we hypothesized that ecological niches of the two species did not differ based on the wide occurrence but considered it likely that macroclimatic differentiation between diploid and tetraploid individuals at both intra- and interspecific levels occurred in both species.

## Materials and Methods

### Sampling

**FIGURE 1:**
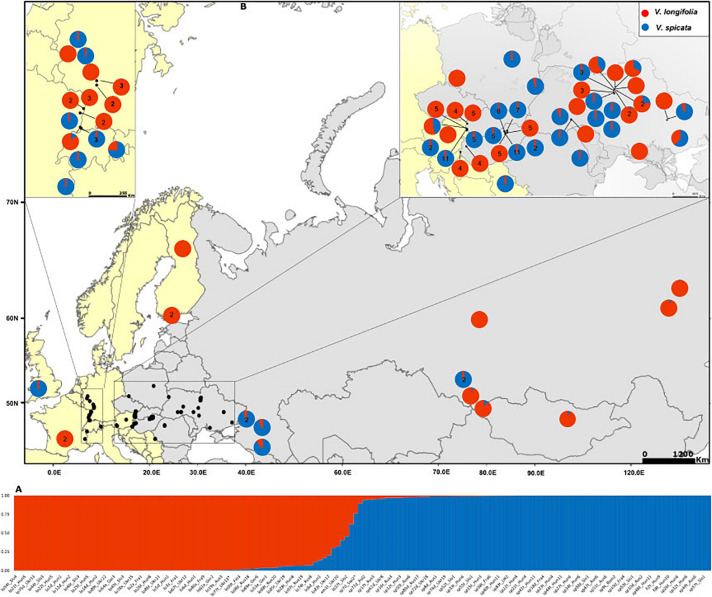
**(A)** Assignment of individuals to *K* = 2 clusters based on Bayesian statistics as implemented in STRUCTURE. Blue color represents the probability of *Veronica spicata* ancestry and red for *Veronica longifolia* ancestry. Complete details of all individuals with the same order are in Supplementary Table 1. **(B)** Distribution map of the sampled individuals with genetic composition based on *K* = 2. Pie charts without number represent only one individual whereas those with numbers give the number of individuals included from that particular locality. Genetic composition of the localities with more than one individual has been averaged accept where the difference among the individual’s genetic composition exceeded 10%.

We collected 177 accessions: 81 of *V. spicata*, 74 of *V. longifolia*, one putative hybrid (*V.* × *media*), and *Veronica schmidtiana* as an outgroup for the molecular analyses. *V. schmidtiana* has been used as outgroup since it is the only species unequivocally positioned within the subgenus and sister to the clade containing both ingroup species (Figure 1 and Supplementary Table 1). The accessions were collected on various field excursions during 2000–2019 in different areas across the distribution range of the species (Figure 1B) and identified using the morphological key provided in Albach and Fischer (2003). During the excursion, fresh leaf samples were collected and dried in silica gel (Voucher information; Supplementary Table 1). For crossing experiments, living plants of various accessions have been cultivated in the greenhouse of the Botanical Garden of Carl von Ossietzky-University (see details below). Vouchers for all crosses are deposited in the herbarium of Carl von Ossietzky University of Oldenburg (OLD).

**FIGURE 2:**
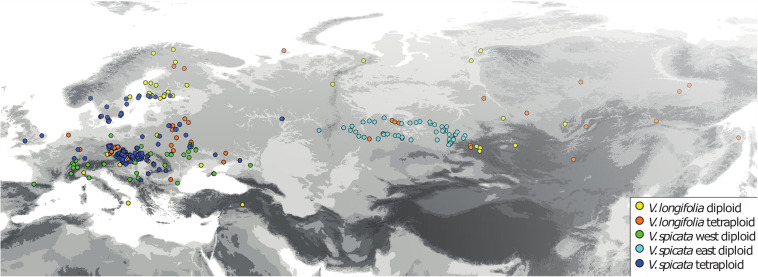
Geographical distribution of all *V. longifolia* and *V. spicata* individuals with known ploidy level (*N* = 404).

For the analysis of ecological niches, we generated a dataset of 404 specimens with known ploidy levels based on chromosome counting or flow cytometry and sufficiently precise geographical coordinates (about one arc minute resolution or better; Figure 2 and Supplementary Table 2) from literature data and our own estimations. New records of ploidy level directly measured by us constitute 38% of the data. In the first step, we excluded data points with less than 25 km linear distance from another point in each group (Table 1) using the geosphere package (Hijmans et al., 2019) for R (R Core Team, 2019). This reduces the chance that individuals might stem from the same (meta-) population and it is also convenient for statistical purposes when there is only one data point per grid square or less at the same time. This step also reduces a bias in geographical sampling, which can lead to distorted results (Fourcade et al., 2014). After thinning, we used a total of 248 data points. The threshold of using 25 km linear distance (and not an even greater distance) for exclusion was based on the assumption that a lower sample number is negatively affecting the statistical confidence. Therefore, we sticked to our 248 data set. Following our genetic analysis about 4% on average of all existing individuals in both species (or data points in the macroclimatic analysis) might have been compromised by introgression. It will become evident further below that the variation within each group and the overlap between groups was that high that these potential 4% could not have perturbed the results in any way. Plants for which potential hybrid influence was detected in our genetic analyses were not part of the macroclimatic analyses except for two individuals. One of these individuals was included in tetraploid *V. longifolia* and one in tetraploid *V. spicata* based on their typical morphology and the majority of the genetic markers found. Excluding both would not make any difference because their climatic parameters were typical for their respective groups.

**TABLE 1:** Number of points for which georeferenced data and ploidy level was known.

*Veronica* groups	All available	After thinning	Subsets
Diploid *V. longifolia*	48	40	
Tetraploid *V. longifolia*	102	65	
Diploid *V. spicata*	118	74	33 west, 41 east
Tetraploid *V. spicata*	136	69	
Total	404	248	

*In the second column only the points with more than 25 km linear distance from each other were included. Only diploid V. spicata was divided in two subsets for some analyses.*

### Flow Cytometry and DNA Extraction

Ploidy information of all samples was assessed by flow cytometry following Meudt et al. (2015) on a CyFlow SL (Partec, Germany). Overall, we report here 156 new measurements of ploidy for the two species. Since intra-population ploidy variation occurs rarely in *V*. subg. *Pseudolysimachium* (∼1.8% of the cases; Bardy et al., 2011), we measured for the majority of populations only one sample after confirming the finding of Bardy et al. (2011) for 12 populations in which we measured three individuals (Supplementary Table 2). DNA was extracted from about 20 mg of dried leaf material using innuPREP Plant DNA Kit (Analytic Jena AG, Jena, Germany) following the manufacturer’s instructions. The quality of DNA was checked using gel electrophoresis (1% agarose gel). Photometric DNA concentration and A260/A280 ratio was measured using TECAN infinite F200 Pro (Tecan Group AG, Männedorf, Switzerland).

### Genotyping by Sequencing

Several datasets from different years and sequencing runs were combined for the analysis in this study. Combinability of runs was checked in preliminary analyses including the same individual sequenced in different runs. All GBS (genotype by sequencing) libraries were prepared following Elshire et al. (2011), using the methylation-insensitive restriction enzyme *Msl*I (5′…CAYNN^NNRTG…3′) with modifications as described by Siadjeu et al. (2018). Briefly, 200 ng genomic DNA were included in restriction-digestion with one unit *Msl*I (New England Biolabs, NEB) in 30 μl (1× NEB4 buffer) at 37°C for 1 h and heat inactivated at 80°C for 20 min. Adaptor ligation was conducted with 15 μl of restriction product and 3 μl ligation adapters (Ovation Rapid DR Multiplex System, Nugen Technologies, Leek, Netherlands) in 12 μl of master mix (4.6 μl D1 water, 6 μlL1 ligation buffer mix, and 1.5 μlL3 ligation enzyme) at 25°C for 15 min and heat inactivated at 65°C for 10 min. The ligation product was finalized in 20 μl of the kits ‘final repair’ master mix at 72°C for 3 min which was then purified with magnetic beads (homemade Serapure beads). The purified ligated products were amplified with a low number of PCR-cycles (10 cycles) using 4 μl of 5× MyTaq buffer (Bioline), 0.2 μl polymerase and 1 μl (10 pmol/μl) of standard Illumina TrueSeq amplification primers. To remove small fragments from the libraries, all amplicons were purified again with magnetic beads. In this way, libraries were prepared for all individuals, normalized, and pooled. An additional step of purification was performed to remove the PCR polymerase through Qiagen MinElute Columns (Hilden, Germany) from the pooled library. The genomic library was sent to LGC genomics (Berlin, Germany) for sequencing.

### RAD-Seq *de novo* Assembly Into Loci and Genotyping of SNPs

Sequencing quality was checked using FASTQC version 0.11.9 (Andrews, 2010) and all the results in HTML format were aggregated in MULTIQC version 1.9 (Ewels et al., 2016). At the end, we included the reads with mean Phred quality score >28, without any adapter contamination and a per base *N* content close to 0. In addition, to ensure and avoid any type of false positive/negative errors in SNP calling, we employed process_radtags step from the STACKS version 2.4 (Catchen et al., 2013). The settings for process_radtags were: trimming after 135 bp; remove all uncalled bases with the option -c and low quality scores with the option -q. After confirming upon the quality of the sequencing, we used STACKS version 2.4 (Rochette et al., 2019) pipeline to assemble the raw reads into *de novo* loci and to genotype the SNPs. The pipeline consists of the sequential execution of different tools as (I) USTACKS, (II) CSTACKS, (III) SSTACKS, (IV) TSV2BAM, (V) GSTACKS, and (VI) POPULATIONS. USTACKS was used to construct a set of putative loci and detect SNP at each locus following: maximum distance allowed between stacks = 4; minimum depth of coverage to create a stack = 5; maximum number of stacks at a single *de novo* locus = 5. Some of these settings were dictated by having tetraploid individuals in the dataset. Subsequently, the tool CSTACKS was executed to build a catalog (a set of consensus loci) from the set of processed samples, allowing 4 mis-matches between sampled loci when building the catalog. SSTACKS was then used to search a set of stacks against the catalog produced. We used TSV2BAM to transpose the R2 reads, by orienting it to their corresponding loci instead by sample associated with each single-end locus assembled *de novo* in the previous steps. Following TSV2BAM, we assembled the paired-end reads into a contig, merging the contig with the single-end locus, and aligned reads from individual samples to the locus within GSTACKS. Lastly, the tool POPULATIONS from STACKS was used to filter the data and produce VCF (Variant Call Format, ver. 4.2) files. The minimum percentage of individuals across populations required to process a locus (option −R) was selected as 0.5 after inspection of several phylogenetic trees obtained with various values of −R option (0.9, 0.75, 0.5; results not shown) since this setting has important effect on the maximum missing data (for example with *R* = 0.5, 50% of missing data). At the end, we generated two different VCF files: one with more than one SNP per locus (concatenated), and one with a single (the first) SNP per locus (using the populations option –write-single-snp), assuming that loci are independent (e.g., unlinked). In the downstream population genomics, we only used the biallelic SNPs assumed to be unlinked to ensure that allele frequencies are not correlated. We included the post process filtering step to remove the variants with low (<8) and high coverages (>800). This step is necessary as the variants sequenced at very low coverage may represent only one of four alleles in tetraploids and the one with high coverage may result from repetitive regions. Subsequently, we removed the variants and individuals with more than 50% missing data. This post-process step was performed using the R package *VCFR* (Knaus and Grünwald, 2017) and self-written R script (Supplementary Material 1). The tool *PARALOG-FINDER* implementing the method of McKinney et al. (2017) was applied to remove paralogs in our dataset before the downstream analyses, however, no appreciable differences in the results were found and we kept the original datasets. Our final dataset for population genomics included 27% missing data for concatenated loci (290114 SNPs; 153 individuals) and 25.73% missing data on unlinked loci (8723 SNPs; 157 individuals).

The two datasets contain different numbers of individuals as the post process filtering step removed samples using the same limit of missing information admitted, which differs slightly between samples from unlinked and concatenated dataset (since on unlinked dataset missing data is related with only the first SNP per locus; Supplementary Table 1).

### Phylogeographic Structure and Admixture

To assess the genetic structure involving all individuals of both species sampled across the sampling area, we firstly adopted a non-hierarchical approach in STRUCTURE version 2.3 (Pritchard et al., 2000) using the unlinked dataset. STRUCTURE groups the individuals originating from different genetic clusters and identifies migrants. It is also useful in studies involving hybridization using Bayesian statistics by applying Markov Chain Monte Carlo (MCMC) and multiple repetition (Pritchard et al., 2000; Porras-Hurtado et al., 2013). We included all individuals of *V. longifolia* and *V. spicata* in STRUCTURE analysis to assign them to their respective genetic clusters, find the role of geography in their structuring and investigate admixed individuals resulted from the contribution of alleles from both species. The analysis was run for 100000 MCMC (Markov chain Monte Carlo) burning the initial 10% chains, with the admixture model and correlated allele frequencies between populations, from K one to four each with 20 iterations, considering that migration is still occurring. This analysis included morphologically defined *V. spicata* (81 individuals), *V. longifolia* (74 individuals) and *V.* × *media* (one individual). To investigate the role of geography in sub-structuring of both species, we ran an additional second-level hierarchical analysis as well including only samples of *V. spicata* and *V. longifolia* with the same parameters except *K* = 13. We used the program CLUMPP version 1.1.2 (Jakobsson and Rosenberg, 2007) to clump and align all the 20 iteration for the optimal *K* value following the Evanno approach as employed in STRUCTURE HARVESTER version 0.6.94 (Evanno et al., 2005; Earl and von Holdt, 2012) selecting the Full-Search option. To further explore the role of geography, we used TESS3R package in R (Caye et al., 2016; Supplementary Material 2) as well. TESS3R determines genetic variation in natural populations considering both genetic and geographic data simultaneously. We used the output results of the second level hierarchical analyses on *V. spicata* and *V. longifolia* from STRUCTURE (results not shown) and combined them with geographic coordinates of all the included individuals.

To check the relationship among the individuals from different geographical localities and complement the STRUCTURE/TESS3R results, we further constructed the maximum likelihood trees as well using the concatenated SNPs data. For this analysis, we used two different datasets, one including all individuals and another one excluding admixed individuals (based on STRUCTURE results). We considered the individuals admixed which had admixture proportion more than 20%. The best substitution model of evolution was assessed with JMODELTEST-NG (Darriba et al., 2019), which provided GTR + I as best model with both BIC and AIC criteria. The actual analysis was then carried out in RAxML version 8.2.12 (Stamatakis, 2014). To inform RAxML about the nature of the dataset containing only concatenated SNPs without invariable sites, we applied the ASC correction as suggested (Stamatakis, 2014) to minimize the impact on branch lengths. To check the robustness of our results, we ran the analysis with 100 bootstrap replicates. In addition to phylogenetic reconstruction, pairwise *F*_*ST*_ statistics were also calculated (Wright, 1949) following Weir and Cockerham (1984). This analysis was implemented using the function stamppFst of the R package STAMPP (Pembleton et al., 2013; Supplementary Material 3) on the concatenated dataset with 100 bootstrap replicates and a confidence interval of α = 0.05.

### Characterization of Hybrids Classes and Introgression

After getting the admixing proportion through Bayesian approach between *V. longifolia* and *V. spicata* in STRUCTURE, we allocated the samples to distinct hybrid or pure classes, specifically, we tested to identify the presence of parent species individuals, F1 or F2 hybrids and backcrossed individuals to both species [parental *V. longifolia* (A) or *V. spicata* (B)]. We only included the individuals for which no single class had *Q* ≥ 0.80 probabilities to belong to one parent species. We implemented this analysis using the program NEWHYBRID (Anderson and Thompson, 2002). NEWHYBRIDS assign individuals to specific hybrid classes by looking at the patterns of gene inheritance within each locus. Since NEWHYBRIDS cannot handle large datasets, we created subsets of 200 unlinked most informative and differentiated SNP loci (method = *AvgPIC*) using the function *gl.nhybrids* included in the R package *DARTR* (Gruber and Georges, 2019; Supplementary Material 3). This function identifies loci that exhibit a fixed difference by comparing two sets of parental populations and creates an input file for the program NewHybrids. To avoid any further bias caused by the heterozygous loci of parental populations, we only included the pure individuals, those showing less than 1% of admixture from STRUCTURE analysis. Additionally, to get the robust results, we run the program with no prior information regarding the status or class of individuals. The analysis was run for 10^5^ MCMC iterations with a burn-in threshold of 10^4^. To run the program NEWHYBRID, we used the R package PARALLELNEWHYBRID (Wringe et al., 2016, 2017).

To check the possibility of introgression between both species, we measured genomic clines in the admixed individuals shared by the parental species as implemented in INTROGRESS (Gompert and Buerkle, 2009, 2010, 2012). INTROGRESS analyzes introgression of genotypes between divergent lineages and hybridizing species. In addition, INTROGRESS estimates genomic clines from co−dominant, dominant, and haploid marker data, without any requirement of fixed allelic differences between parental populations for the sampled genetic markers. The package tests for deviations of loci from neutral expectations as well. INTROGRESS is ideal in cases with high number of informative markers in which the potentially admixed individuals cover the full range of variation between parental species (Gompert and Buerkle, 2010). Since our data is based on continuous sampling, without any spatial correspondence, INTROGRESS suits well to our model. Briefly, we divided the data into three groups, two homozygotes (pure parental) and one heterozygote genotypes (admixed individuals), based on the NEWHYBRID and STRUCTURE results. These groups included 63 individuals of *V. longifolia*, 79 individuals of *V. spicata* and 13 admixed individuals (*Q* ≥ 0.2). In the actual analysis, firstly, we estimated hybrid indices and then genomic clines using multinomial regression. We measured introgression for each individual locus in admixed individuals relative to clines using their genotype frequency along an admixture gradient (Buerkle, 2005; Gompert and Buerkle, 2010) by comparing likelihood of regression models to that of a neutral model. The significance of likelihood was accessed using 1000 permutations. Lastly, we counted the introgressed loci from the observed hybrid genotypes by comparing with the probability densities of homozygotes and heterozygotes genotypes as suggested (Nolte et al., 2009). We adjusted the neutral expectation of genomic clines through multiple comparisons using the false discovery rate (FDR) as suggested (Benjamin and Hochberg, 1995; examples in Harrison et al., 2017; Khan et al., 2020).

### Crossing and Germination Experiments

We conducted crossing experiments between individuals of *V. spicata* and *V. longifolia* of both ploidy levels and geographic origins to assess experimentally reproductive barriers between the two species. The experiments were conducted in the greenhouses of the Carl von Ossietzky-University (Oldenburg, Germany) in spring and summer 2019. When enough inflorescences were available on a single plant, three treatments were applied for each replicate beside the controls (Ctr): H (hybridization), C (negative control) and CS (cage + selfing). To avoid any unwanted pollination from insects, all floral spikes were bagged using tea-bag filters. In treatment H self-pollination was tried to avoid by emasculation of the flowers on daily basis before anthesis. When the flowers opened, the freshly dehiscent anthers of the donor species were removed with clean tweezers and their pollens were transferred by direct contact with stigma on the receptor species. Negative controls (treatment C) were used to determine the minimum amount of pollen contamination acceptable after applying only emasculation. Negative controls were performed in parallel with H on the same plant. Spikes for treatment CS were bagged before anthesis till fruit maturation. Further, the controls (Ctr) were used to determine the germination rate of seeds pollinated with pollen of the same species. They were handled in a similar way to H with the exception that the pollen donor was from the same species and ploidy level as the control.

Seeds obtained from the crossing experiment were sterilized following the protocol suggested by Pickens et al. (2003): (1) 70% ethanol for 2 min; (2) rinse in distilled water for 5 min; (3) 2.6% NaOCl + Tween 20 for 40 min; (4) rinse in sterile water twice for 5 min each. After sterilization, seeds were placed in Petri dishes on filter paper, adding sterilized distilled water. A climate chamber was used to ensure controlled temperature and light conditions, with the settings: light 12/12 h; and temperature 21°C. The position of the single Petri dishes was randomized inside the chamber in order to account for small variation of temperature and light related to the position. Germination rate was defined as the number of seeds germinated after 30 days divided by the total number of seeds plated.

### Polyploidy and Ecological Differentiation

All niche analyses are based on the WorldClim data set (Hijmans et al., 2005) with a resolution of 2.5 min. The 19 bioclim variables were attached to the occurrence data of 248 individuals (see sampling) using DIVA-GIS (Hijmans et al., 2012).

In a preliminary principal component analysis (PCA) including all variables, we found that many of the variables were highly correlated with each other. To avoid collinearity and type II errors caused by high correlation, we applied pairwise Pearson product correlations in R (R Core Team, 2019). This Pearson correlations test retrieved bioclim variables with mod ≥ 0.8 such as: bio4 and bio7; bio5 and bio10; bio1, bio6, bio9, and bio11; bio12, bio13, bio14, bio16, bio17, bio18, and bio19, and the remaining four variables, i.e., bio2, bio3, bio8, and bio15 were not correlated. In the actual analysis we used eight variables, excluding the remaining 11 variables. The choice of the four variables that showed correlation with other variables was based on criteria such as longer time period over shorter period in the definition of the variables and more or less subjective hypotheses about the biological importance in our species. The eight variables included were: bio2 mean diurnal range, bio3 isothermality, bio7 temperature annual range, bio8 mean temperature of wettest quarter, bio9 mean temperature of driest quarter, bio10 mean temperature of warmest quarter, bio15 precipitation seasonality, and bio17 precipitation of driest quarter.

The reduced data set with 248 specimens and eight bioclimatic variables included was subjected to a PCA using NTSYSPC version 2.2 (Rohlf, 2009). It turned out that the Siberian samples of diploid *V. spicata* (>50° East) grow under rather distinctive climatic conditions. Tetraploid *V. spicata* do not occur there. To accommodate for this, we repeated several of our analyses separating the Siberian samples (>50° East) from diploid *V. spicata* from the West (Table 1). Such a problem did not arise in *V. longifolia*, because its diploid and tetraploid groups occur in the same general geographical regions or there were only very few data points in the one group when far distant from the other group.

To qualitatively compare climatic niche models (or in other words: the macroclimatic envelopes of a group projected on a geographical map) between conspecific diploid and tetraploid groups, we used MAXENT version 3.4.1 (Phillips et al., 2006, 2019) on the data set described above (248 data points divided in four or in five groups). For the application of MAXENT, we followed Merow et al. (2013) and Fourcade et al. (2014), who recommended minimizing the correlation among predictors. Therefore, we again used only the eight bioclim variables as described above. For purposes as in our study, they recommended to use the relevant and complete geographical space for background selection. We therefore first limited the bioclim layers in DIVA-GIS (Hijmans et al., 2012) to 5°W, 135°E, 35°N, and 75°N (Figure 2) and used this restricted rectangle of the eight climatic layers in MAXENT. It was not possible to account for sampling effort in our data, because the data was opportunistically gathered from different times and sources. However, a facultative exclusion of the densely sampled Siberian *V. spicata* and the thinning of all data to 25 km linear distance follow the suggestions of Fourcade et al. (2014) to minimize geographically biased sampling. We tested different output options in MAXENT and chose the logistic output because the results seemed less diffuse (steeper and therefore clearer gradients) than with the other options.

We investigated differentiation in macroclimatic niches between diploids and tetraploids among individual variables using the analysis of variance (ANOVA) tool in EXCEL 2016 (Microsoft Corp., Redmond; significance considered *p* < 0.05). All variables were tested for both taxon combinations and this might lead to statistical type I errors. Therefore, we additionally applied Bonferroni corrections for all 19 parallel comparisons, which leads to *p* < 0.003 as a much more conservative threshold for significance. Since the differences found between European and Siberian samples of diploid *V. spicata* could be misleading (see below), we repeated the ANOVA for *V. spicata* including only the western samples (west of 50° East).

Finally, to get a quantitative idea about climatic niche differences between the ploidy levels or between species, we calculated Schoener’s *D*, a frequently used index for niche overlap, which ranges between 0 and 1 (the latter stands for complete niche overlap) from the original MAXENT results. We used the precompiled version 1.3 for windows of ENMtools (Warren et al., 2008, 2010) for this and also for the following analysis. To test whether the MAXENT scores exhibit statistically significant ecological differences between several groups, we performed a niche identity test using Schoener’s *D* as a test variable. In this test the empirical occurrence points were pooled, their identity randomized, subjected to a MAXENT analysis, and Schoener’s *D* was calculated. We did 100 pseudo-replicates per comparison. The resulting distribution of Schoener’s *D* represents the null hypotheses that niches differ by chance only and it can be compared with the *D* score of the original data to judge about the probability that niches are identical.

## Results

### Phylogeographic Structure and Admixture

**FIGURE 3:**
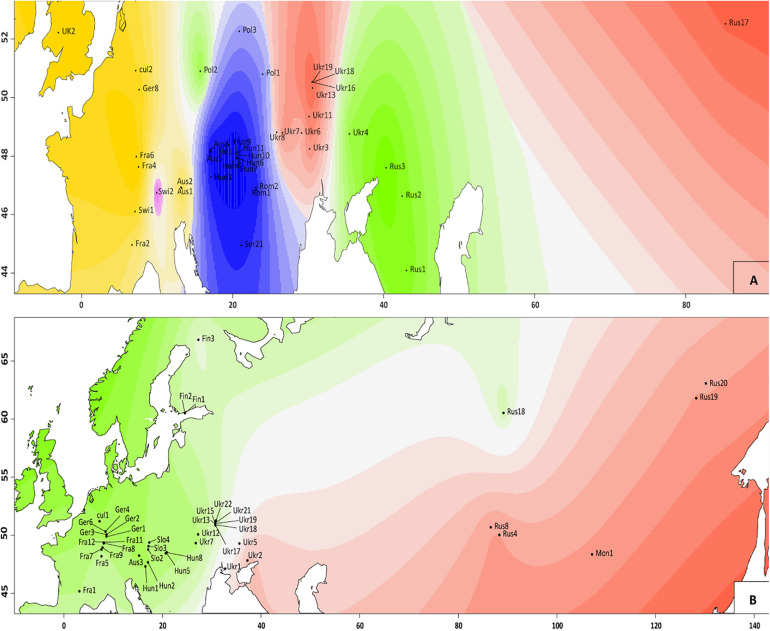
Genetic clusters identified with TESS3R analysis in *V. spicata* **(A)** and *V. longifolia* **(B)** populations. Different colors indicate different clusters inferred (two for *V. longifolia* and five for *V. spicata*) and different shading indicates different probabilities to belong to the respective cluster. The graphs are based on the results of STRUCTURE between individuals in the populations, as employed in R package tess3r (details are in main text).

The Bayesian modeling to assess the genetic structure clustered all individuals in two groups (Evanno method; *K* = 2; Figure 1A) corresponding to *V. spicata* and *V. longifolia*. This analysis clearly showed that the genetic structure of both species is in accordance with their morphology-based classification. However, our results also recovered individuals with admixture higher than 20% (Figure 1A and Supplementary Table 1). The threshold of 20% was subsequently used to infer hybrids since individuals with more than 20% did not group according to geography in other analyses. The second level hierarchical clustering on species alone found optimal intraspecific clustering in *V. spicata* at *K* = 2 (result not shown). However, after averaging the results of STRUCTURE between individuals in the populations and using the R package TESS3R to plot those averages on a map, we recovered a reasonable geographical pattern until *K* = 5 (Figure 3). The Evanno method indicated an optimal *K* = 2 for the second level hierarchical clustering analysis using the dataset of *V. longifolia* in STRUCTURE and TESS3R was used to plot the results. At *K* = 2, the individuals were structured into one group composed of individuals from Asia and the other including those from Europe (Figure 3) except for one individual from Siberia [Rus18 (lo96t_Rus18)] grouping with European individuals. Admixture between the two species based on the STRUCTURE analysis, considering individuals with *Q* ≥ 0.20 for the minor ancestry as admixed, occurred in seven individuals. Of these, four individuals were *a priori* identified as *V. longifolia*, two as *V. spicata*, and one considered a hybrid. Admixture occurred in both ploidy levels, six in tetraploids and one in a diploid. It was found widely from Switzerland to Ukraine. The divergence between *V. spicata* and *V. longifolia* was lower, with an *F*_*ST*_ value of 0.296 ± 0.002, than between *V. longifolia* and *V. schmidtiana* (*F*_*ST*_ = 0.625), and between *V. spicata* and *V. schmidtiana* (*F*_*ST*_ = 0.632; Supplementary Table 3), while highly significant for all (*P* = ∼0.0).

**FIGURE 4:**
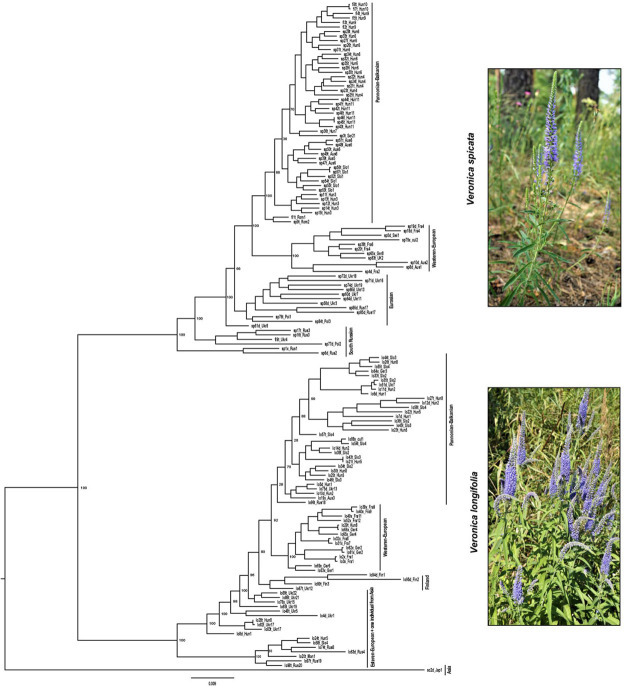
Phylogenetic analysis based on maximum likelihood statistics as implemented in RAxML. Two main groups with high support correspond to *V*. *spicata* (upper part) and *V*. *longifolia* (lower part). The IDs assigned to the samples (e.g., lo82t_Ukr17) are constructed using the first two letter to identify the species, e.g., lo for *V*. *longifolia*, sp for *V*. *spicata*, and sc for *V*. *schmidtiana*, followed by a unique number for every individual and a letter to indicate the ploidy level (*d* for 2×, *t* for 4×, and × for unknown ploidy). Underscore separates the information related with population constituted by 3 letters to identify the country (e.g., CzR for Czechia) and a unique number in that species (see complete detail in Supplementary Table 1).

Similarly, the phylogenetic analysis based on maximum likelihood also clustered all individuals into two main groups with high support corresponding to *V. spicata* and *V. longifolia*. Since individuals admixed according to STRUCTURE appeared basally branching and not in geographical meaningful clusters, we consider in the following only the analysis without admixed individuals (Figure 4 and see Supplementary Figure 1 for results including hybrids). The phylogenetic analysis also recovered sub-clustering representing Asian/East European individuals and more western European individuals in both species, except lo96t_Rus18. This individual was cultivated in a Russian botanical garden with an unknown geographic origin and placed in a group that contains individuals from Central and Western Europe (France, Germany, Switzerland, and Austria), which suggests that the garden did not cultivate a local genotype.

### Putative Hybrid Individuals and Introgression

**FIGURE 5:**
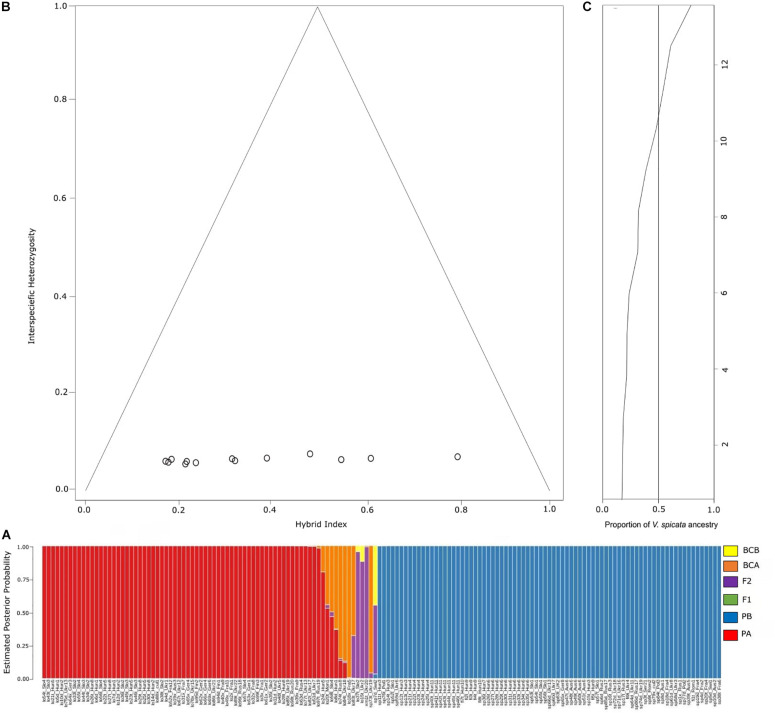
**(A)** Probability assignment into different hybrid categories according to NEWHYBRIDS analysis. PA, parental population A (*V. longifolia*); PB, parental population B (*V. spicata*); BCA, backcross with parental population A; BCB, backcross with parental population B; F1, F1 hybrid between populations A and P; F2, F2 hybrid between two F1 individuals. **(B)** Triangle plot detailing the relationship between interspecific heterozygosity against hybrid index of *V. longifolia*, *V. spicata* and admixed individuals. The hybrid index was calculated as proportion of *V*. *longifolia* and *V. spicata* alleles (*h* = 0 indicates individuals with alleles from *V*. *longifolia* ancestry, and *h* = 1 denotes *V. spicata* ancestry). We considered those individuals from the STRUCTURE presenting *Q* ≤ 0.80 as putative hybrid individuals; and **(C)** ancestry plot showing probability based on hybrid indices of *V. longifolia* (Hybrid index = 0) and *V. spicata* (Hybrid index = 1) ancestry across all 13 admixed individuals.

Probabilities to be parental species A or B (*PA* for *V. longifolia* and *PB* for *V. spicata*) obtained in NEWHYBRIDS followed the same pattern as explored with Bayesian statistics in STRUCTURE (Figure 5A). The NEWHYBRID results did not recover any individual to be an F_1_ hybrid. The estimated posterior probability to be an F_2_ hybrid was high (>0.88) for lo37t_Slo2, lo15t_Ukr2, and xm1t_Ukr20, while for sp7d_Swi2 and lo83t_Ukr17 the probability to be F_2_ was lower (0.51 and 0.32, respectively). Sample sp7d_Swi2 had a posterior probability of 0.45 to be a back-cross with *V. longifolia* parental group. Similarly, samples sp73t_Ukr19, lo18x_Fra5, lo83t_Ukr17, lo84t_Ukr18, and lo74t_Rus8 showed high probabilities (>0.6) to be backcrossed individuals of *V. longifolia*, whereas lo8d_Hun1, lo58t_Slo4, lo20t_Mon1, and lo24t_Hun5 had lower probabilities (<0.6) to be in the same category (Figure 5A). Similarly, the hybrid indices <0.78 or >0.18 recovered with INTROGRESS with very low heterozygosity ∼0.02, suggest that the hybrid individuals are fertile (Figure 5B).

The genomic clines approach implemented in INTROGRESS supported the hypothesis of higher probability of homozygotes in admixed individuals than expected under neutral expectations. The homozygosity inferred was 41% from *V. longifolia* and 51% from *V. spicata* (Figure 5C). We recovered only one heterozygous locus in all admixed individuals, reflecting high homozygosity. The results infer a higher tendency of homozygotes clines from *V. spicata* with 216 more SNPs than *V. longifolia*. This tendency of 18% more homozygotes from *V. spicata* suggests asymmetrical introgression. For this analysis we only used the non-neutral loci, showing deviation from a model of neutral introgression based on genome-wide admixture (*P* ≤ 0.025; significance following FDR correction; Table 2).

**TABLE 2:** Patterns of introgression based on all admixed individuals.

Patterns of Introgression relative to neutral expectations	No of Loci
**Non-neutral**	1793
*V. longifolia* (Parent A) homozygotes to admixed individuals	472
*V. spicata* (Parent B) homozygotes to admixed individuals	688
Heterozygous excess in admixed individuals	1
Heterozygous deficiency in admixed individuals	1603
Homozygous excess in admixed individuals	1160
Homozygous deficiency in admixed individuals	2
**Neutral**	6930

*The results are based on pure parental individuals of V. longifolia (63 individuals with 0.1 ≥ Q ≥ 0.9), V. spicata (79 individuals with 0.1 ≥ Q ≥ 0.9), and admixed individuals (13 with Q ≥ 0.2) following the NEWHYBRID, and STRUCTURE results. The total number of SNPs used were 8,723 with 25.73% missing data. Prediction of introgression at each locus was compared through likelihood of regression model to that of a neutral model and its significance by using 1,000 permutations. Non-neutral loci were selected by adjusting the neutral expectation of genomic clines through multiple comparisons (P < 0.025; significance following FDR correction).*

### Crossing and Germination Experiments

We have used 32 individuals during the crossing experiment (11 of *V. spicata* and 21 from *V. longifolia*). Overall, 24 crossings were performed in which 13 were based on the same ploidy and 11 on different ploidy levels (Table 3). In almost all cases, the negative controls (*n* = 20) resulted in the production of zero seeds except in two cases, in which the number of seeds was 18 and 4. In successful crosses, we got usually more than 100 seeds per spike. We considered the crossing as successful only when it led to production of more than 20 seeds per spike, attributing the eventual production of small amounts of seeds to contamination by small arthropods, which were able to reach the spikes through the lower opening of the bag. Crossings performed between plants of the same ploidy level (2× × 2× and 4× × 4×; Table 3), even from very distant geographical origin, resulted in production of viable seeds in all cases, with germination rates ranging from 18 to 100%. However, the crossings between different ploidy levels (2× × 4× and 4× × 2×; Table 3), produced almost no seed in crosses with the tetraploid mother, and in four out of seven crosses when the mother was diploid (Table 3). Plants of crosses, which produced less than 10 seeds, did not grow to fully developed plants, except for a single plant. This plant was determined to be tetraploid (Albach S372 × Albach S634). All five plants of the cross Albach 1450 and Albach S854 analyzed flow cytometrically were tetraploids. Six of the plants from the cross between Albach 1450 and Albach S485 were analyzed with flow cytometry and all were triploid. The controls between plants of the same species and ploidy level showed a high production of seeds and germination rates (>93%) except for the crossing of diploid *V. spicata*, which could be due to sterility or self-incompatibility reactions.

**TABLE 3:** Crossing experiment results.

Hybrids (H)										

Ploidy	♂		♀						ID	
	
(♂ × ♀)	Species	Origin	Species	Origin	Seeds obt.	Seeds plated	Germ.30 d	Germ.%	♂	♀
	*V. spicata*	Switzerland	*V. longifolia*	Altai Mount.	>100	50	47	94	Albach S526	Albach S625
	*V. longifolia*	Altai Mount.	*V. spicata*	France	>100	50	50	100	Albach S625	Albach S758
	*V. longifolia*	Altai Mount.	*V. spicata*	Switzerland	>100	50	48	96	Albach S625	Albach S526
**2× × 2×**	*V. longifolia*	Finland	*V. spicata*	Romania	>300	50	50	100	Albach S848	Albach S376
	*V. longifolia*	Finland	*V. spicata*	Hungary	>100	50	46	92	Albach S849	Albach S748
	*V. longifolia*	Finland	*V. spicata*	Poland	>100	50	9	18	Albach S848	Albach S485
	*V. longifolia*	Finland	*V. spicata*	Poland	>100	50	13	26	Albach S849	Albach S485
	*V. spicata*	France	*V. longifolia*	Finland	>50	50	12	24	Albach S758	Albach S849
	
	*V. longifolia*	Finland	*V. spicata*	Hungary	0	0	0	0	Albach S848	Albach S869
**2× × 4×**	*V. spicata*	Altai Mount.	*V. longifolia*	Ukraine	∼10	0	0	0	Albach S634	Boiko 020
	*V. spicata*	Poland	*V. longifolia*	Czechia	0	0	0	0	Albach S485	Albach S499
	
	*V. longifolia*	Yakutia	*V. spicata*	France	4	4	4	100	Albach S372	Albach S758
	*V. longifolia*	Yakutia	*V. spicata*	Altai Mount.	9	9	6	67	Albach S372	Albach S634
**4× × 2×**	*V. longifolia*	Ukraine	*V. spicata*	Poland	>50	28	19	68	Albach 1450	Albach S485
	*V. longifolia*	Ukraine	*V. spicata*	Poland	∼100	50	1	2	Albach 1450	Albach S485
	*V. longifolia*	Ukraine	*V. spicata*	Altai Mount.	0	0	0	0	Boiko 020	Albach S634
	
	*V. longifolia*	Ukraine	*V. spicata*	Hungary	>100	50	49	98	Boiko 020	Albach S869
**4× × 4×**	*V. spicata*	Hungary	*V. longifolia*	Czechia	>100	50	48	96	Albach S869	Albach S499
	*V. spicata*	Hungary	*V. longifolia*	Ukraine	>100	50	50	100	Albach S869	Albach 1450
	*V. longifolia*	Ukraine	*V. spicata*	Hungary	>100	50	40	80	Boiko 020	Albach S869

**Positive Control (Ctr)**										

**2× × 2×**	*V. longifolia*	Finland	*V. longifolia*	Finland	∼30	33	31	94	Albach S849	Albach S849
	
	*V. spicata*	Austria	*V. spicata*	Austria	>50	50	50	100	Albach S749	Albach S749
**4× × 4×**	*V. spicata*	Austria	*V. spicata*	Austria	>100	50	50	100	Albach S749	Albach S749
	*V. longifolia*	Czechia	*V. longifolia*	Czechia	∼37	35	34	97	Albach S499	Albach S499
	*V. longifolia*	Czechia	*V. longifolia*	Czechia	>50	50	50	100	Albach S499	Albach S499

*The number of seeds obtained refers to single floral spikes. Germ. 30d refers to seed germinated 30 days after plating. ID refers to vouchers in herbarium OLD. Hybrids indicate crossings between different species, while positive controls crossings between same species and ploidy level. The IDs assigned to the samples (e.g., sp30aSwi_d1) were constructed using the first two letter to identify the species (sp for V. spicata, and lo for V. longifolia), followed by a unique letter e.g., 30a representing an individual of population 30, and 3 letter code of country of origin (e.g., Swi for Switzerland). An underscore separates the information related with polyploidy e.g., t for tetraploid and d for diploid.*

### Polyploidy and Ecological Differentiation

Based on our literature search and our own results, we demonstrate that diploids and tetraploids are about equally common in *V. spicata* (115 vs. 138 measurements, respectively), whereas diploids are less common in *V. longifolia* (49 vs. 102 measurements, respectively). However, these numbers are biased by our sampling and the accessibility of populations.

The ANOVA results for the diploid/tetraploid comparison of eight climatic variables are given in Table 4 (results for all 19 variables are given in Supplementary Table 4). When Siberian diploid samples of *V. spicata* were excluded, mainly the variables dealing somehow with precipitation (e.g., bio17 in Table 4) were significantly different between diploids and tetraploids. However, when all *V. spicata* diploids were included, variables somehow dealing with temperature or seasonality (e.g., bio2, 3, 7 in Table 4) were significant. This discrepancy is a clear indication and consequence of the distinctly continental climate in Siberia in contrast to the sub-oceanic climate in western and central Europe. The difference between diploids and tetraploids within *V. longifolia* seemed to be less pronounced and was indicated by five significant variables (three after Bonferroni correction). These variables (e.g., bio8 and 10 in Table 4) basically refer to different summer temperatures.

**TABLE 4:** One-way ANOVA calculations comparing bioclim variables (Hijmans et al., 2005) of diploid (2) and tetraploid (4) *Veronica longifolia* and of diploid (2) and tetraploid (4) *Veronica spicata*.

Bioclim variable	*V. longifolia* 2/4			*V. spicata* 2 total/4			*V. spicata* 2 west/4	
	Mean 2 (*N* = 40)	Mean 4 (*N* = 65)	*p*-value	Mean 2 (*N* = 74)	Mean 4 (*N* = 69)	*p*-value	Mean 2 (*N* = 33)	*p*-value
**bio2**	8.99	9.58	**0.047**	9.65	8.62	<0.001*	8.42	0.431
**bio3**	25.20	25.61	0.629	25.36	29.00	<0.001*	29.10	0.894
**bio7**	36.28	38.89	0.185	39.35	29.81	<0.001*	29.17	0.383
**bio8**	13.39	16.90	<0.001*	16.20	16.32	0.861	13.68	**0.006**
**bio9**	−5.18	−7.18	0.267	−7.10	0.11	<0.001*	0.93	0.292
**bio10**	15.27	17.26	<0.001*	17.72	17.40	0.365	16.92	0.332
**bio15**	39.48	42.85	0.334	36.48	32.40	**0.031**	27.24	**0.009**
**bio17**	85.88	77.75	0.258	98.34	105.32	0.406	153.31	<0.001*

*The latter was tested with and without the diploid Siberian samples (2 total and 2 west, respectively). The V. spicata mean 4 column is valid for both comparisons. Only the eight uncorrelated bioclim variables are shown here but the test was done for all 19 variables (see Supplementary Table 4). Significant results (p < 0.05) are in bold. Significant results after Bonferroni correction for multiple tests (p < 0.003) have an asterisk.*

**FIGURE 6:**
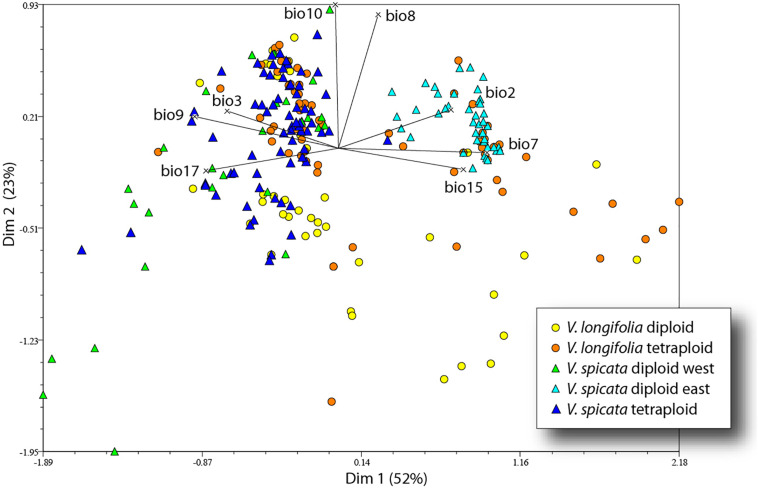
Principal component analysis of *V*. *longifolia* and *V. spicata* based on 248 data points and eight bioclim variables.

The first four axes of the PCA incorporated 52, 23, 11, and 7% of the total variance. The first axis was mainly determined by variables based on seasonality (bio2, bio3, bio7, and bio15) and precipitation (bio9 and bio17), while the second axis was mainly determined by temperature variables (bio8 and bio10). The 41 eastern samples of diploid *V. spicata* (cyan blue triangle in Figure 6) were separate from all other *V. spicata* data points in the PCA. However, the western remainder of diploid *V. spicata* was largely overlapping with tetraploid *V. spicata*, and the same was true for most of the diploid and tetraploid individuals of *V. longifolia*, respectively.

The variables that contributed more than 10% to MAXENT models were: for diploid *V. longifolia* bio7 (43%) and bio17 (28%); for tetraploid *V. longifolia* bio8 (48%), bio 17 (21%), and bio7 (11%); for diploid *V. spicata* (all data) bio17 (41%), bio10 (22%), and bio9 (12%); for diploid *V. spicata* west bio17 (63%) and bio3 (21%), for diploid *V. spicata* east bio7 (34%), bio10 (25%), bio17 (20%), and bio8 (16%); for tetraploid *V. spicata* bio7 (36%) and bio17 (34%). The maps produced by the logistic output of MAXENT revealed slight differences between the conspecific diploid and tetraploid groups of each species (Supplementary Figure 2). The main difference within *V. longifolia* was that the plot for diploids indicated suitable climate also in northern Europe (Scandinavia and northeast of the Baltic Sea), but less so for the tetraploids. Within *V. spicata*, the areas suitable for tetraploids and for the western diploids are very similar. This changed when eastern individuals were also included, adding a large corridor from eastern Europe to western Asia to the potentially suitable conditions of the diploids.

The results of the quantitative niche overlap analysis (Schoener’s *D*) and the niche identity test for all pairwise comparisons indicated significant niche differentiation (Table 5). These analyses recovered the least differentiation between *V. longifolia* tetraploid and *V. spicata* diploid and highest difference between *V. spicata* diploid and *V. spicata* tetraploid.

**TABLE 5:** The results of the niche overlap (Schoener’s *D*) and niche identity tests.

	Schoener’s *D*	Niche identity test mean *D* ± *SD*	*p*-value
*V. longifolia* tetraploid vs. diploid	0.61	0.79 ± 0.04	<0.01
*V. spicata* tetraploid vs. diploid	0.39	0.84 ± 0.03	<0.01
*V. spicata* tetraploid vs. diploid (west)	0.66	0.81 ± 0.04	<0.01
*V. longifolia* tetraploid vs. *V. spicata* diploid	0.69	0.82 ± 0.03	<0.01
*V. longifolia* diploid vs. *V. spicata* diploid	0.51	0.81 ± 0.04	<0.01
*V. longifolia* tetraploid vs. *V. spicata* tetraploid	0.40	0.81 ± 0.04	<0.01
*V. longifolia* diploid vs. *V. spicata* tetraploid	0.41	0.78 ± 0.05	<0.01
*V. longifolia* diploid vs. *V. spicata* diploid (west)	0.46	0.78 ± 0.06	<0.01
*V. longifolia* tetraploid vs. *V. spicata* diploid (west)	0.40	0.77 ± 0.04	<0.01

*The mean, the standard deviation (SD) of the D value, and the p-values were derived from the distribution of D values from 100× pseudoreplicates.*

## Discussion

In this study, we investigated the phylogeographic structure using genome-wide SNPs considering hybridization, introgression and polyploidization of two congeneric species, *V. spicata* and *V. longifolia* across their vast European distribution ranges. Though *V. longifolia* exhibited lower geographic structure, our results demonstrated clear geographical patterns for both species. Hybridization and introgression through backcrossing and polyploidization was detected in several natural populations across the European distribution of both species, but apparently these cases of hybridization have not lead to large-scale merger of the species, neither such hybridization obscured the species boundaries. Finally, experimental crossings revealed high level of compatibility between the two species in cases in which individuals have the same ploidy levels. We have not recovered any clear geographical or macroclimatic patterns related to polyploidy. Thus, the two species do not form hybrid zones on a continental scale but likely have formed hybrid zones of different extent and temporal duration on a local scale ever since the two species split about 1.5 million years ago (Khan et al., submitted).

### Phylogeographic Structure of *V. longifolia* and *V. spicata* Across Eurasia

Both the population genetic structure and maximum likelihood tree indicated a basal split between European and Central to East Asian (plus two European) samples of *V. longifolia* after removal of admixed (*Q* > 0.2 for the minor ancestry) individuals. The higher amount of inferred admixture for these individuals (4–20%; Supplementary Table 1) is not considered to be real evidence for introgression but rather an artifact of STRUCTURE based on genetic divergence of these samples from the majority of (European) individuals. Currently, we lack evidence for this hypothesis but this is currently being analyzed in more detail. Such a split between European and Central to East Asian populations has previously been found in *Clausia* (Franzke et al., 2004) with a split between groups found around 70° East and further west along the Ural Mountains in *Camelina microcarpa* (Žerdoner Čalasan et al., 2019). This split along the Ural Mountains is in line with the division of periglacial steppes in two large ecosystems east and west of the Ural Mountains based on paleo-environmental reconstructions (Hurka et al., 2019). Considering the postulated East Asian origin of the subgenus (Kosachev et al., 2016), we hypothesize that *V. longifolia* originated in Eastern Asia and subsequently dispersed westward toward Europe and further differentiated there. A split between the European and Asian populations in *V. longifolia* has already been suggested by Trávníček (2000), who considered the differences between Asian and European plants substantial enough to treat them as two different species. However, given our sparse sampling among Asian populations and the lack of clear morphological differentiation, we currently refrain from adopting this view.

In contrast to *V. longifolia*, the earliest branching samples in *V. spicata* are from the South Russian – North Caucasian region (Figure 4). This argues against the East Asian origin but rather indicates early differentiation along the southern border of the extant distribution, though not necessarily an origin within that region. The only Asian samples of that species included here were nested among samples from Ukraine, which suggests that *V. spicata* migrated eastwards to the Altai Mountains either from Southern Russia or from a widespread Eurasian steppe group. The results of the STRUCTURE analysis support the latter view that the samples of *V. spicata* from the Altai Mountains and Eastern Europe belong to a group of widespread Eurasian steppe genotypes and the Southern Russian populations belong to a separate population group that differentiated early in the evolution of the species. As in *V. longifolia*, these results show higher amounts of admixture, which we again attribute more to intraspecific genetic divergence than to real introgression from *V. longifolia* (see above). In this case, it is noteworthy that sample sp1x_Rus1 morphologically belongs to *V. spicata* subsp. *transcaucasica* Bordz., which is morphologically differentiated from typical *V. spicata*. The existence of widespread Eurasian steppe genotypes is somehow counter to the more general assumption that many species during glacial phases of the Pleistocene survived in southern refugia, e.g., the three southern European peninsulas with a subsequent spread across Europe and eastwards to Siberia (Taberlet et al., 1998; Hewitt, 2001). This generalized phylogeographic pattern, being in general true for many species, is, however, not considered a universal key suitable for explaining all phylogeographic patterns of European and Eurasian plant species anymore. Several other patterns for taxa of vascular plants have been proposed as well (e.g., Suchan et al., 2019; Friesen et al., 2020; Volkova et al., 2020, and references therein), such as survival in more northern refugia (even cryptic ones: see Stewart and Lister, 2001), ecological shifts with possible survival in habitats and plant communities not now peculiar to the species, etc. (see a paleobotanical overview in Bezusko et al., 2011). These patterns reflect that each species reacted to the Pleistocene and Holocene environmental and climate changes in an idiosyncratic way reflecting that many characters and ecological adaptations are specific to that species. Phylogeographic patterns of Eurasian steppe plants should also be considered against the background of the general development and transformations of the Eurasian steppe zone (see an overview in Hurka et al., 2019, and references therein), especially during the Pleistocene and Holocene climatic and environmental cycles. Thus, an analysis with better sampling across the Eurasian steppe is necessary to identify refugia of these two species of *Veronica*.

The ML phylogram (Figure 4) further suggests that *V. spicata* expanded westwards to Eastern Europe (southern Poland and Ukraine) from the Southern to Central Russian refugium. All individuals west of Ukraine form two geographically distinct groups, one constitutes a Western European (Germany to United Kingdom) and another a Pannonian-Balkanian group. Our TESS3R (Figure 3A) results detected five genetic clusters in *V. spicata*, which form geographically delimited groups mostly in congruence with the ML phylogram. These are (I) the Western European group with individuals from United Kingdom, France, Germany, Switzerland, and Western Austria; (II) Pannonian-Balkanian group that includes Serbian, Romanian, Hungarian, Slovenian, Eastern Austrian, and Southern Polish individuals; (3) the South Russian group with an outlying individual from Poland; (4) a widespread Eurasian group composed by Ukrainian populations including the samples from the Russian Altai; and (5) a rather isolated Swiss individual. Consequently, our results agree with previous analyses and support the existence of a western, a southeastern European, a Caucasian, and an Eastern refugium (Stewart and Lister, 2001).

The single diploid individual (sp7d_Swi2) sampled from population Swi2 constitutes a separate Swiss group in the STRUCTURE analysis. STRUCTURE analysis indicates slight admixture for this *V. spicata* individual, while it shows no morphological similarities with *V. longifolia.* Our NewHybrids analysis indicates that sp7d_Swi2 has a partial probability to be a F_2_ hybrid or a backcross with *V. longifolia*, explaining the uniqueness of this Swiss population. Another alternative for this uniqueness might be that sp7d_Swi2 originates from a separate refugial area. This is in concordance with Trávníček et al. (2004) who mentioned *V. spicata* diploid relicts in Switzerland and other areas of Europe. During the last Pleistocene glaciations, the Alps were covered with glaciers, and many species found refuge in the present-day territory of Italy, which was then free of ice (Lugon-Moulin and Hausser, 2002). After glaciations, many species recolonized the Alps from the South following different migration pathways (e.g., through the Rhodanian pathway; Parisod, 2008). A phylogeographic analysis of *V. spicata* in the Alps seems necessary to resolve this question. However, our study warns that hybridization and introgression from *V. longifolia* needs to be considered in such an analysis.

Intraspecific differentiation as in *V. spicata* is not as clear in *V. longifolia*. This may partly be due to our sampling, but also to the higher connectivity of suitable habitats (*V. longifolia* is considered mainly a river corridor plant; Winter et al., 2008; Figure 3B), which allows high gene flow. Nevertheless, phylogenetic analysis, though hampered by low sampling points in western Asia, indicates that *V. longifolia* spread westward across the Russian steppe, reached first Ukraine (Figure 4), from where it seems to have spread to Fennoscandia independently from the rest of Europe. The remaining samples form two clades, one in Western Europe (France, and Germany) and another from Central to Eastern Europe (from Austria to Ukraine).

### Effects of Polyploidization

The genetic structure of both species seems to be largely unrelated to ploidy levels, since both geographical groups in *V. longifolia* and the European populations in *V. spicata* contain both ploidy levels with some geographically coherent clusters but no clear pattern. Thus, recurrent polyploidization events across the distribution area best explain the pattern, although we cannot locate specific areas where tetraploids originated. The Balkan group of *V. spicata* is constituted only by 4× individuals and the East European group only by 2× individuals, while the other groups have both ploidy levels present. Diploid *V. spicata* have been reported from Bosnia (Fischer, 1974), Bulgaria (Graze, 1933), Serbia (Pustahija et al., 2013), Hungary (Löve, 1977), and Romania (Supplementary Table 2) but they have not been analyzed genetically and could potentially be misidentified or, in the case of our Romanian sample, could be the westernmost extension of the Ukrainian group. It is intriguing that there are no tetraploid individuals of *V. spicata* known in Asia. This can have at least three different explanations somewhat depending on the unknown frequency of polyploidization. First, it might be that tetraploids have not originated there by chance; second, they might not have migrated there by chance; or, third, they might have originated there with similar frequency as in Europe, but the habitats were not suitable. Unfortunately, the ANOVA analysis is inconclusive (Table 4), because significant niche differences within Europe seem mainly due to less precipitation in habitats of tetraploids and higher precipitation in diploids but when looking at the total range of the diploids, the significant differences indicate different temperature regime only. The same pattern can be seen in the PCA (Figure 6) in which the diploid and tetraploid *V. spicata* groups were largely overlapping and only the Siberian diploids were separate from all other *V. spicata* points. Also, the analysis of niche overlap (Table 5) is not a good or valid indicator for putative differences between ploidy levels in our study. Admittedly, there were significant differences between the ploidy levels. This test statistic, however, seems overly sensitive to unequal geographical sampling and we could not adjust our sample to accommodate for this. An example for this problem was, that the measured niche overlap between diploid and tetraploid *V. spicata* was smaller than between any other comparison, even between the different species. In conclusion, Schoener’s *D* test statistic may not be suitable for such a wide and therefore partly incomplete geographical sampling as in our study.

Since tetraploid *V. spicata* have been found mainly in the Pannonian-Balkan region and diploids have rarely been found in the region, it should be investigated in more detail whether the tetraploid *V. spicata* from the region constitute a genetically differentiated infraspecific taxon with higher drought resistance (Table 4), a trait reported for other autopolyploids, although this is not a universal pattern (e.g., Yang et al., 2014). In that respect, the connection between the Pannonian-Balkan tetraploids and the other tetraploids needs to be studied further. Are these tetraploids of different origin, which could not spread as much as the Balkan populations? Despite this question, tetraploid *V. spicata* fits the common scenario found in many other European polyploids that were better able to colonize North European areas after glacier retreats (see Figure 2; blue vs. green points), possibly by being better adapted to cold conditions (Brochmann et al., 2004; but see Tkach et al., 2008), although this is not directly reflected in the comparison of climatic variables shaping ecological niches (e.g., similar bio10 values in Table 4). An alternative explanation for the success of polyploids applicable here is that due to fixed heterozygosity polyploids are able to cope better with inbreeding depression after colonization events in new areas (Birchler, 2012). In contrast, in *V. longifolia* the diploids spread further north, with the tetraploids requiring higher temperature (e.g., bio10 in Table 4). Thus, the macroclimatic niche signal we found is weak and partly inconsistent, but if there was any, it seemed that polyploidization had different effects in the two related species. Finally, our genetic data showed that there was and is a recurrent evolution of polyploids in both species, which produces many unrelated and unconnected autopolyploid lineages. Thus, our explorations of climatic niches fit well to the theory, that the *Veronica* polyploids still carry the macroclimatic niche requirements of their individuals and immediate diploid ancestral population rather than a common, “typical” and detectable polyploid niche requirement.

### Hybridization

The genetic divergence between *V. spicata* and *V. longifolia* was found to be low (*F*_*ST*_ = 0.29), indicating structured populations with connectivity (Supplementary Table 3). This suggests that the amount of evolutionary divergence between the parental species is not high enough to cause significant genetic incompatibilities (Moyle and Nakazato, 2010). Further, the possibility for hybridization to occur was revealed in our crossing experiments, which also demonstrated that plants of the two species can form viable seeds independent of their geographic origin, but only when both parents have the same ploidy level (Table 3). Third, macroclimatic differences between the two species appear minor (Table 5), which is consistent with the coexistence of the two species in close proximity (e.g., Hun6, Ukr13, and Ukr16-20).

Hybridization due to secondary contacts is considered to be a common phenomenon following recolonization after retreat of Pleistocene glaciers (Hewitt, 2001). During these glaciations (glacial phases, in contrast to interglacials), communities of steppe vegetation and its ecological analogs (such as cold and dry periglacial steppes or similar dry grassland plant communities) were favored by predominantly continental climate in most part of Europe, thus possibly providing large and connected open habitats (Mai, 1995; Bezusko et al., 2011; Hurka et al., 2019); such habitats were most probably suitable for *V. spicata* and ensured its survival and dispersal during Pleistocene cycles. On the other hand, interglacial phases of the Pleistocene were normally warmer and more humid in most of Europe and thus promoted such habitats as riparian meadows and floodplain grasslands developing on fluvioglacial deposits, which were more favorable for survival and dispersal of *V. longifolia*. Thus, most probably these two species reacted in different manners to the environmental changes of the Pleistocene and Holocene but shifts in environmental conditions brought them into frequent contact.

Unfortunately, direct paleobotanical (paleopalynological) data on the history of *Veronica* in the Late Quaternary are limited because pollen grains of those entomophilous species are rare in fossil and subfossil pollen samples (see Bezusko et al., 2011, 2018); also, distinguishing species of *Veronica* based solely on their pollen is problematic (Tsymbalyuk and Mosyakin, 2013). However, some paleobotanical (in fact, paleoecological) evidence on our two species can be obtained indirectly, through reconstructions of the possible distribution of ancient plant communities suitable for these species. In such assumptions we should, however, take into considerations possible habitat shifts of species in the past, as well as the existence of plant communities of the past that were only roughly corresponding to modern plant communities of the region concerned (Bezusko et al., 2011). In any case, such paleoreconstructions should be done in parallel and in agreement with experimental and molecular results, such as those presented here.

Our results indicate that hybridization events can be found between *V. spicata* and *V. longifolia* across the whole distribution range in Europe, with tetraploid and diploid hybrid individuals identified in populations from Ukraine, Slovakia, and Switzerland and likely happened for a long time. However, reliable identification of hybrids can be difficult. Here, we adopted a genetic definition based on results of the admixture analysis in combination with phylogenetic evidence. Individuals with higher admixture levels branched earlier among the respective species. Thus, the genetic divergence appears continuous from intraspecific variation to hybrids (Supplementary Table 1), and geographic coherence was added to justify the cut-off.

NEWHYBRIDS results are in line with STRUCTURE (Figures 1, 5). We observed that individuals with high probability to be hybrids or back-crosses with one of the two parental species in the STRUCTURE analysis showed a significant introgression probability proportional to the genotype frequency class expected (Anderson and Thompson, 2002). Overall, 13 individuals had a significant posterior probability to be one of the four hybrid classes (Figure 5). Backcrossing was mainly inferred toward *V. longifolia*, with only one exception, suggesting that introgression is asymmetrical from *V. spicata* to *V. longifolia*. Introgression seems to be not uniform inside natural populations, as indicated by the fact that some individuals resulted to be highly introgressed, while other individuals from the same population (e.g., Ukr19) were not admixed. Furthermore, in natural populations hybridization seems to be a phenomenon not restricted only to tetraploid individuals, even though it seems to occur less frequently in diploids (in only one case the identified hybrid was a diploid compared to five cases among tetraploids). Our crossing results indicate that the vitality of diploid and tetraploid hybrid individuals is comparable, while crossings between different ploidy levels produce seeds with a lower seed development, germination success and survivability, suggesting ploidy level as a crossing barrier. In line with this, hybridization in the two species was only observed in the populations in which *V. spicata* and *V. longifolia* had the same ploidy (Ukr17–20) and not in the populations where the two species differed in their ploidy (Hun6, Ukr13, and Ukr16–18). This result is in accordance with Härle (1932), who suggested within-ploidy but not between-ploidy cross-compatibility. Stebbins (1971) already hypothesized that inter-cytotype gene flow is mostly unidirectional from diploids to tetraploids and this has been demonstrated in the last years with molecular tools (e.g., Jørgensen et al., 2011; Zohren et al., 2016). Whereas documented cases of introgression in the wild involving the same ploidy level are frequent, less common examples between different ploidy levels are known. This is due to the fact that triploid hybrids are not produced (triploid block), and if produced, they are highly sterile (Ramsey and Schemske, 1998). In fact, despite thousands of flow cytometric analyses in *Veronica*, uneven ploidy levels have never been reported in the wild (Bardy et al., 2011, Supplementary Table 1 for *V*. subg. *Pseudolysimachium*, but see also Bardy et al., 2010; Padilla-García et al., 2018; Rojas-Andrés et al., 2020; López-González et al., 2020). However, we produced some triploids in our greenhouse from interploidal crosses (Table 3), although we did not yet grow them to maturity to check for fertility. Thus, in some rare cases, triploid individuals may exist in the wild and functional gametes may be produced by triploid hybrids. This can break ploidy barrier and, through backcross with one of the parental species, result in gene transfer (Chapman and Abbott, 2010).

Thus, as suggested by Bardy et al. (2011), hybridization may have generated a great morphological variation by generating several morphologically intermediates between species. Some hybrids seem to have intermediate morphology, while others seem to be more similar to one of the parents morphologically and/or in term of ecological niche. For example, the individual *V.* × *media* (xm1t_Ukr20), classified as F_2_ hybrid, showed intermediate morphological characters between *V. spicata* and *V. longifolia*, while the other hybrids detected, even those inferred to be F_2_ such as lo37t_Slo2 and lo15t_Ukr2, did not. The predominance of backcrossing to *V. longifolia* combined with the majority of hybrids *a priori* identified as *V. longifolia* suggests that there is selective pressure favoring a *V. longifolia*-phenotype for the hybrids and, thus, backcrossing with *V. longifolia*. However, this selection does not occur up to the seedling stage since we did not observe maternal effects in our crossings (Table 3).

## Conclusion

The genus *Veronica*, with its high species richness and diversification, as well as a great ecological amplitude, constitutes an important model study system which allows investigating several aspects and factors of plant evolution. In the present study, further evidence is provided to support the hypothesis, already proposed for *V.* subg. *Pseudolysimachium* in other regions (Balkans – Bardy et al., 2011; Altai – Kosachev et al., 2019), that hybridization between *V. spicata* and *V. longifolia* is common but these two species remain separate and distinct due to their different ecological adaptations and partial geographical or spatial isolation, with hybrids being probably less competitive than their parents. Furthermore, as shown in the crossing experiment, ploidy can constitute a further reproductive barrier, preventing gene flow between natural populations of different ploidy levels. Our analyses further demonstrate that, despite similar distribution ranges and flower biology, the two species differ markedly in their phylogeographic structure. Dry grasslands appear to have occurred more scattered than riparian habitats with their high connectivity, leading to more elaborate population structure in *V. spicata* as compared to that in *V. longifolia*. Increasing the number of populations sampled and performing sampling in a more geographically homogeneous way across Eurasia may provide interesting and more solid data on the origins and phylogeographic histories of the two species and their evolutionary interactions. Further ecological studies in different regions would be necessary to evaluate the effective ecological competitiveness of the hybrids compared with their parents and to identify which ecological niches they can occupy. The ecology of the hybrids is totally unknown; it can be intermediate between parents, but also one of the two extremes.

## Data Availability Statement

The datasets generated for this study can be found in online repositories. The names of the repository/repositories and accession number(s) can be found below: 10.5061/dryad.kkwh70s3.

## Author Contributions

DA, PK, SM, and GK conceived the idea. DB and EM-Q performed the experiments. DB, KBH, and GK conducted formal analyses. PK, EM-Q, and DA contributed reagents, materials, and analysis tools. DA, GK, DB, PK, and SM authored the drafts of the article. All authors contributed, made multiple revisions, and approved the final draft.

## Conflict of Interest

The authors declare that the research was conducted in the absence of any commercial or financial relationships that could be construed as a potential conflict of interest. The handling editor declared a past co-authorship with one of the authors SM.

## Supplementary Material

Supplementary Figure 1 Phylogenetic analysis based on maximum likelihood statistics as implemented in RAxML including hybrids. Coloring and labeling follow Figure 4. The IDs assigned to the samples (e.g., lo82t_Ukr17) are constructed using the first two letter to identify the species, e.g., lo for *Veronica longifolia*, sp for *V*. *spicata*, and sc for *V*. *schmidtiana*, followed by a unique number for every individual and a letter to indicate the ploidy level (*d* for 2×, *t* for 4×, and × for unknown ploidy). Underscore separates the information related with population constituted by 3 letters to identify the country (e.g., CzR for Czechia) and a unique number in that species (see complete detail in Supplementary Table 1).

Supplementary Figure 2 Macroclimatic maps resulting from the logistic output of MAXENT (Phillips et al., 2019). Macroclimatic suitability of regions for the groups analyzed ranges from very high (red) to poor (blue). White squares indicate points used for training the model. **(A)** Diploid *V. longifolia*. **(B)** Tetraploid *V. longifolia*. **(C)** All of diploid *V. spicata*. **(D)** Tetraploid *V. spicata*. **(E)** Only western individuals of diploid *V. spicata*. **(F)** Only eastern individuals of diploid *V*. *spicata*.
