# Supplementary figures and images for: Comparative Phylogeography of Veronica spicata and V. longifolia (Plantaginaceae) Across Europe: Integrating Hybridization and Polyploidy in Phylogeography
Source: Front Plant Sci. 2021 Feb 1;11:588354. doi: 10.3389/fpls.2020.588354 (PMC7884905; doi:10.3389/fpls.2020.588354)

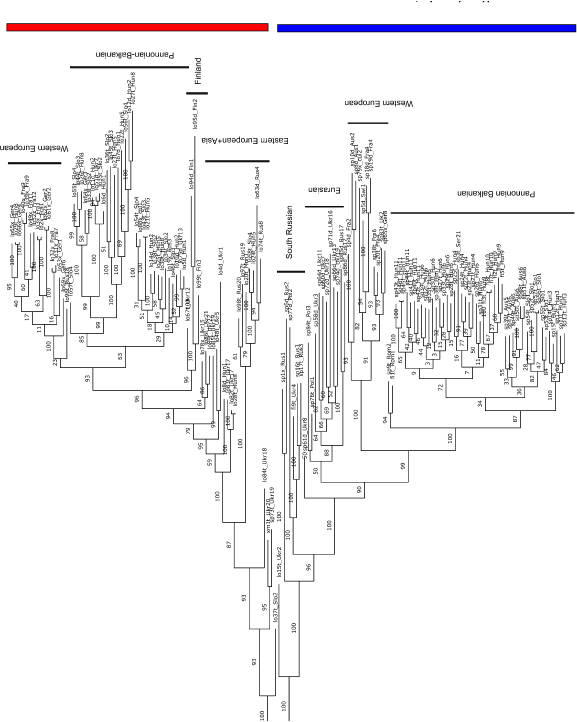

Supplement: Supplementary Figure 1 — Phylogenetic analysis based on maximum likelihood statistics as implemented in RAxML including hybrids. Coloring and labeling follow Figure 4. The IDs assigned to the samples (e.g., lo82t_Ukr17) are constructed using the first two letter to identify the species, e.g., lo for Veronica longifolia, sp for V. spicata, and sc for V. schmidtiana, followed by a unique number for every individual and a letter to indicate the ploidy level (d for 2×, t for 4×, and × for unknown ploidy). Underscore separates the information related with population constituted by 3 letters to identify the country (e.g., CzR for Czechia) and a unique number in that species (see complete detail in Supplementary Table 1). [file Image_1.TIF]

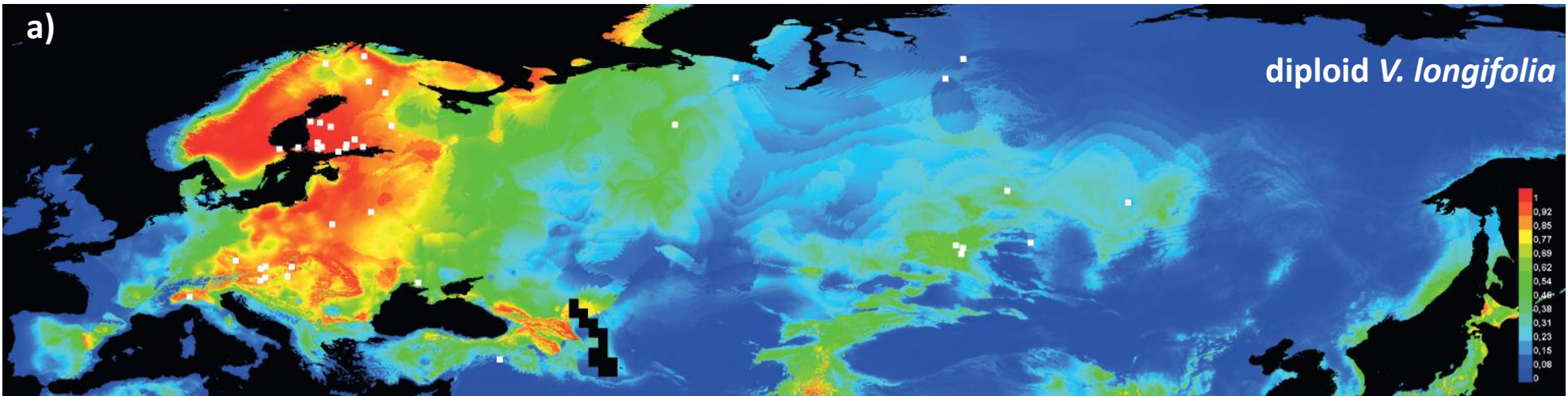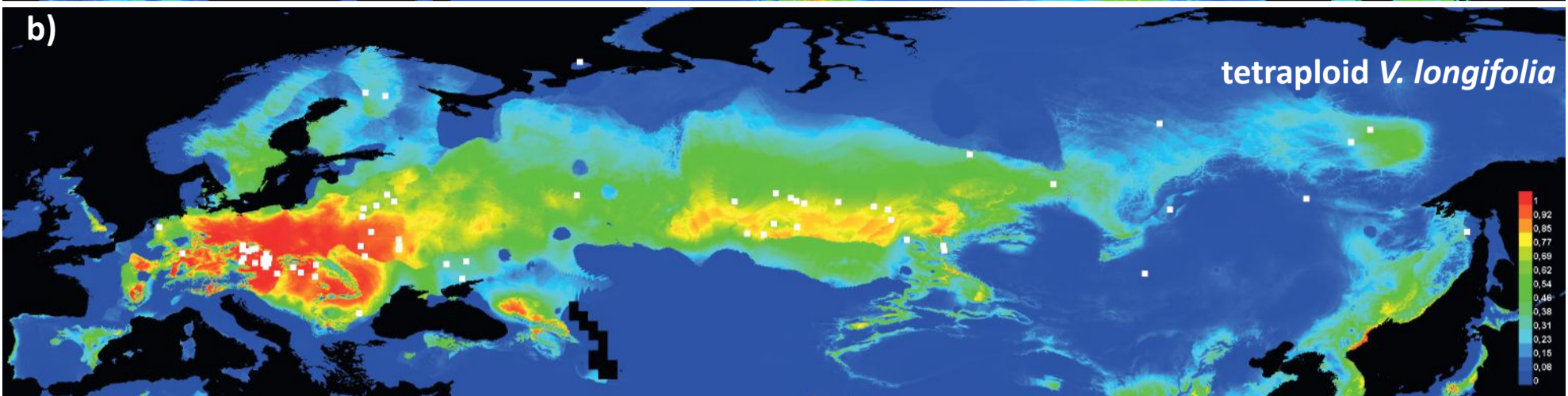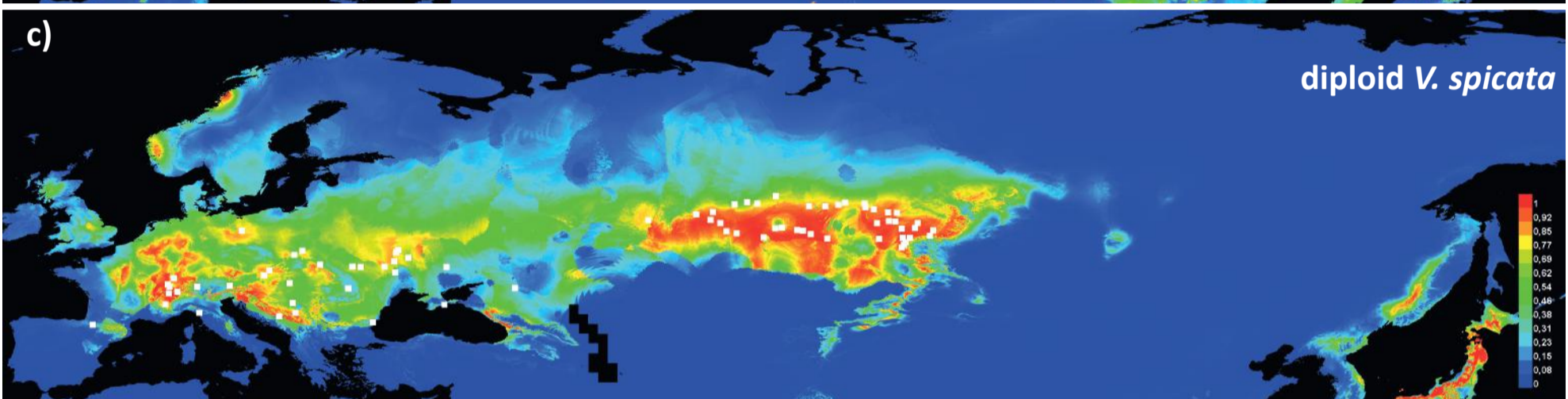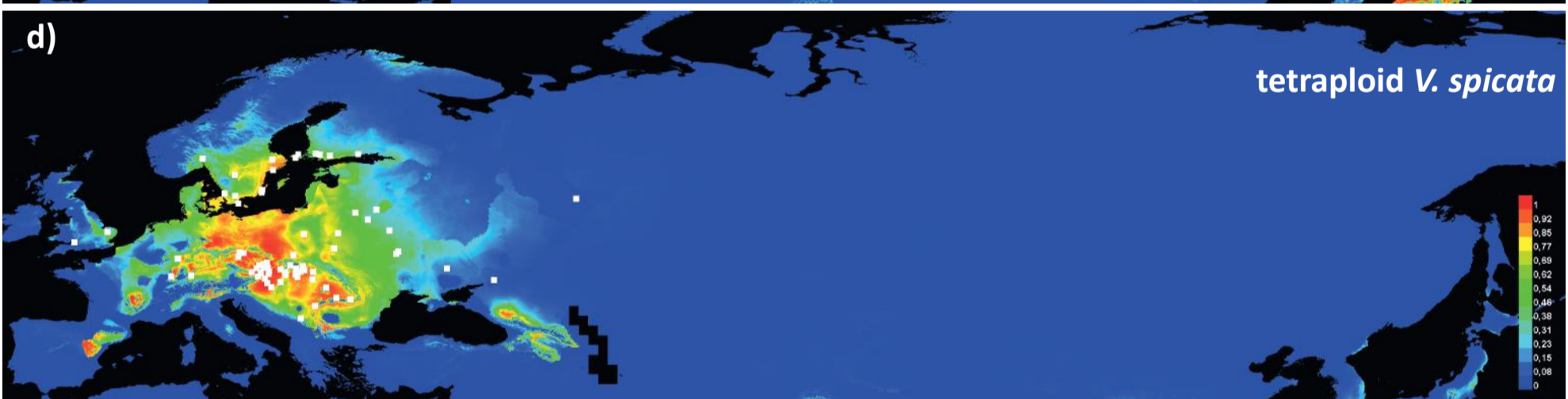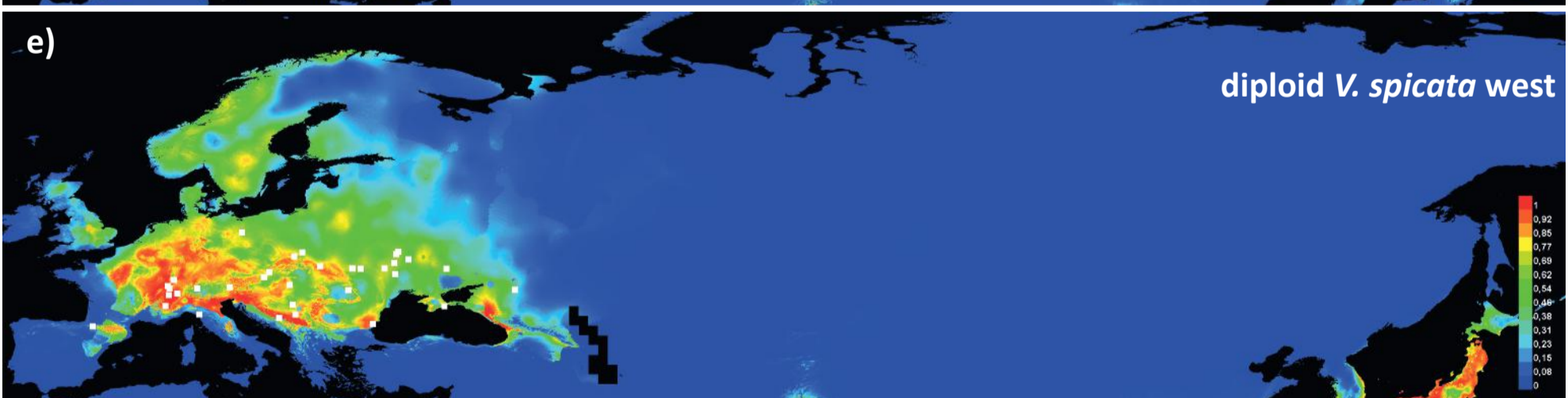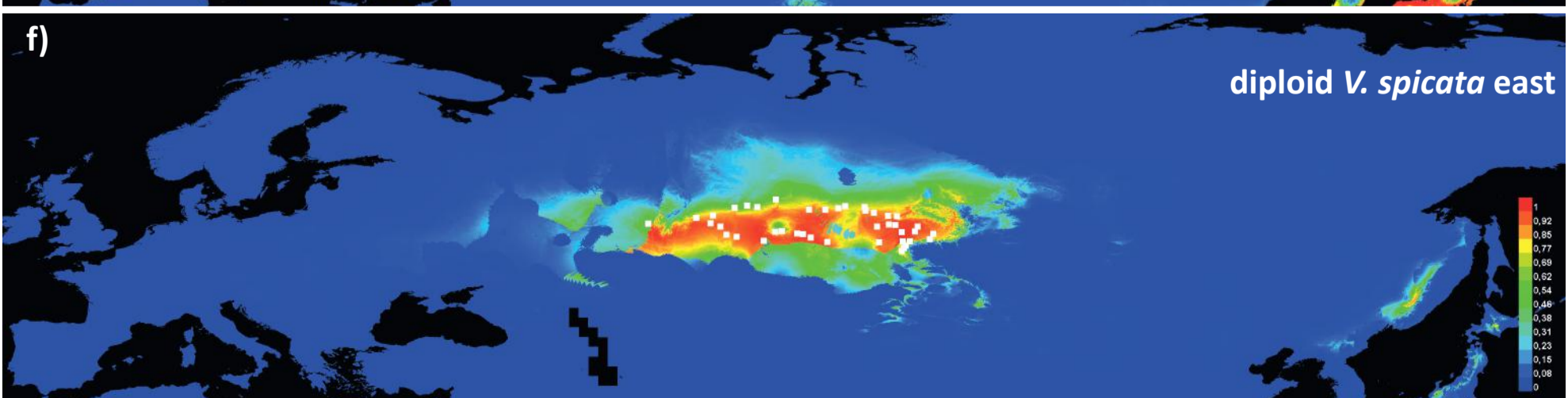

Supplement: Supplementary Figure 2 — Macroclimatic maps resulting from the logistic output of MAXENT (Phillips et al., 2019). Macroclimatic suitability of regions for the groups analyzed ranges from very high (red) to poor (blue). White squares indicate points used for training the model. (A) Diploid V. longifolia. (B) Tetraploid V. longifolia. (C) All of diploid V. spicata. (D) Tetraploid V. spicata. (E) Only western individuals of diploid V. spicata. (F) Only eastern individuals of diploid V. spicata. [file Image_2.PDF]
