# Supplementary material for: Comparative Phylogeography of Veronica spicata and V. longifolia (Plantaginaceae) Across Europe: Integrating Hybridization and Polyploidy in Phylogeography
Source: Front Plant Sci. 2021 Feb 1;11:588354. doi: 10.3389/fpls.2020.588354 (PMC7884905; doi:10.3389/fpls.2020.588354)
Supplement: Supplementary file 3 [file Data_Sheet_1.docx]

library(vcfR)

# load the original VCF file obtained with Stacks 2.4

orig.VCF <- read.vcfR("vcf_files/r08/veronica_r08_filt.vcf.gz")

orig.VCF

# censoring data (scoring as NA some variants) according to dp.

# 1) Variants sequenced at low coverage may only observe one of two alleles in a diploid.

# 2) Variants sequenced at high coverage may be from repetitive regions that were assembled

# in our reference as a single region. This means that different alleles may be from

# different copied (loci)

dp <- extract.gt(orig.VCF, element = "DP", as.numeric=TRUE)

options(bitmapType='cairo')

png("heatmap_original.png")

heatmap.bp(dp[1:100000,], rlabels = FALSE) # print ony a part [1:100000] of the info about genotypes

dev.off()

dp[dp < 8] <- NA

dp[dp > 800] <- NA

max(dp, na.rm=T)

min(dp, na.rm=T)

png("heatmap_rem_less_8_more_800_dp.png")

heatmap.bp(dp[1:100000,], rlabels = FALSE) # print only a part [1:100000] of the info about genotypes

dev.off()

# omitting variants ####

myMiss <- apply(dp, MARGIN = 1, function(x){ sum( is.na(x) ) } )

myMiss <- myMiss / ncol(dp)

VCF.2<-orig.VCF

VCF.2@gt[,-1][ is.na(dp) == TRUE ] <- NA # update the vcfR object

VCF.2

VCF.2 <- VCF.2[myMiss < 0.7, ]

VCF.2

dp2 <- extract.gt(VCF.2, element = "DP", as.numeric=TRUE)

png("heatmap_rem_variants_more_70miss.png")

heatmap.bp(dp2, rlabels = FALSE)

dev.off()

range(dp2,na.rm=T)

# omitting samples after removing variants ####

myMiss2 <- apply(dp2, MARGIN = 2, function(x){ sum( is.na(x) ) } )

myMiss2 <- myMiss2 / nrow(dp2)

min(myMiss2)

max(myMiss2)

png("perc_missing_on_samples_after_omitting_variants.png", width = 1400, height = 480)

barplot(myMiss2, main="% missing data per sample after omitting variants", las = 3, cex.names=0.5, col=c("grey", "black"), ylim = c(0,1), xpd=F)

lines(x=c(0:245),y=rep(0.8,246), col="red")

text(x=1,y=0.8,pos=2,label = "0.8", col="red", cex = 0.7)

lines(x=c(0:245),y=rep(0.5,246), col="blue")

text(x=1,y=0.5,pos=2,label = "0.5", col="blue", cex = 0.7)

lines(x=c(0:245),y=rep(0.7,246), col="orange")

text(x=1,y=0.7,pos=2,label = "0.7", col="orange", cex = 0.7)

lines(x=c(0:245),y=rep(0.6,246), col="green")

text(x=1,y=0.6,pos=2,label = "0.6", col="green", cex = 0.7)

dev.off()

VCF.2@gt <- VCF.2@gt[, c(TRUE, myMiss2 < 0.8)] # exclude samples with more than 80% of missing data

orig.VCF

VCF.2

dp3 <- extract.gt(VCF.2, element = "DP", as.numeric=TRUE)

png("heatmap_rem_indiv.png",width = 1480, height = 1480)

heatmap.bp(dp3, rlabels = FALSE)

dev.off()

myMiss3 <- apply(dp3, MARGIN = 2, function(x){ sum( is.na(x) ) } )

myMiss3 <- myMiss3 / nrow(dp3)

min(myMiss3)

max(myMiss3)

png("perc_missing_removing_variants_and_individuals.png",width = 1480, height = 480)

barplot(myMiss3, main="% missing data after omitting variants and samples", las = 3, cex.names=0.5, col=c("grey", "black"), ylim = c(0.0,0.92), xpd=F)

lines(x=c(1:250),y=rep(0.8,250), col="red")

text(x=-4,y=0.7,pos=3,label = "0.8", col="red", cex = 0.7)

dev.off()

# renaming samples to easily identify them

sample.data <- read.table("ref_data_samples.csv", sep="\t", header=T,row.names = NULL)

new_IDs<-character()

for (i in colnames(VCF.2@gt)[-1]){ new_IDs<-c(new_IDs, as.character(sample.data[sample.data$sample.names==i,]$ID_VCF))}

# I renamed once again the sample names because too long, the code used was:

#sample.data <- read.table("ref_data_samples_2.csv", sep="\t", header=T,row.names = NULL)

#library(vcfR)

#VCF.2<-read.vcfR("r06_mis/vcf_files/veronica_r06_filt.vcf.gz")

# colnames(VCF.2@gt)[-1]

# new_IDs<-character()

# for (i in colnames(VCF.2@gt)[-1]){ new_IDs<-c(new_IDs, as.character(sample.data[sample.data$ID_VCF==i,]$short_ID_VCF))}

new_IDs

colnames(VCF.2@gt)[-1]<-new_IDs

colnames(VCF.2@gt)[-1]

write.vcf(VCF.2, file = "veronica_r08_filt.vcf.gz") #save final vcf file

#plot again barplot of missing data per individual with new names

dp3 <- extract.gt(VCF.2, element = "DP", as.numeric=TRUE)

myMiss3 <- apply(dp3, MARGIN = 2, function(x){ sum( is.na(x) ) } )

myMiss3 <- myMiss3 / nrow(dp3)

min(myMiss3)

max(myMiss3)

png("perc_missing_removing_variants_and_individuals.png",width = 1480, height = 480)

barplot(myMiss3, main="% missing data after omitting variants and samples", las = 3, cex.names=0.7, col=c("grey", "black"), ylim = c(0.0,0.92), xpd=F)

lines(x=c(1:250),y=rep(0.8,250), col="red")

text(x=-4,y=0.7,pos=3,label = "0.8", col="red", cex = 0.7)

dev.off()
