# Supplementary material for: Comparative Phylogeography of Veronica spicata and V. longifolia (Plantaginaceae) Across Europe: Integrating Hybridization and Polyploidy in Phylogeography
Source: Front Plant Sci. 2021 Feb 1;11:588354. doi: 10.3389/fpls.2020.588354 (PMC7884905; doi:10.3389/fpls.2020.588354)
Supplement: Supplementary file 4 [file Data_Sheet_2.docx]

library(tess3r)

# bragraph STRUCTURE-like ####

# loading qmatrix obtained from STRUCTURE

setwd("50miss_STRUCTURE_clumped_results")

# q_matrix are the clumped results of several replicates of STRUCTURE (20 Ã longifolia and spicata)

# results for longifolia only are in files:

# K=2: lon_50miss_K2_CLUMPED.txt

# K=3: lon_50miss_K3_CLUMPED.txt

# K=4: qMatr_lon50miss_K4

# results for spicata are in files:

# K=2: qMatr_lonspixm_50miss_K2_clumped.txt

# K=3: qmatr_spi_50miss_K3_CLUMPED.txt

# K=4: qMatr_spi_50miss_K4

# K=5: qMatr_spi_50miss_K5.txt

q_matrix_K_n<-read.table("qMatr_lon50miss_K4",sep="\t")

# sample.data contains informations related to samples (as ID and coordinates):

# longifolia: lon_50miss_unl_noProb_sampleData.csv

# spicata: spi_50miss_unl_noProb_sampleData.csv

# spicata AND longifolia together: sample_data_lonspixm_50miss_unl.csv

sample.data<-read.table("lon_50miss_unl_noProb_sampleData.csv",sep=" ", header=T)

# those next two values (# of rows) has to be identical!

nrow(q_matrix_K_n)

nrow(sample.data)

row.names(q_matrix_K_n)<-sample.data$new_id

row.names(q_matrix_K_n)

cols <- c("#D95F02","#A6761D","#666666","#7570B3","#66A61E","#E6AB02","#1B9E77","#E7298A")

#cols <- c("#1B9E77","#E7298A")

x<-barplot(t(q_matrix_K_n), col = cols, border = F, space = 0,

xlab = "Individuals", ylab = "Admixture coefficients", names.arg = row.names(q_matrix_K_n), axisnames = F #cex.names = NULL#names.arg=sample.data$new_id, angle=90

)

text(cex=0.5, x=x+.9, y=-0.01, row.names(q_matrix_K_n), xpd=TRUE, srt=90, pos=2)

# order by probability K=2

q_matrix_ord<-q_matrix_K_n[order(q_matrix_K_n$V1),]

# order by probability K=3

q_matrix_ord<-q_matrix_ord[order(-q_matrix_ord$V4),]

x<-barplot(t(q_matrix_ord), col = cols, border = F, space = 0,

xlab = "", ylab = "Admixture coefficients", main="clumped K=2 STRUCTURE results 20 rep.\nunlinked SNPs - 25.73% missing data",

names.arg = row.names(q_matrix_ord), axisnames = F #cex.names = NULL#names.arg=sample.data$new_id, angle=90

)

text(cex=0.8, x=x+.9, y=-0.01, row.names(q_matrix_ord), xpd=TRUE, srt=90, pos=2)

# select pure individuals (ind. with less than 20% admixture) ####

# NOTE: make sense only when dataset with 2 species is loaded

q_matr_pure_spi<-q_matrix_ord[q_matrix_ord[,1]>0.8,]

q_matr_pure_lon<-q_matrix_ord[q_matrix_ord[,2]>0.8,]

pure_spi_ids<-row.names(q_matr_pure_spi)

pure_lon_ids<-row.names(q_matr_pure_lon)

pure_spi_ids

pure_lon_ids

# map ####

# to plot sample position

coord = sample.data[,c(14,13)]

plot(coord,

ylab="Latitude", xlab="Longitude",

xlim=c(min(coord$longitude)-2,max(coord$longitude)+2),

ylim=c(min(coord$latitude)-2,max(coord$latitude)+2)

#xlim=c(-3,150),ylim=c(42,68)

)

text(coord,labels = sample.data$new_id, pos = 3,cex=.8)

# tess3r map plot ####

q_matrix_K_n<-tess3r::as.qmatrix(q_matrix_K_n)

plot(q_matrix_K_n, coord, method = "map.max",

#interpol = FieldsKrigModel(10),

main = "Genetic clusters",

xlab = "Longitude", ylab = "Latitude",

resolution = c(600,300), cex = .4

#col.palette = cols

)

text(coord,labels = row.names(q_matrix_K_n), pos = 3,cex=.8)

# map with average values for populations ####

q_matrix_K_n<-read.table("qMatr_spi_50miss_K5.txt",sep="\t")

sample.data<-read.table("spi_50miss_unl_noProb_sampleData.csv",sep=" ", header=T)

nrow(q_matrix_K_n)

nrow(sample.data)

colnames(q_matrix_K_n)<-c("G1","G2","G3","G4","G5")

samples_info<-cbind(sample.data,q_matrix_K_n)

library(plyr) # for ddply

pop_K_vals<-ddply(samples_info, .(population, latitude, longitude),

summarize,

N_spi=sum(species=="spicata"),

N_lon=sum(species=="longifolia"),

N_sch=sum(species=="schmidtiana"),

mixed=if(N_spi>0 & N_lon>0){"lon_spi"}else{if(N_spi>0){"spi"}else{if(N_lon>0){"lon"}else{"sch"}}},

avg_G1=mean(G1),

avg_G2=mean(G2),

avg_G3=mean(G3),

avg_G4=mean(G4),

avg_G5=mean(G5)

)

pop_qMatrix=pop_K_vals[,c("avg_G1","avg_G2","avg_G3","avg_G4","avg_G5")]

row.names(pop_qMatrix) <- pop_K_vals$population

q_matrix_K_n<-tess3r::as.qmatrix(pop_qMatrix)

coord<-pop_K_vals[,c("longitude","latitude")]

plot(q_matrix_K_n, coord, method = "map.max",

#interpol = FieldsKrigModel(10),

main = expression("Genetic clusters identified in "*italic("V. spicata")*" populations"),

xlab = "Longitude", ylab = "Latitude",

resolution = c(600,300), cex = .4,

xlim=c(-4,47)

#ylim=c(43,60) # to adjust size and proportions of maps work on xlim and ylim

#col.palette = cols # if you want to use other colors

)

text(coord,labels = row.names(q_matrix_K_n), pos = 4,cex=.8)
