# Supplementary material for: Comparative Phylogeography of Veronica spicata and V. longifolia (Plantaginaceae) Across Europe: Integrating Hybridization and Polyploidy in Phylogeography
Source: Front Plant Sci. 2021 Feb 1;11:588354. doi: 10.3389/fpls.2020.588354 (PMC7884905; doi:10.3389/fpls.2020.588354)
Supplement: Supplementary file 5 [file Data_Sheet_3.docx]

# elaboration script on filtered VCF data from Veronica individuals

library(vcfR)

library(poppr)

library(ape)

library(RColorBrewer)

library(adegraphics)

lonspi.filtered.VCF <- read.vcfR("lonspischxm_50miss_filt2.VCF.gz")

load(file="gl.lonspi.filt.dip.Rdata")

load(file="gl.lonspi.filt.tet.Rdata")

load(file="gl.lonspi.filt.plo.Rdata")

load(file="gl.lonspi.filt.Rdata")

load(file="gl.lon.filt.Rdata")

load(file="gl.spi.filt.Rdata")

load(file="gl.lonspi.only.filt.Rdata")

load(file="gl.lonspisch.only.Rdata")

#lonspi.filtered.VCF <- read.vcfR("lonspi_filtered_1.vcf.gz")

#lonspi.filtered.VCF <- read.vcfR("lonspi_filt_1_biallelic.vcf")

# check for LEA

#lon.spi.str<-read.table("lon_spi_filt_1.str")

#colnames(lonspi.filtered.VCF@gt)[-1]

# try this with structure and fast structure (gl2faststructure)

#gl2structure(gl, indNames = NULL, addcolumns = NULL, ploidy = 2,

# exportMarkerNames = TRUE, outfile = "gl.str", outpath = tempdir(),

# v = 1)

sample.data <- read.table("sample_data.csv", sep="\t", header=T,row.names = NULL)

sample.data <- read.table("sample_data_filt_noProb.csv", sep=" ", header=T,row.names = NULL)

sample.data <- read.table("sampleData_lonspi_only_50miss_noProb.csv", sep=" ", header=T,row.names = NULL)

sample.data <- read.table("sample_data_lonspischxm_50miss_unl.csv", sep=" ", header=T,row.names = NULL)

gl.lonspi.filt$ind.names

nrow(sample.data)

# just to check if the order of id_vcf is the same for sample.data file and vcf file

identical(as.character(sample.data$new_id),colnames(lonspi.filtered.VCF@gt)[-1])

identical(as.character(sample.data$id_vcf),colnames(lonspi.filtered.VCF@gt)[-1])

for (i in 1:nrow(sample.data)){

if(as.character(sample.data$new_id[i]) != colnames(lonspi.filtered.VCF@gt)[i+1])

cat("a different guy here:", sample.data$new_id[i], colnames(lonspi.filtered.VCF@gt)[i+1],"\n")

else

cat(i,"is fine\n")

}

#sample.data.filtered <- read.csv("sample_data_filt_1_unk_pl_3.txt", sep="\t", header=T, row.names=F)

id_vcf<-colnames(lonspi.filtered.VCF@gt)[-1]

length(id_vcf)

ind.dif<- setdiff(sample.data$id_vcf,colnames(lonspi.filtered.VCF@gt)[-1])

length(ind.dif)

# exclude from sample.data individuals that have been filtered out

sample.data.filt<-sample.data[sample.data$new_id %in% colnames(lonspi.filtered.VCF@gt)[-1],]

sample.data.filt<-sample.data[sample.data$new_id %in% colnames(lonspi.2.VCF@gt)[-1],]

nrow(sample.data.filt)

sample.data<-sample.data.filt

# sample.data.filt<-matrix(ncol=ncol(sample.data))

# dim(sample.data.filt)

#

# #sample.data.filt<-sample.data

# for (i in 1:nrow(sample.data)){

# if(!is.element(sample.data[i,]$id_vcf, ind.dif)){

# cat(i,"added")

# print(sample.data[i,]$id_vcf)

# sample.data.filt<-sample.data.filt[-i,]

# sample.data.filt<-rbind(sample.data.filt,sample.data[i,])

# }

# }

#

sample.data.filt

dim(sample.data.filt)

View(sample.data.filt)

View(sample.data)

# to reduce the number of levels on the current one

sample.data.filt$species<-as.character(sample.data.filt$species)

sample.data.filt$species<-as.factor(sample.data.filt$species)

levels(sample.data.filt$species)

write.table(sample.data,file="sample_data_filt.csv",row.names = F)

#cols <- brewer.pal(n = nlevels(sample.data.filt$species), #number of different colors in the palette

# name = "Dark2") # palette name, for list: brewer.pal.info

#to keep the same color as in excell file

cols <- c("#D95F02","#7570B3","#1B9E77","#E7298A")

cols <- c("#D95F02","#A6761D","#666666","#7570B3","#66A61E","#E6AB02","#1B9E77","#E7298A")

all(sample.data$id_vcf == colnames(lonspi.filtered.VCF@gt)[-1]) #check that all the samples in vcf correspond to the sample.data file

all(sample.data.lonspischxm$new_id == colnames(lonspischxm.VCF@gt)[-1]) #check that all the samples in vcf correspond to the sample.data file

setdiff(colnames(lonspi.filtered.VCF@gt)[-1],as.character(sample.data.filt$id_vcf))

colnames(lonspi.filtered.VCF@gt)[-1]<-as.character(sample.data$new_id)

colnames(lonspi.filtered.VCF@gt)[-1]

write.vcf(lonspi.filtered.VCF, file = "lonspi_50miss_filt2_renam.vcf.gz")

# from VCF to GENLIGHT ####

gl.lonspi.filt <- vcfR2genlight(lonspi.filtered.VCF, n.cores=4)

pop(gl.lonspi.filt) <- sample.data$population

gl.lonspi.filt$strata <- sample.data

levels(sample.data$species)

gl.lonspi.filt

save(file="gl.lonspischxm.Rdata",gl.lonspi.filt)

# genlight to NewHybrids ####

#install.packages("dartR")

library(dartR)

load(file="gl.lonspischxm.Rdata")

gl.lonspixm<-gl.lonspischxm[gl.lonspischxm@strata$species != "schmidtiana"]

gl.lonspixm@strata$species<-as.character(gl.lonspixm@strata$species)

gl.lonspixm@strata$species<-as.factor(gl.lonspixm@strata$species)

gl.lonspixm@strata$species

# population = species

non_pure_ind<-c("lo15t_Ukr2","lo37t_Slo2","xm1t_Ukr20","lo84t_Ukr18","sp73t_Ukr19",

"lo65x_Ger4","lo69x_Ger6","lo39x_Fra9","lo53x_Ger1","lo48t_Ukr5","lo98t_Rus20","lo24t_Hun5","lo85t_Ukr19","lo58t_Slo4","lo28t_Hun8","lo20t_Mon1","lo97t_Rus19","lo82t_Ukr17","lo74t_Rus8","lo63d_Rus4","lo8d_Hun1","lo18x_Fra5","lo83t_Ukr17",

"sp1x_Rus1","sp77d_Pol2","sp6d_Rus2","sp17t_Rus3","fi9t_Ukr4","sp61d_Ukr8","sp76t_Pol1","sp16t_Rus3","sp20t_Fra4","sp12t_Hun3","sp11t_Hun3","sp50t_Aus6","sp13t_Hun3")

# obtained with structure resutls: non_adm_inds<-c(row.names(q_matrix_ord)[1:40],row.names(q_matrix_ord)[116:156])

non_adm_inds<-c("lo54t_Slo4", "lo43t_Slo3", "lo21t_Hun5", "lo10d_Hun2", "lo75d_Ukr13", "lo33t_Slo2" ,

"lo44t_Slo3", "lo56t_Slo4", "lo22t_Hun5", "lo27t_Hun8" , "lo7d_Hun1" , "lo57t_Slo4" ,

"lo12d_Hun2", "lo23t_Hun5", "lo46t_Slo3", "lo29t_Hun8", "lo25t_Hun5", "lo30t_Hun8",

"lo14d_Hun2", "lo88x_cul1", "lo89t_Ukr22", "lo34t_Slo2" , "lo64x_Ger3" , "lo36t_Slo2" ,

"lo45t_Slo3", "lo38t_Slo2" ,"lo78x_Ukr15", "lo66x_Ger4" , "lo2x_Fra1" , "lo55t_Slo4" ,

"lo26t_Hun8" , "lo95d_Fin2", "lo86t_Ukr21" ,"lo49x_Fra11", "lo5d_Hun1" , "lo94d_Fin1" ,

"lo3x_Fra1" , "lo11d_Hun2", "lo67t_Ukr12" ,"lo32x_Fra8" ,"sp60d_Ukr7", "sp41t_Hun11",

"sp84t_Pol3" , "sp18d_Fra4", "sp5d_Swi1" ,"sp23t_Hun4" , "sp43t_Hun11", "sp44t_Hun11",

"sp45t_Hun11" ,"sp27t_Hun6", "sp31t_Hun6" ,"sp34t_Hun6" , "sp54t_Slo1", "sp56t_Slo1" ,

"sp65x_Ger8" , "sp47t_Aus6", "sp48t_Aus6" ,"sp51t_Aus6" , "sp3t_Ser21" , "sp9t_Rom2" ,

"sp79x_cul2" , "sp19d_Fra4" , "sp28t_Hun6" , "sp53t_Slo1" , "sp4d_Fra2" , "sp10d_Aus2" ,

"sp24t_Hun4" , "sp46t_Hun11" ,"fi7t_Hun10" ,"fi2t_Hun9" , "fi4t_Hun9" , "fi8t_Hun10" ,

"sp36t_Hun7" , "sp26t_Hun6" , "sp29t_Hun6" ,"sp32t_Hun6", "sp35t_Hun6" ,"sp49t_Aus6",

"fi5t_Hun9" , "sp57t_Slo1", "sp39t_Aus5")

ind_species<-gl.lonspixm@strata$species

ind_species<-as.character(ind_species)

for(i in 1:length(ind_species)){

if(!gl.lonspixm[i]@strata$new_id %in% non_adm_inds){

ind_species[i]<-"admixed"

}

}

gl.lonspixm2<-gl.lonspixm

pop(gl.lonspixm2)<-as.factor(ind_species)

gl.nhybrids(gl.lonspixm2, outfile = "newHybrids_200AvgPICSNPs2_lonspixm_50miss.txt", p0 = "longifolia",#list of populations to be regarded as parental population 0 [default NULL]

p1 = "spicata",

method = "AvgPIC" #specifies the method (random or AvgPIC) to select 200 loci for NewHybrids [default random]

)

#

# gl.nhybrids(gl.lonspixm, outfile = "lonspixm_50miss.txt", p0 = NULL,#list of populations to be regarded as parental population 0 [default NULL]

# p1 = NULL, t = 0, #â sets the level at which a gene frequency difference is considered to be fixed [default 0]

# method = "random", #specifies the method (random or AvgPIC) to select 200 loci for NewHybrids [default random]

# nhyb.directory = NULL,

# BurnIn = 10000, sweeps = 10000, GtypFile = "TwoGensGtypFreq.txt",

# AFPriorFile = NULL, PiPrior = "Jeffreys", ThetaPrior = "Jeffreys",

# v = 2)

# specify ploidy level ####

#check this package for estimate ploidy level: gbs2ploidy

# next is a trick to remove NA ploidy levels (the NAs will be level 3)

ploid.unk.3<-sample.data$ploidy

ploid.unk.3[is.na(ploid.unk.3)]<-3

ploidy(gl.lonspi.filt)<-ploid.unk.3

# remove NA ploidy (set as 3)

gl.lonspi.filt.plo<-gl.lonspi.filt[gl.lonspi.filt$ploidy!=3] # in sample.data file NAs ploidy were set to 3

ploidy(gl.lonspi.filt.plo)

# lonspi.filt.ploidy.VCF <- lonspi.filtered.VCF[,c(TRUE,sample.data$ploidy != "3")]

# write.vcf(lonspi.filt.ploidy.VCF,"lonspi_20_miss_filt_ploidy.VCF.gz")

gl.lonspi.filt.dip<-gl.lonspi.filt.plo[gl.lonspi.filt.plo$ploidy==2]

ploidy(gl.lonspi.filt.dip)

# lonspi.diploid.VCF <- lonspi.filtered.VCF[,c(TRUE,sample.data$ploidy == 2)]

# write.vcf(lonspi.diploid.VCF,"lonspi.diploid.VCF.gz")

gl.lonspi.filt.tet<-gl.lonspi.filt.plo[gl.lonspi.filt.plo$ploidy==4]

ploidy(gl.lonspi.filt.tet)

# lonspi.tetraploid.VCF <- lonspi.filtered.VCF[,c(TRUE,sample.data$ploidy == 4)]

# write.vcf(lonspi.tetraploid.VCF,"lonspi.tetraploid.VCF.gz")

summary(gl.lonspi.filt@strata$species)

summary(gl.lonspi.filt.plo@strata$species)

summary(gl.lonspi.filt.dip$strata$species)

summary(gl.lonspi.filt.tet$strata$species)

save(gl.lonspi.filt.dip,file="gl.lonspi.filt.dip.Rdata")

save(gl.lonspi.filt.tet,file="gl.lonspi.filt.tet.Rdata")

save(gl.lonspi.filt.plo,file="gl.lonspi.filt.plo.Rdata")

save(gl.lonspi.filt,file="gl.lonspi.filt.Rdata")

load(file="gl.lonspi.filt.Rdata")

# subsetting data by species ####

gl.lon.filt<-gl.lonspi.filt[gl.lonspi.filt@strata$species=="longifolia"]

gl.lon.filt$ind.names

save(gl.lon.filt,file="gl.lon.filt.Rdata")

lon.VCF <- lonspi.filtered.VCF[,c(TRUE,sample.data$species == "longifolia")]

write.vcf(lon.VCF,"lon_50miss_whitelist004.VCF.gz")

sample.data.lon<-sample.data[sample.data$new_id %in% colnames(lon.VCF@gt)[-1],]

nrow(sample.data.lon)

write.table(sample.data.lon,file="sample_data_lon_50miss_whitel004.csv",row.names = F)

gl.spi.filt<-gl.lonspi.filt[gl.lonspi.filt@strata$species=="spicata"]

gl.spi.filt$ind.names

save(gl.spi.filt,file="gl.spi.filt.Rdata")

spi.VCF <- lonspi.filtered.VCF[,c(TRUE,sample.data$species == "spicata")]

write.vcf(spi.VCF,"spi_50miss_whitelist004.VCF.gz")

sample.data.spi<-sample.data[sample.data$new_id %in% colnames(spi.VCF@gt)[-1],]

nrow(sample.data.spi)

write.table(sample.data.spi,file="sample_data_spi_50miss_white004.csv",row.names = F)

gl.lonspi.only<-gl.lonspi.filt[gl.lonspi.filt@strata$species=="longifolia" | gl.lonspi.filt@strata$species=="spicata"]

gl.lonspi.only$ind.names

save(gl.lonspi.only,file="gl.lonspi.only.filt.Rdata")

lonspi.only.VCF <- lonspi.filtered.VCF[,c(TRUE,sample.data$species == "longifolia" | sample.data$species == "spicata")]

write.vcf(lonspi.only.VCF,"lonspi_only_50miss_unlink_whitelist004.VCF.gz")

sample.data.lonspi.only<-sample.data[sample.data$new_id %in% colnames(lonspi.only.VCF@gt)[-1],]

write.table(sample.data.lonspi.only,file="sample_data_lonspi_only_50miss_unl_whitel004.csv",row.names = F)

lonspischxm.VCF <- lonspi.filtered.VCF[,c(TRUE,sample.data$species == "longifolia" | sample.data$species == "spicata" | sample.data$species == "schmidtiana" | sample.data$species == "xmedia")]

write.vcf(lonspischxm.VCF,"lonspischxm_50miss_filt2.VCF.gz")

sample.data.lonspischxm<-sample.data[sample.data$new_id %in% colnames(lonspischxm.VCF@gt)[-1],]

write.table(sample.data.lonspischxm,file="sample_data_lonspischxm_50miss_filt2.csv",row.names = F)

nrow(sample.data.lonspischxm)

gl.lonspischxm<-gl.lonspi.filt[gl.lonspi.filt@strata$species=="spicata" |

gl.lonspi.filt@strata$species=="longifolia" |

gl.lonspi.filt@strata$species=="xmedia" |

gl.lonspi.filt@strata$species=="schmidtiana"]

gl.lonspischxm@strata$species<-as.character(gl.lonspischxm@strata$species)

gl.lonspischxm@strata$species<-as.factor(gl.lonspischxm@strata$species)

levels(gl.lonspischxm@strata$species)

save(file="gl.lonspischxm.Rdata",gl.lonspischxm)

lonspixm.VCF <- lonspi.filtered.VCF[,c(TRUE,sample.data$species == "longifolia" | sample.data$species == "spicata" | sample.data$species == "xmedia")]

write.vcf(lonspixm.VCF,"lonspixm_50miss_unl.VCF.gz")

sample.data.lonspixm<-sample.data[sample.data$new_id %in% colnames(lonspixm.VCF@gt)[-1],]

write.table(sample.data.lonspixm,file="sample_data_lonspixm_50miss_unl.csv",row.names = F)

nrow(sample.data.lonspixm)

# subset with only problematic samples: P2-46 and P2-48, P2-47 and H09clipped_jannes

problematic_samples.VCF <- lonspi.filtered.VCF[,c(TRUE,sample.data$id_vcf == "P2-46_clipped" |

sample.data$id_vcf == "P2-48_clipped" |

sample.data$id_vcf == "P2-47_clipped" |

sample.data$id_vcf == "H09_clipped_jannes")]

colnames(problematic_samples.VCF@gt)[-1]

write.vcf(problematic_samples.VCF,"problematic_samples.VCF.gz")

gl.problematics<-gl.lonspi.filt[gl.lonspi.filt@strata$id_vcf=="P2-46_clipped" |

gl.lonspi.filt@strata$id_vcf=="P2-48_clipped" |

gl.lonspi.filt@strata$id_vcf=="P2-47_clipped" |

gl.lonspi.filt@strata$id_vcf=="H09_clipped_jannes"]

save(gl.problematics,file="gl.problematics.Rfile")

# remove putative hybrids ####

gl.lonspi.only.noHyb<-gl.lonspi.only[gl.lonspi.only@strata$new_id != "sp7d_Swi2" & #sp7d got out already: in unlinked dataset there are more individual due to different levels of missing data

gl.lonspi.only@strata$new_id != "lo15t_Ukr2" &

gl.lonspi.only@strata$new_id != "lo37t_Slo2" &

gl.lonspi.only@strata$new_id != "sp73t_Ukr19" &

gl.lonspi.only@strata$new_id != "lo84t_Ukr18"]

save(gl.lonspi.only.noHyb,file="gl.lonspi.only.noHyb.Rdata")

levels(as.factor(indNames(gl.lonspi.only.noHyb)))

levels(as.factor(indNames(gl.lonspi.only)))

# VCF

lonspi.filtered.VCF<-read.vcfR("lonspischxm_50miss.VCF.gz")

sample.data <- read.table("sample.data.lonspischxm.csv", sep=" ", header=T,row.names = NULL)

identical(as.character(sample.data$new_id),colnames(lonspi.filtered.VCF@gt)[-1])

lonspischxm.noHyb.VCF<-lonspi.filtered.VCF[,c(TRUE, sample.data$new_id != "lo37t_Slo2" & sample.data$new_id !="xm1t_Ukr20" & sample.data$new_id != "sp73t_Ukr19" & sample.data$new_id !="lo84t_Ukr18" & sample.data$new_id != "lo15t_Ukr2")]

levels(sample.data$species)

sample.data$species<-as.character(sample.data$species)

sample.data$species<-as.factor(sample.data$species)

write.vcf(lonspischxm.noHyb.VCF,"lonspischxm_50miss_singletonsH007_noHyb.VCF.gz")

sample.data.lonspisch.noHyb<-sample.data[sample.data$new_id %in% colnames(lonspischxm.noHyb.VCF@gt)[-1],]

write.table(sample.data.lonspisch.noHyb,file="sample_data_lonspischxm_50miss_singletonsH007_noHyb.csv",row.names = F)

# remove problematic samples lo47t, lo81t, sp81t, fi6t, sc1d_Jap1 ####

lonspi.2.VCF <- lonspi.filtered.VCF[,c(TRUE, sample.data$new_id != "lo47t_Slo3" &

sample.data$new_id != "lo81t_Ukr17" &

sample.data$new_id != "sp81t_UK1" &

sample.data$new_id != "fi6t_Hun9" &

sample.data$new_id != "sc1d_Jap1")]

sample.data.2<-sample.data[sample.data$new_id != "lo47t_Slo3" &

sample.data$new_id != "lo81t_Ukr17" &

sample.data$new_id != "sp81t_UK1" &

sample.data$new_id != "fi6t_Hun9" &

sample.data$new_id != "sc1d_Jap1",

]

lonspi.2.VCF <- lonspi.filtered.VCF[,c(TRUE, sample.data$new_id != "sc2d_Jap1")]

sample.data.2<-sample.data[sample.data$new_id != "sc2d_Jap1" ,]

nrow(sample.data.2)

# remove also other samples indicated with other species

write.vcf(lonspi.2.VCF, file = "lonspixm_50miss_unl_ren_noProb.vcf.gz")

write.table(sample.data.2,file="sample_data_lonspixm_50miss_unl_ren_noProb.csv",row.names = F)

# converting to bed ####

library(bedr)

spi.VCF <- read.vcf("spi.VCF.gz") # note read.vcf instead of read.vcfR

lon.VCF<-read.vcf("lon.VCF.gz") # note read.vcf instead of read.vcfR

lon.bed=vcf2bed(lon.VCF,filename="lon.bed.gz")

spi.bed=vcf2bed(spi.VCF, filename = "spi.bed.gz")

# subsetting by variants ####

sub.lonspi.VCF<-lonspi.filtered.VCF[c(1:10),] #first 10 variants

subset.1 <- sample(size = 200, x= c(1:nrow(lonspi.filtered.VCF))) # 200 random variants

sub.lonspi.VCF<-lonspi.filtered.VCF[subset.1,] #subsetting according to a list (random n from 1 to tot_n_variants)

# assess which alleles differentiate more between groups

dapc60PC<-dapc(gi.lonspi.filt, var.contrib = T, #retain variable contributing to the analysis in output

scale=F, n.pca = 60, # 60 PCs from cross-validation

n.da = nPop(gi.lonspi.filt)-1)

scatter(dapc60PC,

col = cols,

cex = 2, legend = TRUE,

clabel = FALSE, posi.leg = "topleft", scree.pca = TRUE,

posi.pca = "bottomleft", cleg = 0.75, xax = 1, yax = 2, inset.solid = 1,cstar=0,lwd=2,lty=2)

set.seed(3)

contrib <- loadingplot(dapc60PC$var.contr,axis = 1, thres = 0.0003, lab.jitter = 1)

length(contrib$var.names)

all_names<-substr(contrib$var.names,1,nchar(contrib$var.names)-2) #remove .0 and .1 from names

un_names<-unique(all_names) #remove duplicate names (e.g. "Contig82_7234457_52531.0" "Contig82_7234457_52531.1")

length(un_names)

gt <- extract.gt(lonspi.filtered.VCF, element = c('GT'), as.numeric = TRUE)

in_list<-rownames(gt) %in% un_names

sub.lonspi.VCF<-lonspi.filtered.VCF[in_list,]

# Fst ####

library(StAMPP)

# load input file in genlight format as converted from VCF and saved previously

load(file="gl.lonspischxm.Rdata")

populations<-pop(gl.lonspischxm)

pop(gl.lonspischxm) <- gl.lonspischxm@strata$species

aa.fst<-stamppFst(gl.lonspischxm, nboots = 100, percent =95, nclusters=4)

###### PCA ###

# PCA problematic samples ####

source(file="custom_functions.R")

pca.filt <-glPcaFast(gl.problematics, center=F, # center means of measurements

nf=4) # function defined in file "custom_functions.R"

#pca.filt <-glPcaFast(gl.lonspi.filt, center=T, # center means of measurements

# nf=4)

# proportion of explained variance by first three axes

ax1 = pca.filt$eig[1]/sum(pca.filt$eig) # proportion of variation explained by 1st axis

ax2 = pca.filt$eig[2]/sum(pca.filt$eig) # proportion of variation explained by 2nd axis

ax3 = pca.filt$eig[3]/sum(pca.filt$eig) # proportion of variation explained by 3rd axis

sum(ax1,ax2,ax3)

barplot(100*pca.filt$eig/sum(pca.filt$eig),

col=heat.colors(50),

main="fast PCA Eigenvalues on filtered data")

title(ylab="Percent of variance\nexplained", line=2)

title(xlab="Eigenvalues", line=1)

# plot different colors for species

# cols <- brewer.pal(n = nlevels(gl.lonspi.filt$strata$species), #number of different colors in the palette

# name = "Dark2")

g1 <- s.class(pca.filt$scores, gl.problematics$strata$species, xax=1, yax=2,

col=cols, ellipseSize=0, starSize=0, ppoints.cex=0.5, paxes.draw=T,

pgrid.draw = F, plab.cex = 0 , plot = FALSE)

g2 <- s.label (pca.filt$scores, xax=1, yax=2, ppoints.col = "red",

plabels = list(box = list(draw = FALSE), optim = T),

ppoints.cex=0.5, paxes.draw=T, pgrid.draw =F, plabels.cex=1, plot = F)

ADEgS(c(g1, g2), layout = c(1, 2))

g2

# PCA spicata and longifolia only ####

source(file="custom_functions.R")

pca.filt <-glPcaFast(gl.lonspi.only, center=F, # center means of measurements

nf=4) # function defined in file "custom_functions.R"

#pca.filt <-glPcaFast(gl.lonspi.filt, center=T, # center means of measurements

# nf=4)

# proportion of explained variance by first three axes

ax1 = pca.filt$eig[1]/sum(pca.filt$eig) # proportion of variation explained by 1st axis

ax2 = pca.filt$eig[2]/sum(pca.filt$eig) # proportion of variation explained by 2nd axis

ax3 = pca.filt$eig[3]/sum(pca.filt$eig) # proportion of variation explained by 3rd axis

sum(ax1,ax2,ax3)

barplot(100*pca.filt$eig/sum(pca.filt$eig),

col=heat.colors(50),

main="fast PCA Eigenvalues\nV. longifolia and V. spicata only")

title(ylab="Percent of variance\nexplained", line=2)

title(xlab="Eigenvalues", line=1)

# plot different colors for species

# cols <- brewer.pal(n = nlevels(gl.lonspi.filt$strata$species), #number of different colors in the palette

# name = "Dark2")

g1 <- s.class(pca.filt$scores, gl.lonspi.only$strata$species, xax=1, yax=2,

col=cols, ellipseSize=0, starSize=0, ppoints.cex=0.8, paxes.draw=T,

pgrid.draw = F, plab.cex = 0 , plot = FALSE)

g2 <- s.label (pca.filt$scores, xax=1, yax=2, ppoints.col = "red",

plabels = list(box = list(draw = FALSE), optim = T),

ppoints.cex=0.5, paxes.draw=T, pgrid.draw =F, plabels.cex=1, plot = F)

ADEgS(c(g1, g2), layout = c(1, 2))

# different colors for ploidy

g1 <- s.class(pca.filt$scores, as.factor(as.vector(ploidy(gl.lonspi.only))),

xax=1, yax=2, col=cols, ellipseSize=0, starSize=0, ppoints.cex=0.8,

paxes.draw=T, pgrid.draw = F, plab.cex = 0 , plot = FALSE)

ADEgS(c(g1, g2), layout = c(1, 2))

title("fast PCA")

# PCA all ####

source(file="custom_functions.R")

ploidy(gl.lonspi.filt)=ploid.unk.3

gl.lonspi.filt@strata=NULL

pop(gl.lonspi.filt)<-NULL

ploidy(gl.lonspi.filt)<-4

gl.lon.filt@strata=sample.data

ploidy<-gl.lonspi.filt@strata$ploidy

ploidy[is.na(ploidy)]<-3

ploidy(gl.lonspi.filt)<-ploidy

pca.filt <-glPcaFast(gl.lonspi.filt, center=F, # center means of measurements

nf=4) # function defined in file "custom_functions.R"

#pca.filt <-glPcaFast(gl.lonspi.filt, center=T, # center means of measurements

# nf=4)

# proportion of explained variance by first three axes

ax1 = pca.filt$eig[1]/sum(pca.filt$eig) # proportion of variation explained by 1st axis

ax2 = pca.filt$eig[2]/sum(pca.filt$eig) # proportion of variation explained by 2nd axis

ax3 = pca.filt$eig[3]/sum(pca.filt$eig) # proportion of variation explained by 3rd axis

sum(ax1,ax2,ax3)

barplot(100*pca.filt$eig/sum(pca.filt$eig),

col=heat.colors(50),

main=paste("fast PCA Eigenvalues on all individuals"))

title(ylab="Percent of variance\nexplained", line=2)

title(xlab="Eigenvalues", line=1)

# plot different colors for species

# cols <- brewer.pal(n = nlevels(gl.lonspi.filt$strata$species), #number of different colors in the palette

# name = "Dark2")

g1 <- s.class(pca.filt$scores, gl.lonspi.filt@strata$species, xax=1, yax=2,

col=cols, ellipseSize=0, starSize=0, ppoints.cex=1.2, paxes.draw=T,

pgrid.draw = F, plab.cex = 0 , plot = FALSE)

g2 <- s.label (pca.filt$scores, xax=1, yax=2, ppoints.col = "red",

plabels = list(box = list(draw = FALSE), optim = T),

ppoints.cex=0.5, paxes.draw=T, pgrid.draw =F, plabels.cex=1, plot = F)

ADEgS(c(g1, g2), layout = c(1, 2))

# different colors for ploidy

g1 <- s.class(pca.filt$scores, as.factor(as.vector(gl.lonspi.filt@strata$ploidy)),

xax=1, yax=2, col=cols, ellipseSize=0, starSize=0, ppoints.cex=1.2,

paxes.draw=T, pgrid.draw = F, plab.cex = 0 , plot = FALSE)

ADEgS(c(g1, g2), layout = c(1, 2))

title("fast PCA")

# to view results of PCA we can use ggplot2

lonspi.pca1.scores <- as.data.frame(pca.filt$scores)

lonspi.pca1.scores$pop <- ploidy(gl.lonspi.filt)#add population values as new column in the object, so we can color samples by population

cols <- brewer.pal(n=nlevels(gl.lonspi.filt$strata$species), name="Dark2")

library(ggplot2)

set.seed(9)

ggplot(lonspi.pca1.scores, aes(x=PC1, y=PC2, colour=gl.lonspi.filt$strata$species)) +

geom_point(size=1) +

stat_ellipse(level=0.95, size=0.3) +

theme_bw()

# check here for guide on ggrepel https://github.com/slowkow/ggrepel/blob/master/vignettes/ggrepel.md#hide-some-of-the-labels

library(ggrepel)

ggplot(lonspi.pca1.scores, aes(x=PC1, y=PC2, label=gl.lonspi.filt$strata$id)) +

geom_text_repel() +

geom_point(color = cols[gl.lonspi.filt$strata$species]) +

theme_classic(base_size = 2)

# PCA known ploidy ####

source(file="custom_functions.R")

pca.filt.plo <-glPcaFast(gl.lonspi.filt.plo, center=F, # center means of measurements

nf=4) # function defined in file "custom_functions.R"

# proportion of explained variance by first three axes

ax1 = pca.filt.plo$eig[1]/sum(pca.filt.plo$eig) # proportion of variation explained by 1st axis

ax2 = pca.filt.plo$eig[2]/sum(pca.filt.plo$eig) # proportion of variation explained by 2nd axis

ax3 = pca.filt.plo$eig[3]/sum(pca.filt.plo$eig) # proportion of variation explained by 3rd axis

sum(ax1,ax2,ax3)

barplot(100*pca.filt.plo$eig/sum(pca.filt.plo$eig),

col=heat.colors(50),

main="fast PCA Eigenvalues on filtered \nonly known ploidy")

title(ylab="Percent of variance\nexplained", line=2)

title(xlab="Eigenvalues", line=1)

# plot different colors for species

# cols <- brewer.pal(n = nlevels(gl.lonspi.filt.plo$strata$species), #number if different colors in the palette

# name = "Dark2")

g1 <- s.class(pca.filt.plo$scores, gl.lonspi.filt.plo$strata$species, xax=1, yax=2,

col=cols, ellipseSize=0, starSize=0, ppoints.cex=0.8, paxes.draw=T,

pgrid.draw = F, plab.cex = 0 , plot = FALSE)

g2 <- s.label (pca.filt.plo$scores, xax=1, yax=2, ppoints.col = "red",

plabels = list(box = list(draw = FALSE), optim = T),

ppoints.cex=0.5, paxes.draw=T, pgrid.draw =F, plabels.cex=0.5, plot = F)

ADEgS(c(g1, g2), layout = c(1, 2))

# different colors for ploidy

g1 <- s.class(pca.filt.plo$scores, as.factor(as.vector(ploidy(gl.lonspi.filt.plo))),

xax=1, yax=2, col=cols, ellipseSize=0, starSize=0, ppoints.cex=0.5,

paxes.draw=T, pgrid.draw = F, plab.cex = 0 , plot = FALSE)

ADEgS(c(g1, g2), layout = c(1, 2))

# PCA diploids ####

source(file="custom_functions.R")

pca.filt.dip <-glPcaFast(gl.lonspi.filt.dip, center=F, # center means of measurements

nf=4) # function defined in file "custom_functions.R"

# proportion of explained variance by first three axes

ax1 = pca.filt.dip$eig[1]/sum(pca.filt.dip$eig) # proportion of variation explained by 1st axis

ax2 = pca.filt.dip$eig[2]/sum(pca.filt.dip$eig) # proportion of variation explained by 2nd axis

ax3 = pca.filt.dip$eig[3]/sum(pca.filt.dip$eig) # proportion of variation explained by 3rd axis

sum(ax1,ax2,ax3)

barplot(100*pca.filt.dip$eig/sum(pca.filt.dip$eig),

col=heat.colors(50),

main="fast PCA Eigenvalues on filtered \nonly diploids")

title(ylab="Percent of variance\nexplained", line=2)

title(xlab="Eigenvalues", line=1)

# cols <- brewer.pal(n = nlevels(gl.lonspi.filt.dip$strata$species), #number if different colors in the palette

# name = "Dark2")

g1 <- s.class(pca.filt.dip$scores, gl.lonspi.filt.dip$strata$species, xax=1, yax=2,

col=cols, ellipseSize=0, starSize=0, ppoints.cex=0.8, paxes.draw=T,

pgrid.draw = F, plab.cex = 0 , plot = FALSE)

g2 <- s.label (pca.filt.dip$scores, xax=1, yax=2, ppoints.col = "red",

plabels = list(box = list(draw = FALSE), optim = T),

ppoints.cex=0.5, paxes.draw=T, pgrid.draw =F, plabels.cex=0.5, plot = F)

ADEgS(c(g1, g2), layout = c(1, 2))

# PCA tetraploids ####

source(file="custom_functions.R")

pca.filt.tet <-glPcaFast(gl.lonspi.filt.tet, center=F, # center means of measurements

nf=4) # function defined in file "custom_functions.R"

# proportion of explained variance by first three axes

ax1 = pca.filt.tet$eig[1]/sum(pca.filt.tet$eig) # proportion of variation explained by 1st axis

ax2 = pca.filt.tet$eig[2]/sum(pca.filt.tet$eig) # proportion of variation explained by 2nd axis

ax3 = pca.filt.tet$eig[3]/sum(pca.filt.tet$eig) # proportion of variation explained by 3rd axis

sum(ax1,ax2,ax3)

barplot(100*pca.filt.tet$eig/sum(pca.filt.tet$eig),

col=heat.colors(50),

main="fast PCA Eigenvalues on filtered \nonly tetraploids")

title(ylab="Percent of variance\nexplained", line=2)

title(xlab="Eigenvalues", line=1)

# cols <- brewer.pal(n = nlevels(gl.lonspi.filt.tet$strata$species), #number if different colors in the palette

# name = "Dark2")

g1 <- s.class(pca.filt.tet$scores, gl.lonspi.filt.tet$strata$species, xax=1, yax=2,

col=cols, ellipseSize=0, starSize=0, ppoints.cex=0.8, paxes.draw=T,

pgrid.draw = F, plab.cex = 0 , plot = FALSE)

g2 <- s.label (pca.filt.tet$scores, xax=1, yax=2, ppoints.col = "red",

plabels = list(box = list(draw = FALSE), optim = T),

ppoints.cex=0.5, paxes.draw=T, pgrid.draw =F, plabels.cex=0.5, plot = F)

ADEgS(c(g1, g2), layout = c(1, 2))

# PCA spicata ####

source(file="custom_functions.R")

ploidy(gl.spi.filt)<-2

rm(gl.spi.filt)

load(file="gl.spi.filt.Rdata")

pca.filt.spi <-glPcaFast(gl.spi.filt, center=F, # center means of measurements

nf=4) # function defined in file "custom_functions.R"

# proportion of explained variance by first three axes

ax1 = pca.filt.spi$eig[1]/sum(pca.filt.spi$eig) # proportion of variation explained by 1st axis

ax2 = pca.filt.spi$eig[2]/sum(pca.filt.spi$eig) # proportion of variation explained by 2nd axis

ax3 = pca.filt.spi$eig[3]/sum(pca.filt.spi$eig) # proportion of variation explained by 3rd axis

sum(ax1,ax2,ax3)

barplot(100*pca.filt.spi$eig/sum(pca.filt.spi$eig),

col=heat.colors(50),

main="fast PCA Eigenvalues on filtered \nspicata")

title(ylab="Percent of variance\nexplained", line=2)

title(xlab="Eigenvalues", line=1)

# plot different colors for species

# cols <- brewer.pal(n = nlevels(gl.spi.filt$strata$species), #number if different colors in the palette

# name = "Dark2")

g1 <- s.class(pca.filt.spi$scores, gl.spi.filt$strata$species,

xax=1, yax=2,

col=cols, ellipseSize=0, starSize=0, ppoints.cex=1.2, paxes.draw=T,

pgrid.draw = F, plab.cex = 0 , plot = FALSE)

g2 <- s.label (pca.filt.spi$scores, xax=1, yax=2, ppoints.col = "red",

plabels = list(box = list(draw = FALSE), optim = T),

ppoints.cex=0.5, paxes.draw=T, pgrid.draw =F, plabels.cex=0.8, plot = F)

ADEgS(c(g1, g2), layout = c(1, 2))

# different colors for ploidy

g1 <- s.class(pca.filt.spi$scores, as.factor(as.vector(ploidy(gl.spi.filt))),

xax=1, yax=2, col=cols, ellipseSize=0, starSize=0, ppoints.cex=1.2,

paxes.draw=T, pgrid.draw = F, plab.cex = 0 , plot = FALSE)

ADEgS(c(g1, g2), layout = c(1, 2))

# PCA longifolia ####

source(file="custom_functions.R")

ploidy(gl.lon.filt)<-4

rm(gl.lon.filt)

load(file="gl.lon.filt.Rdata")

pca.filt.lon <-glPcaFast(gl.lon.filt, center=F, # center means of measurements

nf=4) # function defined in file "custom_functions.R"

# proportion of explained variance by first three axes

ax1 = pca.filt.lon$eig[1]/sum(pca.filt.lon$eig) # proportion of variation explained by 1st axis

ax2 = pca.filt.lon$eig[2]/sum(pca.filt.lon$eig) # proportion of variation explained by 2nd axis

ax3 = pca.filt.lon$eig[3]/sum(pca.filt.lon$eig) # proportion of variation explained by 3rd axis

sum(ax1,ax2,ax3)

barplot(100*pca.filt.lon$eig/sum(pca.filt.lon$eig),

col=heat.colors(50),

main="fast PCA Eigenvalues on filtered \nlongifolia")

title(ylab="Percent of variance\nexplained", line=2)

title(xlab="Eigenvalues", line=1)

# plot different colors for species

# cols <- brewer.pal(n = nlevels(gl.lon.filt$strata$species), #number if different colors in the palette

# name = "Dark2")

g1 <- s.class(pca.filt.lon$scores, gl.lon.filt$strata$species, xax=1, yax=2,

col=cols, ellipseSize=0, starSize=0, ppoints.cex=1.2, paxes.draw=T,

pgrid.draw = F, plab.cex = 0 , plot = FALSE)

g2 <- s.label (pca.filt.lon$scores, xax=1, yax=2, ppoints.col = "red",

plabels = list(box = list(draw = FALSE), optim = T),

ppoints.cex=0.5, paxes.draw=T, pgrid.draw =F, plabels.cex=0.8, plot = F)

ADEgS(c(g1, g2), layout = c(1, 2))

# different colors for ploidy

g1 <- s.class(pca.filt.lon$scores, as.factor(as.vector(ploidy(gl.lon.filt))),

xax=1, yax=2, col=cols, ellipseSize=0, starSize=0, ppoints.cex=1.2,

paxes.draw=T, pgrid.draw = F, plab.cex = 0 , plot = FALSE)

ADEgS(c(g1, g2), layout = c(1, 2))

# TREES

# upgma tree with problematic samples ####

tree.problematics <- aboot(gl.problematics, #genlight object

tree="upgma", #algorithm to use

distance=dist, #how calculate distance matrix (bitwise::dist is not working with different ploidy levels)

sample=100, #bootstrap replicates to assess branch support

showtree=F,

cutoff=5,

quiet=F)

plot.phylo(tree.problematics, cex = 0.7, font = 2, adj = 0,

tip.color = cols[gl.problematics@strata$species],

main="upgma tree with problematic V. spicata and V. longifolia samples")

nodelabels(tree.problematics$node.label, adj = c(1.3, -0.5), frame = "n",

cex = 0.7,font = 1, xpd = TRUE, col="blue")

axis(side = 1, lwd=1)

title(xlab = "Genetic distance", cex=0.5)

# upgma tree with only spicata and longifolia ####

load(file="tree.lonspi.only.Rdata")

tree.lonspi.only <- aboot(gl.lonspi.only, #genlight object

tree="upgma", #algorithm to use

distance=dist, #how calculate distance matrix (bitwise::dist is not working with different ploidy levels)

sample=100, #bootstrap replicates to assess branch support

showtree=F,

cutoff=5,

quiet=F)

save(tree.lonspi.only,file="tree.lonspi.only.Rdata")

#write.nexus(tree.ploidy.filt, file="distance_tree_ploidy_filt_1.tree")

#cols <- brewer.pal(n = nlevels(gl.lonspi.filt.plo$strata$species), #number if different colors in the palette

#

#name = "Dark2")

plot.phylo(tree.lonspi.only, cex = 0.7, font = 2, adj = 0,

tip.color = cols[gl.lonspi.only@strata$species],

main="upgma tree with only V. spicata and V. longifolia samples")

nodelabels(tree.lonspi.only$node.label, adj = c(1.3, -0.5), frame = "n",

cex = 0.7,font = 1, xpd = TRUE, col="blue")

# cols2<-brewer.pal(n = nlevels(as.factor(gl.lonspi.filt.plo@strata$area)),

# name = "Paired")[gl.lonspi.filt.plo@strata$area]

# tiplabels(gl.lonspi.filt.plo$strata$area, frame = "n", cex=0.4, offset = 10,

# col=cols2, adj=c(1,0))

legend('topleft', legend = levels(gl.lonspi.filt.plo@strata$species),

fill = cols, border = FALSE, bty = "n", cex = 0.8)

axis(side = 1, lwd=1)

title(xlab = "Genetic distance", cex=0.5)

# upgma tree with all ploidy levels ####

load(file="tree.ploidy.filt.Rdata")

tree.ploidy.filt <- aboot(gl.lonspi.filt.plo, #genlight object

tree="upgma", #algorithm to use

distance=dist, #how calculate distance matrix (bitwise::dist is not working with different ploidy levels)

sample=100, #bootstrap replicates to assess branch support

showtree=F,

cutoff=5,

quiet=F)

save(tree.ploidy.filt,file="tree.ploidy.filt.Rdata")

#write.nexus(tree.ploidy.filt, file="distance_tree_ploidy_filt_1.tree")

#cols <- brewer.pal(n = nlevels(gl.lonspi.filt.plo$strata$species), #number if different colors in the palette

#

#name = "Dark2")

plot.phylo(tree.ploidy.filt, cex = 0.7, font = 2, adj = 0,

tip.color = cols[gl.lonspi.filt.plo@strata$species],

main="Phylogenetic tree with filtered samples of known ploidy level")

nodelabels(tree.ploidy.filt$node.label, adj = c(1.3, -0.5), frame = "n",

cex = 0.7,font = 1, xpd = TRUE, col="blue")

# cols2<-brewer.pal(n = nlevels(as.factor(gl.lonspi.filt.plo@strata$area)),

# name = "Paired")[gl.lonspi.filt.plo@strata$area]

# tiplabels(gl.lonspi.filt.plo$strata$area, frame = "n", cex=0.4, offset = 10,

# col=cols2, adj=c(1,0))

legend('topleft', legend = levels(gl.lonspi.filt.plo@strata$species),

fill = cols, border = FALSE, bty = "n", cex = 0.8)

axis(side = 1, lwd=1)

title(xlab = "Genetic distance", cex=0.5)

# tree with all filtered samples ####

load(file="tree.all.filt.RData")

tree.all.filt<- aboot(gl.lonspi.filt, #genlight object

tree="upgma", #algorithm to use

distance=dist, #how calculate distance matrix (bitwise::dist is not working with different ploidy levels)

sample=100, #bootstrap replicates to assess branch support

showtree=F,

cutoff=20,

quiet=F)

#write.nexus(tree.all.filt, file="distance_tree_all_filt_1.tree")

write.nexus(tree.all.filt, file="upgma_lonspischxm_50miss_whitelist.tree")

save(tree.all.filt,file="tree.all.filt.Rdata")

#cols <- brewer.pal(n = nlevels(gl.lonspi.filt$strata$species), #number if different colors in the palette

# name = "Dark2")

plot.phylo(tree.all.filt, cex = 0.8, font = 2, adj = 0,

tip.color = cols[gl.lonspi.filt$strata$species],

main="upgma tree with filtered samples")

plot.phylo(tree.all.filt, cex = 0.8, font = 2, adj = 0,

type="phylogram",

#use.edge.length = T,

#node.depth = 2,

tip.color = cols[gl.lonspi.filt$strata$species],

main="upgma tree with filtered samples")

nodelabels(tree.all.filt$node.label, adj = c(1.3, -0.5), frame = "n",

cex = 0.7,font = 1, xpd = TRUE, col="blue")

#cols2<-brewer.pal(n = nlevels(as.factor(gl.lonspi.filt@strata$area)),

# name = "Paired")[gl.lonspi.filt@strata$area]

#tiplabels(gl.lonspi.filt$strata$area, frame = "n", cex=0.4, offset = 10,

# col=cols2, adj=c(1,0))

legend('topleft', legend = levels(gl.lonspi.filt$strata$species),

fill = cols, border = FALSE, bty = "n", cex = 0.8)

axis(side = 1, lwd=1,cex=0.8)

title(xlab = "Genetic distance", cex=0.8)

# tree with all filtered samples including only spicata, longifolia and schmidtiana ####

gl.lonspi.filt2<-gl.lonspi.filt[gl.lonspi.filt$strata$species=="longifolia" |

gl.lonspi.filt$strata$species=="spicata" |

gl.lonspi.filt$strata$species=="schmidtiana"]

save(gl.lonspi.filt2,file="gl.lonspisch.only.Rdata")

load(file="tree.all.filt.RData")

tree.lonspisch<- aboot(gl.lonspi.filt2, #genlight object

tree="upgma", #algorithm to use

distance=dist, #how calculate distance matrix (bitwise::dist is not working with different ploidy levels)

sample=100, #bootstrap replicates to assess branch support

showtree=F,

cutoff=10,

quiet=F)

save(tree.lonspisch,file="tree.lonspisch.Rdata")

write.nexus(tree.lonspisch, file="upgma_lonspisch_50miss.tree")

#cols <- brewer.pal(n = nlevels(gl.lonspi.filt$strata$species), #number if different colors in the palette

# name = "Dark2")

plot.phylo(tree.lonspisch, cex = 0.8, font = 2, adj = 0,

tip.color = cols[gl.lonspi.filt2$strata$species],

main="upgma tree 50% missing data")

nodelabels(tree.all.filt$node.label, adj = c(1.0, -0.2), frame = "n",

cex = 0.6,font = 1, xpd = TRUE, col="blue")

legend('topleft', legend = levels(gl.lonspi.filt2$strata$species),

fill = cols, border = FALSE, bty = "n", cex = 0.8)

axis(side = 1, lwd=1,cex=0.8)

title(xlab = "Genetic distance", cex=0.8)

# tree with all spicata and longifolia ####

gl.lonspi.filt3<-gl.lonspi.filt[gl.lonspi.filt$strata$species=="spicata" | gl.lonspi.filt$strata$species=="longifolia"]

load(file="tree.all.filt.RData")

tree.all.filt.3 <- aboot(gl.lonspi.filt3, #genlight object

tree="upgma", #algorithm to use

distance=dist, #how calculate distance matrix (bitwise::dist is not working with different ploidy levels)

sample=100, #bootstrap replicates to assess branch support

showtree=F,

cutoff=10,

quiet=F)

#write.nexus(tree.all.filt, file="distance_tree_all_filt_1.tree")

save(tree.all.filt,file="tree.lonspi_only.Rdata")

#cols <- brewer.pal(n = nlevels(gl.lonspi.filt$strata$species), #number if different colors in the palette

# name = "Dark2")

plot.phylo(tree.all.filt.3, cex = 0.8, font = 2, adj = 0,

tip.color = cols[gl.lonspi.filt3$strata$species],

main="Phylogenetic tree with spicata and longifolia only")

nodelabels(tree.all.filt.3$node.label, adj = c(1.3, -0.5), frame = "n",

cex = 0.7,font = 1, xpd = TRUE, col="blue")

legend('topleft', legend = levels(gl.lonspi.filt2$strata$species),

fill = cols, border = FALSE, bty = "n", cex = 0.8)

axis(side = 1, lwd=1,cex=0.8)

title(xlab = "Genetic distance", cex=0.8)

# tree only diploids ####

#tree.dip.filt <-read.nexus("distance_tree_dip_filt_1.tree")

tree.dip.filt<- aboot(gl.lonspi.filt.dip, #genlight object

tree="upgma", #algorithm to use

distance=dist, #def: nei.dist how calculate distance matrix (bitwise::dist is not working with different ploidy levels)

sample=100, #bootstrap replicates to assess branch support

showtree=F,

cutoff=10,

quiet=F)

# try with other options

tree.dip.filt<- aboot(gl.lonspi.filt.dip, #genlight object

tree="upgma", #algorithm to use

distance=bitwise.dist, #how calculate distance matrix, default nei.dist

sample=100, #bootstrap replicates to assess branch support

showtree=F,

cutoff=10)

save(tree.dip.filt,file="tree_dip_filt.Rdata")

#write.nexus(tree.dip.filt, file="distance_tree_dip_filt_1.tree")

#cols <- brewer.pal(n = nlevels(gl.lonspi.filt.dip$strata$species), #number if different colors in the palette

# name = "Dark2")

plot.phylo(tree.dip.filt, cex = 0.8, font = 2, adj = 0,

tip.color = cols[gl.lonspi.filt.dip$strata$species],

main="Phylogenetic tree with filtered samples\ndiploids only")

nodelabels(tree.dip.filt$node.label, adj = c(1.3, -0.5), frame = "n",

cex = 0.6,font = 1, xpd = TRUE, col="blue")

#cols2<-brewer.pal(n = nlevels(as.factor(gl.lonspi.filt.dip@strata$area)),

# name = "Paired")[gl.lonspi.filt.dip@strata$area]

#tiplabels(gl.lonspi.filt.dip$strata$area, frame = "n", cex=0.8, offset = 15,

# col=cols2, adj=c(1,0))

legend('topleft', legend = levels(gl.lonspi.filt.dip$strata$species),

fill = cols, border = FALSE, bty = "n", cex = 0.8)

axis(side = 1, lwd=1)

title(xlab = "Genetic distance", cex=0.5)

# tree only tetraploids ####

#tree.tet.filt <-read.nexus("distance_tree_tet_filt_1.tree")

tree.tet.filt<- aboot(gl.lonspi.filt.tet, #genlight object

tree="upgma", #algorithm to use

distance=dist, #how calculate distance matrix (bitwise::dist is not working with different ploidy levels)

sample=100, #bootstrap replicates to assess branch support

showtree=F,

cutoff=10,

quiet=F)

save(tree.tet.filt,file="tree.tet.filt.Rdata")

#write.nexus(tree.tet.filt, file="distance_tree_tet_filt_1.tree")

#cols <- brewer.pal(n = nlevels(gl.lonspi.filt.tet$strata$species), #number if different colors in the palette

# name = "Dark2")

plot.phylo(tree.tet.filt, cex = 0.8, font = 2, adj = 0,

tip.color = cols[gl.lonspi.filt.tet$strata$species],

main="Phylogenetic tree with filtered samples\ntetraploids only")

nodelabels(tree.tet.filt$node.label, adj = c(1.3, -0.5), frame = "n",

cex = 0.8,font = 1, xpd = TRUE, col="blue")

#cols2<-brewer.pal(n = nlevels(as.factor(gl.lonspi.filt.tet@strata$area)),

# name = "Paired")[gl.lonspi.filt.tet@strata$area]

tiplabels(gl.lonspi.filt.tet$strata$id_sample, frame = "n", cex=0.6, offset = 10,

col=cols2, adj=c(1,0))

legend('topleft', legend = levels(gl.lonspi.filt.tet$strata$species),

fill = cols, border = FALSE, bty = "n", cex = 0.8)

axis(side = 1, lwd=1)

title(xlab = "Genetic distance", cex=0.5)

# tree obtained with vk tool ####

library(treeio)

setwd("ML tree/")

tree.vk <-read.newick("RAxML_bestTree.T1")

library(ape)

plot.phylo(tree.vk, cex = 0.4, font = 2, adj = 0,

#tip.color = cols[sample.data$species],

main="ML phylogenetic tree with all samples")

nodelabels(tree.vk$node.label, adj = c(1.3, -0.5), frame = "n",

cex = 0.4,font = 1, xpd = TRUE, col="blue")

legend('topleft', legend = levels(sample.data$species),

fill = cols, border = FALSE, bty = "n", cex = 0.4)

axis(side = 1, cex=0.4)

title(xlab = "Genetic distance")

ggtree(tree.vk,

#branch.length = "none" #turn tree into a cladogram

#layout="circular",

mrsd='2018-01-01') + theme_tree2() + ggtitle("Divergence time")

# check here for ggtree: https://www.molecularecologist.com/2017/02/phylogenetic-trees-in-r-using-ggtree/

# and here: https://bioconductor.org/packages/release/bioc/vignettes/ggtree/inst/doc/treeManipulation.html

library("ggtree")

tree.ploidy <-read.tree("distance_tree_ploidy.tree")

ggtree(tree.ploidy,

#branch.length = "none" #turn tree into a cladogram

#layout="circular",

mrsd='2018-01-01') + theme_tree2() + ggtitle("Divergence time")

# + geom_treescale()

t <- groupClade(tree.ploidy,node=c(37,58))

ggtree(t, aes(color=group, linetype=group)) + geom_tiplab() + geom_nodelab(aes(x=branch), vjust=-.5, size=3)

ggtree(tree.ploidy)+geom_text2(aes(subset=!isTip, label=node), hjust=-.3) + geom_tiplab()

# Minimum spanning network ####

library(igraph)

#msn diploid

lonspi.dist.dip <- dist(gl.lonspi.filt.dip) #matrix of genetic distance between samples (bitwise.dist is not working with different ploidy levels)

lonspi.msn.dip <- poppr.msn(gl.lonspi.filt.dip, #genlight object

lonspi.dist.dip, # matrix of genetic distances

showplot = F, include.ties = T) # msn=minimun spanning network

node.size <- rep(1, times = nInd(gl.lonspi.filt.dip))

names(node.size) <- indNames(gl.lonspi.filt.dip)

vertex.attributes(lonspi.msn.dip$graph)$size <- node.size

cols2<-brewer.pal(n = 5, name = "Dark2")

set.seed(2) # set a random number seed, changing it we obtain different representations of the same data

plot_poppr_msn(gl.lonspi.filt.dip, # genlight object

lonspi.msn.dip, # minimum spanning network of data

palette=cols2,

#col=cols,

gadj=100,

wscale=F,#edge widths scaled proportionally to inverse of observed distance (thicker = more related nodes)

scale.leg=T)

#msn tetraploids NOT WORKING WITH PLOIDY = 4

# try to tricky it assigning ploidy = 2

gl.tet2<-gl.lonspi.filt.tet

ploidy(gl.tet2)<-2

lonspi.dist.tet <- dist(gl.tet2) #matrix of genetic distance between samples (bitwise.dist is not working with different ploidy levels)

lonspi.msn.tet <- poppr.msn(gl.tet2, #genlight object

lonspi.dist.tet, # matrix of genetic distances

showplot = F, include.ties = T) # msn=minimun spanning network

node.size <- rep(1, times = nInd(gl.tet2))

names(node.size) <- indNames(gl.tet2)

vertex.attributes(lonspi.msn.tet$graph)$size <- node.size

cols2<-brewer.pal(n = 6, name = "Dark2")

set.seed(1) # set a random number seed, changing it we obtain different representations of the same data

plot_poppr_msn(gl.tet2, # enlight object

lonspi.msn.tet, # minimum spanning network of data

palette=cols2,

gadj=100,

wscale=F,#edge widths scaled proportionally to inverse of observed distance (thicker = more related nodes)

scale.leg=T)

# neighbourNet Network all ind ####

library(phangorn)

load(file="gl.lonspischxm.Rdata")

# subset to reduce number of individuals

id_retain<-c("lo54t_Slo4","lo43t_Slo3","lo57t_Slo4","lo36t_Slo2","lo21t_Hun5","lo26t_Hun8", #longifolia

"lo14d_Hun2","lo30t_Hun8","lo75d_Ukr13","lo67t_Ukr12","lo49x_Fra11","lo2x_Fra1",

"lo95d_Fin2","lo94d_Fin1","lo66x_Ger4","lo62x_Ger2","lo19x_Aus3","lo96t_Rus18",

"lo83t_Ukr17","lo84t_Ukr18","xm1t_Ukr20","sp73t_Ukr19","lo37t_Slo2","lo15t_Ukr2",#"sp7d_Swi2", #hybrids

"sp39t_Aus5","sp49t_Aus6","sp57t_Slo1","sp56t_Slo1","fi5t_Hun9","sp27t_Hun6", #spicata

"sp19d_Fra4", "sp18d_Fra4","sp5d_Swi1","sp83t_UK2","sp3t_Ser21","sp84t_Pol3","sp76t_Pol1",

"sp65x_Ger8","sp86d_Rus17","sp16t_Rus3","sp17t_Rus3","fi1t_Rom1","sp9t_Rom2",

"sc2d_Jap1")

gl.subs<-gl.lonspischxm[gl.lonspischxm@strata$new_id%in%id_retain]

gl.subs@strata$new_id<-as.character(gl.subs@strata$new_id)

gl.subs@strata$new_id<-as.factor(gl.subs@strata$new_id)

gl.subs@strata$new_id

dm<-dist(gl.subs)

#dm <-dist(gl.lonspi.filt)

# export distance matrix for splitstree

# library(reshape2)

# dm2<-as.matrix(dm)

# write.csv(dm2, file="distanceMatrix_lonspischxm_50miss.csv")

nnet.lonspi <- neighborNet(dm)

save(nnet.lonspi, file="nnet.lonspi.Rdata")

#cols <- brewer.pal(n = nlevels(gl.lonspi.filt.dip$strata$species), #number if different colors in the palette

# name = "Dark2")

col_tips=NULL

cols <- c("#D95F02","#7570B3","#1B9E77","#E7298A")

n<-0

for (i in nnet.lonspi$tip.label){

col_tips=c(col_tips,cols[gl.subs@strata$species[gl.subs$ind.names==i]])

n=n+1

}

n

col_tips

par("mar" = rep(2, 4))

plot(nnet.lonspi, cex=0.6, edge.width=0.5,

tip.color = col_tips,

set.seed(19))

title(main = "NeighbourNet Network using only singletons")

legend('bottomleft', legend = levels(gl.lonspi.filt$strata$species),

fill = cols, border = FALSE, bty = "n", cex = 0.6)

plot(nnet.lonspi,"2D",tip.color = col_tips,cex=0.6, edge.width=0.5, show.edge.label=F)

#nnet lonspi without possible hybrids ####

library(phangorn)

load(file="gl.lonspi.only.noHyb.Rdata")

dm.noHyb <-dist(gl.lonspi.only.noHyb)

nnet.lonspi.noHyb <- neighborNet(dm.noHyb)

save(nnet.lonspi.noHyb, file="nnet.lonspi.noHyb.Rdata")

#cols <- brewer.pal(n = nlevels(gl.lonspi.filt.dip$strata$species), #number if different colors in the palette

# name = "Dark2")

col_tips=NULL

n<-0

for (i in nnet.lonspi.noHyb$tip.label){

col_tips=c(col_tips,cols[gl.lonspi.only.noHyb@strata$species[gl.lonspi.only.noHyb$ind.names==i]])

n=n+1

}

n

col_tips

#par("mar" = rep(2, 4))

par(mfrow=c(1,1)) # Setting plot parameters

plot(nnet.lonspi.noHyb,"2D",tip.color = col_tips,cex=0.6, edge.width=0.5, show.edge.label=F)

plot(nnet.lonspi.noHyb, cex=0.6, edge.width=0.5,

tip.color = col_tips,

set.seed(4))

title(main = "NeighbourNet Network diploids")

legend('bottomleft', legend = levels(gl.lonspi.filt.dip$strata$species),

fill = cols, border = FALSE, bty = "n", cex = 0.6)

# neighbourNet Network diploids ####

#see https://cran.r-project.org/web/packages/phangorn/vignettes/IntertwiningTreesAndNetworks.html

library(phangorn)

#lonspi.phyDat<-read.phyDat("~/thesis SNPs data/partial_dataset/longifolia-spicata_daniele18.u.snps.phy")

#lonspi.diploid.phyDat<-genlight2phyDat(gl.lonspi.filt.dip)

#saveRDS(lonspi.diploid.phyDat,"lonspi_filt_dip.phy")

#summary(lonspi.diploid.phyDat)

#dm <- dist.hamming(lonspi.diploid.phyDat) #try with uncorrected p.distance

dm <-dist(gl.lonspi.filt.dip)

nnet.dip <- neighborNet(dm)

#cols <- brewer.pal(n = nlevels(gl.lonspi.filt.dip$strata$species), #number if different colors in the palette

# name = "Dark2")

col_tips=NULL

n<-0

for (i in nnet.dip$tip.label){

col_tips=c(col_tips,cols[gl.lonspi.filt.dip@strata$species[gl.lonspi.filt.dip$ind.names==i]])

n=n+1

}

n

col_tips

par("mar" = rep(2, 4))

plot(nnet.dip, cex=0.6, edge.width=0.5,

tip.color = col_tips,

set.seed(19))

title(main = "NeighbourNet Network diploids")

legend('bottomleft', legend = levels(gl.lonspi.filt.dip$strata$species),

fill = cols, border = FALSE, bty = "n", cex = 0.6)

# neighbourNet Network tetraploids ####

lonspi.tet.phyDat<-genlight2phyDat(gl.lonspi.filt.tet)

saveRDS(lonspi.diploid.phyDat,"lonspi_filt_tet.phy")

summary(lonspi.tet.phyDat)

nnet.tet <- read.nexus.networx("nnet_filt_1_tet.nxs")

dm <- dist(gl.lonspi.filt.tet)

nnet.tet <- neighborNet(dm)

#write.nexus.networx(nnet.tet,"nnet_filt_1_tet.nxs")

#cols <- brewer.pal(n = nlevels(gl.lonspi.filt.tet$strata$species), #number of different colors in the palette

# name = "Dark2")

col_tips=NULL

for (i in nnet.tet$tip.label){

col_tips=c(col_tips,cols[gl.lonspi.filt.tet@strata$species[gl.lonspi.filt.tet$ind.names==i]])

}

col_tips

par("mar" = rep(2, 4))

plot(nnet.tet, cex=0.6, edge.width=0.5,

tip.color = col_tips, set.seed(16))

title(main = "NeighbourNet Network filt 4 tetraploids")

legend('bottomleft', legend = levels(gl.lonspi.filt.dip$strata$species),

fill = cols, border = FALSE, bty = "n", cex = 0.6)

# see https://researchinpeace.blogspot.com/2018/02/how-did-i-do-it-short-guide-to-nice.html

lonspi.phyDat

phy.lonspi.noHyb <-genlight2phyDat(gl.lonspi.only.noHyb)

save(phy.lonspi.noHyb,file="phy.lonspi.noHyb.RData")

dm <- dist.hamming(pd.lonspi.ploidy)

nnet <- neighborNet(dm)

par("mar" = rep(1, 4))

plot(nnet, "2D")

library(ips)

db.lonspi<-vcfR2DNAbin(lonspi.VCF)

# following command does not work >:(

raxml.tree <- raxml(db.lonspi, f = "d", N = 2, p = 1234,

exec = "/home/daniele/R/x86_64-pc-linux-gnu-library/3.4/phangorn/extdata/trees/"

)

#load tree obtained with terminal command: ./raxml-ng --msa longifolia-spicata_daniele18.u.snps.phy --model GTR+G

# model used: GTR+G

raxml.tree <- read.tree("/home/daniele/Software/raxml-ng_v0.8.1_linux_x86_64/longifolia-spicata_daniele18.u.snps.phy.raxml.bestTree")

plot(raxml.tree, cex=.6,tip.color = cols[sample.data$species])

legend('topleft', legend = levels(sample.data$species),

fill = cols, border = FALSE, bty = "n", cex = 0.6)

nodelabels(raxml.tree$node.label, frame="none")

sample.data <- read.table("sample_data.txt", sep="\t", header=T)

sample.data

cols <- brewer.pal(n = nlevels(sample.data$species), #number of different colors in the palette

name = "Dark2") # palette name, for list: brewer.pal.info

plot.phylo(raxml.tree,"u", cex = 0.6, font = 2, adj = 0, tip.color = cols[sample.data$species],

main="RAxML tree")

nodelabels(raxml.tree$node.label, adj = c(1.3, -0.5), frame = "n", cex = 0.6,font = 1, xpd = TRUE)

legend('topleft', legend = levels(sample.data$species),

fill = cols, border = FALSE, bty = "n", cex = 0.6)

# create a vector of labels for the network corresponding to edges in the tree

edge.lab <- createLabel(nnet, raxml.tree, raxml.tree$edge[,2], "edge")

par(mfrow=c(1,1))

x<-plot(raxml.tree,"u", cex=.4)

edgelabels(raxml.tree$edge[,2],col="blue", frame="none", cex=.4)

# find edges that are in the network but not in the tree

edge.col <- createLabel(nnet, raxml.tree, "black", nomatch="red")

x <- plot(nnet, edge.label = edge.lab, show.edge.label = T, "2D", edge.color = edge.col,

col.edge.label = "blue", cex=.4)

x <- addConfidences(nnet,raxml.tree, scaler = .01) #get an error, probably because we don't have bootstrap support calculated in the tree??

# treemix ####

# 1) removing non biallelic alleles with vcftools, command:

# vcftools --gzvcf lonspi_filtered_4.vcf.gz --min-alleles 2 --max-alleles 2 --out lonspi_filt_4_biallelic.vcf --recode

# 2) conversion VCF to treemix done with stacks, command:

# /home/daniele/Software/stacks/bin/populations --in_vcf lonspi_filt_4_biallelic.vcf --treemix -O ./ -M pop_area.txt

# pop_map.txt is in the format:

# id <tab> area

# or id<tab>species

# or id<tab>country

source("/home/daniele/softwares/treemix-1.13/src/plotting_funcs.R")

plot_tree("out_tree_mig_2")

treemix.in <- read.table("lonspi_ren_filt_1_biallelic.p.treemix", sep=" ", header=T)

treemix.in2<-treemix.in

list=NULL

for (i in colnames(treemix.in2)){

treemix.in2<-treemix.in2[treemix.in2[i] != "0,0",]

list=c(list,i)

}

# treemix.in2<-treemix.in2[treemix.in2$Hungary != "0,0",]

# treemix.in2<-treemix.in2[treemix.in2$Slovakia != "0,0",]

# treemix.in2<-treemix.in2[treemix.in2$cult != "0,0",]

# treemix.in2<-treemix.in2[treemix.in2$Austria != "0,0",]

# treemix.in2<-treemix.in2[treemix.in2$Russia != "0,0",]

# treemix.in2<-treemix.in2[treemix.in2$Ukraine != "0,0",]

# treemix.in2<-treemix.in2[treemix.in2$RussiaRostov != "0,0",]

# treemix.in2<-treemix.in2[treemix.in2$France != "0,0",]

# treemix.in2<-treemix.in2[treemix.in2$Poland != "0,0",]

# treemix.in2<-treemix.in2[treemix.in2$RussiaUral != "0,0",]

#

# treemix.in2<-treemix.in2[treemix.in2$Mongolia != "0,0",]

# treemix.in2<-treemix.in2[treemix.in2$Romania != "0,0",]

# treemix.in2<-treemix.in2[treemix.in2$Finland != "0,0",]

# treemix.in2<-treemix.in2[treemix.in2$Germany != "0,0",]

# treemix.in2<-treemix.in2[treemix.in2$UnitedKingdom != "0,0",]

# treemix.in2<-treemix.in2[treemix.in2$Switzerland != "0,0",]

# treemix.in2<-treemix.in2[treemix.in2$RussiaWSiberia != "0,0",]

list

treemix.in2

write.table(treemix.in2,"lonspi_filt_bial_nonzero.treemix",quote = F, row.names = F)

plot_tree("out_stem")

treemix.in <- read.table("lonspi_biallelic.p.treemix", sep=" ", header=T)

treemix.in2<-treemix.in

list=NULL

for (i in colnames(treemix.in2)){

treemix.in2<-treemix.in2[treemix.in2[i] != "0,0",]

list=c(list,i)

}

plot_tree("out_stem")
