# Supplementary material for: Comparative Phylogeography of Veronica spicata and V. longifolia (Plantaginaceae) Across Europe: Integrating Hybridization and Polyploidy in Phylogeography
Source: Front Plant Sci. 2021 Feb 1;11:588354. doi: 10.3389/fpls.2020.588354 (PMC7884905; doi:10.3389/fpls.2020.588354)
Supplement: Supplementary file 7 [file Table_2.docx]

**TABLE S2 |** List of specimens used for ecological niche modelling with voucher (abbreviations according to Index Herbariorum), ploidy, country, holoploid genome size as 1C-value, coefficient of variation (CV), GPS-coordinates and reference. ^1^ *V. spicata* ssp*. euxina*, ^2^ *V. spicata* ssp*. fischeri*, ^3^ *V. spicata* ssp*. kamelinii*, ^4^ *V. spicata* ssp*. lanisepala*, ^5^ *V. spicata* var*. paczoskiana*, ^6^ *V. spicata* var*. pseudorchidea*.

| **Species** | **Vouchers** | **Ploidy** | **Country** | **1C-value** | **CV** | **Latitude (N-S)** | **Longitude (E-W)** | **References** |
| --- | --- | --- | --- | --- | --- | --- | --- | --- |
| *V. longifolia* | Fischer 2.8.1962, W | 2x | Austria |  |  | 48.0118 | 16.4549 | Fischer, 1969 |
| *V. longifolia* | Fischer 19.7.1965, W | 2x | Austria |  |  | 48.0140 | 16.4491 | Fischer, 1969 |
| *V. longifolia* | Fischer 4.8.1963, W | 2x | Austria |  |  | 48.2667 | 16.9284 | Fischer, 1969 |
| *V. longifolia* | Dzhus 1699a, MSKU | 2x | Byelorussia |  |  | 52.5686 | 23.7828 | Dzhus and Dmitrieva, 2001 |
| *V. longifolia* | Dzhus 1905, MSKU | 2x | Byelorussia |  |  | 53.8119 | 27.7224 | Dzhus and Dmitrieva, 2001 |
| *V. longifolia* | Stech 1995, OL | 2x | Czech Rep. |  |  | 48.8217 | 13.9707 | Travnicek, 2004 |
| *V. longifolia* | Travnicek et al., 1996, OL | 2x | Czech Rep. |  |  | 48.8217 | 13.9707 | Travnicek, 2004 |
| *V. longifolia* | Travnicek et al., 1996, OL | 2x | Czech Rep. |  |  | 48.8726 | 13.8901 | Travnicek, 2004 |
| *V. longifolia* | Albach S849, OLD | 2x | Finland | **0.7351** | 3.10-6.50 | 59.9688 | 24.3932 | new result |
| *V. longifolia* | Albach S848, OLD | 2x | Finland | **0.7293** | 2.90-5.20 | 59.9790 | 24.3931 | new result |
| *V. longifolia* | ?, H | 2x | Finland |  |  | 60.2300 | 25.1400 | Raitanen, 1967 |
| *V. longifolia* | Kukkonen 12856, H | 2x | Finland |  |  | 60.2683 | 22.2819 | Kukkonen, 1986 |
| *V. longifolia* | TUR 170916 | 2x | Finland |  |  | 60.3524 | 25.1260 | Uotila and Pellinen, 1985 |
| *V. longifolia* | Kukkonen 12869, H | 2x | Finland |  |  | 60.4236 | 20.2574 | Kukkonen, 1986 |
| *V. longifolia* | Kukkonen12866, H | 2x | Finland |  |  | 60.4236 | 20.2574 | Kukkonen, 1986 |
| *V. longifolia* | Kukkonen 12853, H | 2x | Finland |  |  | 60.4600 | 22.7040 | Kukkonen, 1986 |
| *V. longifolia* | ?, H | 2x | Finland |  |  | 60.4613 | 26.8964 | Raitanen, 1967 |
| *V. longifolia* | ?, H | 2x | Finland |  |  | 60.6820 | 22.6914 | Raitanen, 1967 |
| *V. longifolia* | ?, H | 2x | Finland |  |  | 60.7100 | 25.2200 | Raitanen, 1967 |
| *V. longifolia* | ?, H | 2x | Finland |  |  | 60.9492 | 22.2704 | Raitanen, 1967 |
| *V. longifolia* | Kukkonen s.n., H | 2x | Finland |  |  | 61.2167 | 26.0152 | Kukkonen, 1986 |
| *V. longifolia* | ?, H | 2x | Finland |  |  | 62.5350 | 23.6230 | Raitanen, 1967 |
| *V. longifolia* | ?, H | 2x | Finland |  |  | 62.6071 | 29.8069 | Raitanen, 1967 |
| *V. longifolia* | ?, H | 2x | Finland |  |  | 62.9427 | 22.5167 | Raitanen, 1967 |
| *V. longifolia* | ?, H | 2x | Finland |  |  | 63.0700 | 21.5000 | Raitanen, 1967 |
| *V. longifolia* | Kukkonen12910, H | 2x | Finland |  |  | 65.9685 | 29.2057 | Kukkonen, 1986 |
| *V. longifolia* | Kukkonen 12914, H | 2x | Finland |  |  | 67.1335 | 27.4889 | Kukkonen, 1986 |
| *V. longifolia* | ?, H | 2x | Finland |  |  | 69.7300 | 27.0000 | Raitanen, 1967 |
| *V. longifolia* | Albach 1547, OLD | 2x | Hungary | **0.7324** | 5.04 | 46.7993 | 16.3868 | new result |
| *V. longifolia* | Albach 1549, OLD | 2x | Hungary | **0.7118** | 7.51 | 47.1467 | 16.9092 | new result |
| *V. longifolia* | Härle U19, U18, TUB | 2x | Hungary |  |  | 47.2899 | 19.2028 | Graze, 1933 |
| *V. longifolia* | Härle "ticinensis", TUB | 2x | Italy |  |  | 45.1800 | 9.1300 | Graze, 1933 |
| *V. longifolia* | ? | 2x | Norway |  |  | 68.9900 | 23.0600 | Laane, 1965 |
| *V. longifolia* | Schöngart, Kosachev 9, OLD | 2x | Russia | **0.8957** | 12.52 | 49.5077 | 88.1660 | new result |
| *V. longifolia* | Pfanzelt et al., 1183.1, OLD | 2x | Russia | **0.8786** | 6.55 | 50.1569 | 88.2954 | new result |
| *V. longifolia* | Albach S625, OLD | 2x | Russia | **0.8153** | 3.14-3.78 | 50.4075 | 87.5971 | new result |
| *V. longifolia* | ? | 2x | Russia |  |  | 50.6800 | 95.2700 | Krasnikova et al., 1983 |
| *V. longifolia* | Prelovskaya 11845, VLA | 2x | Russia |  |  | 54.7823 | 105.2188 | Probatova et al., In Marhold, 2012 |
| *V. longifolia* | Stepanov 11.8.2015, KRSU | 2x | Russia |  |  | 55.9910 | 92.8580 | Stepanov, 2018 |
| *V. longifolia* | Lavrenko, Serditov, Ulle, SYCO | 2x | Russia |  |  | 62.7498 | 58.8365 | Lavrenko et al., 1991 |
| *V. longifolia* | M. Lomonosova 1144, NS | 2x | Russia |  |  | 67.4500 | 86.5317 | Ankova et al., In Marhold and Breitwieser, 2016 |
| *V. longifolia* | A. P. Sokolovskaja 20, VLA | 2x | Russia |  |  | 67.5100 | 65.0700 | Sokolovskaya, 1970 |
| *V. longifolia* | ? | 2x | Russia |  |  | 69.4400 | 88.3600 | Krogulevich ,1976 |
| *V. longifolia* | Travnicke 1993, OL | 2x | Slovakia |  |  | 48.2316 | 19.5973 | Travnicek, 2004 |
| *V. longifolia* | Travnicek 1993, OL | 2x | Slovakia |  |  | 48.2742 | 19.6550 | Travnicek, 2004 |
| *V. longifolia* | Lökvist D135, LD | 2x | Sweden |  |  | 39.0700 | 18.0700 | Lökvist and Hultgard, 1999 |
| *V. longifolia* | Lökvist 1323, LD | 2x | Sweden |  |  | 60.2600 | 18.3700 | Lökvist and Hultgard, 1999 |
| *V. longifolia* | Behcet 7435, OLD | 2x | Turkey | **0.9547** | 2.49-3.24 | 38.7660 | 40.9000 | Kosachev et al., 2016 |
| *V. longifolia* | Höpke 320, OLD | 2x | Ukraine | **0.7422** | 7.66 | 46.5974 | 32.5743 | new result |
| *V. longifolia* | Peev 17.2., SOM | 4x | Bulgaria |  |  | 43.7800 | 26.4800 | Löve, 1972 |
| *V. longifolia* | Dzhus 2014, MSKU | 4x | Byelorussia |  |  | 51.2909 | 30.5723 | Dzhus and Dmitrieva, 2001 |
| *V. longifolia* | Dzhus 1450, MSKU | 4x | Byelorussia |  |  | 52.0789 | 27.7157 | Dzhus and Dmitrieva, 2001 |
| *V. longifolia* | Dzhus 1295, MSKU | 4x | Byelorussia |  |  | 53.6541 | 26.7958 | Dzhus and Dmitrieva, 2001 |
| *V. longifolia* | Dmitrieva 300, MSK | 4x | Byelorussia |  |  | 54.4886 | 26.9849 | Dzhus and Dmitrieva, 2001 |
| *V. longifolia* | ? | 4x | Byelorussia |  |  | 54.7509 | 28.2792 | Parfenov and Dmitrieva, 1987 |
| *V. longifolia* | Dzhus 1234, MKSU | 4x | Byelorussia |  |  | 55.1755 | 30.1024 | Dzhus and Dmitrieva, 2001 |
| *V. longifolia* | Simonovich 653, MSK | 4x | Byelorussia |  |  | 55.9040 | 29.3524 | Dzhus and Dmitrieva, 2001 |
| *V. longifolia* | Travnicek 1993, OL | 4x | Czech Rep. |  |  | 48.6235 | 16.9397 | Travnicek, 2004 |
| *V. longifolia* | Travnicek 1995, OL | 4x | Czech Rep. |  |  | 48.7355 | 16.9935 | Travnicek, 2004 |
| *V. longifolia* | Travnicek et al.1994, OL | 4x | Czech Rep. |  |  | 48.8159 | 16.7778 | Travnicek, 2004 |
| *V. longifolia* | Travnicek 1999, OL | 4x | Czech Rep. |  |  | 48.8247 | 17.5252 | Travnicek, 2004 |
| *V. longifolia* | Travnicek 1999, OL | 4x | Czech Rep. |  |  | 48.8378 | 16.7443 | Travnicek, 2004 |
| *V. longifolia* | Travnicek et al.1994, OL | 4x | Czech Rep. |  |  | 48.8564 | 16.7232 | Travnicek, 2004 |
| *V. longifolia* | Travnicek 1995, OL | 4x | Czech Rep. |  |  | 48.8847 | 15.8475 | Travnicek, 2004 |
| *V. longifolia* | Travnicek 1996, OL | 4x | Czech Rep. |  |  | 48.8944 | 17.6063 | Travnicek, 2004 |
| *V. longifolia* | Travnieck 1994, OL | 4x | Czech Rep. |  |  | 48.9011 | 17.2658 | Travnicek, 2004 |
| *V. longifolia* | Stech 1994, OL | 4x | Czech Rep. |  |  | 49.0103 | 14.4628 | Travnicek, 2004 |
| *V. longifolia* | Travnicek 1993, OL | 4x | Czech Rep. |  |  | 49.2292 | 14.7129 | Travnicek, 2004 |
| *V. longifolia* | Travnicek 1995, OL | 4x | Czech Rep. |  |  | 49.2722 | 17.4880 | Travnicek, 2004 |
| *V. longifolia* | Travnicek 1993, OL | 4x | Czech Rep. |  |  | 49.3026 | 17.3478 | Travnicek, 2004 |
| *V. longifolia* | Travnicek, Koblizek 1993, OL | 4x | Czech Rep. |  |  | 49.3072 | 16.7457 | Travnicek, 2004 |
| *V. longifolia* | Travnicek 1995, OL | 4x | Czech Rep. |  |  | 49.3180 | 17.3478 | Travnicek, 2004 |
| *V. longifolia* | Travnicek 1995, OL | 4x | Czech Rep. |  |  | 49.3616 | 17.3301 | Travnicek, 2004 |
| *V. longifolia* | Travnicek 1993, OL | 4x | Czech Rep. |  |  | 49.3943 | 14.6795 | Travnicek, 2004 |
| *V. longifolia* | Kusak, OL | 4x | Czech Rep. |  |  | 49.7616 | 16.9645 | Travnicek, 2004 |
| *V. longifolia* | Travnicek 1993, OL | 4x | Czech Rep. |  |  | 50.0250 | 15.2269 | Travnicek, 2004 |
| *V. longifolia* | Travnicek 1994, OL | 4x | Czech Rep. |  |  | 50.1226 | 15.1765 | Travnicek, 2004 |
| *V. longifolia* | Travnicek et al.1993, OL | 4x | Czech Rep. |  |  | 50.2123 | 15.8787 | Travnicek, 2004 |
| *V. longifolia* | Stepankova 1993, OL | 4x | Czech Rep. |  |  | 50.2437 | 14.6100 | Travnicek, 2004 |
| *V. longifolia* | Rydlo 1993, OL | 4x | Czech Rep. |  |  | 50.2501 | 14.5632 | Travnicek, 2004 |
| *V. longifolia* | Travnicek 1993, OL | 4x | Czech Rep. |  |  | 50.2511 | 14.5472 | Travnicek, 2004 |
| *V. longifolia* | Rydlo 1993, OL | 4x | Czech Rep. |  |  | 50.2520 | 14.5705 | Travnicek, 2004 |
| *V. longifolia* | Travnicek et al.1993, OL | 4x | Czech Rep. |  |  | 50.2552 | 15.5058 | Travnicek, 2004 |
| *V. longifolia* | Rydlo 1993, OL | 4x | Czech Rep. |  |  | 50.2830 | 14.5833 | Travnicek, 2004 |
| *V. longifolia* | Travnicek et al.1993, OL | 4x | Czech Rep. |  |  | 50.2936 | 16.0664 | Travnicek, 2004 |
| *V. longifolia* | Rydlo 1993, OL | 4x | Czech Rep. |  |  | 50.3122 | 14.4985 | Travnicek, 2004 |
| *V. longifolia* | Travnicek et al.1993, OL | 4x | Czech Rep. |  |  | 50.3431 | 15.9403 | Travnicek, 2004 |
| *V. longifolia* | Rydlo 1993, OL | 4x | Czech Rep. |  |  | 50.4182 | 14.4178 | Travnicek, 2004 |
| *V. longifolia* | Travnicek 1994, OL | 4x | Czech Rep. |  |  | 50.6776 | 14.5870 | Travnicek, 2004 |
| *V. longifolia* | ?, H | 4x | Finland |  |  | 65.9685 | 29.2057 | Raitanen, 1967 |
| *V. longifolia* | Albach S847, OLD | 4x | Finland | **1.5681** | 4.90-5.10 | 66.2960 | 27.1380 | new result |
| *V. longifolia* | Albach S850, OLD | 4x | Germany | **1.6800** | 3.94-4.50 | 49.8414 | 8.4033 | new result |
| *V. longifolia* | Härle U2, TUB | 4x | Hungary |  |  | 47.5197 | 21.9658 | Graze, 1933 |
| *V. longifolia* | Albach 1519, OLD | 4x | Hungary | **1.4735** | 6.41 | 47.9220 | 20.5090 | new result |
| *V. longifolia* | Albach 1544, OLD | 4x | Hungary | **1.4617** | 4.31 | 48.0145 | 20.7207 | new result |
| *V. longifolia* | Höpke 363, OLD | 4x | Kazakhstan | **1.5658** | 7.08 | 51.8327 | 67.9495 | new result |
| *V. longifolia* | Höpke 366, OLD | 4x | Kazakhstan | **1.6237** | 6.56 | 51.8870 | 66.2356 | new result |
| *V. longifolia* | Höpke 351, OLD | 4x | Kazakhstan | **1.4535** | 6.91 | 52.5607 | 71.3342 | new result |
| *V. longifolia* | Höpke 357, OLD | 4x | Kazakhstan | **1.5578** | 7.72 | 52.8905 | 68.9758 | new result |
| *V. longifolia* | Albach S746, OLD | 4x | Mongolia | **1.5959** | 4.44 | 47.8333 | 106.9500 | new result |
| *V. longifolia* | Gadella 11.880-11.881, U | 4x | Netherlands |  |  | 52.5800 | 6.0800 | Gadella and Kliphuis, 1973 |
| *V. longifolia* | Schöngart, Kosachev 13, OLD | 4x | Russia | **1.6896** | 7.74 | 50.1645 | 86.4211 | new result |
| *V. longifolia* | Pfanzelt et al., 1149.4, OLD | 4x | Russia | **1.6894** | 6.07 | 50.6399 | 86.3131 | new result |
| *V. longifolia* | Pfanzelt et al., 1147.5, OLD | 4x | Russia | **1.6585** | 5.12 | 51.2524 | 82.5629 | new result |
| *V. longifolia* | Probatova 5076, VLA | 4x | Russia |  |  | 52.0800 | 139.9400 | Probatova and Sokolovskaya, 1989 |
| *V. longifolia* | Höpke 447, OLD | 4x | Russia | **1.5240** | 8.10 | 53.3015 | 80.9995 | new result |
| *V. longifolia* | ?, LE | 4x | Russia |  |  | 54.3349 | 109.5002 | Belaeva and Siplivinsky, 1975 |
| *V. longifolia* | Höpke 438, OLD | 4x | Russia | **1.5405** | 5.48 | 54.3350 | 80.6659 | new result |
| *V. longifolia* | Höpke 432, OLD | 4x | Russia | **1.4560** | 7.31 | 54.6972 | 79.1997 | new result |
| *V. longifolia* | Höpke 419, OLD | 4x | Russia | **1.5770** | 7.90 | 54.9843 | 72.0569 | new result |
| *V. longifolia* | Höpke 427, OLD | 4x | Russia | **1.5754** | 5.92 | 55.1252 | 75.5711 | new result |
| *V. longifolia* | Höpke 408, OLD | 4x | Russia | **1.4523** | 8.61 | 55.1931 | 64.9575 | new result |
| *V. longifolia* | Höpke 418, OLD | 4x | Russia | **1.6042** | 6.95 | 55.2434 | 71.2694 | new result |
| *V. longifolia* | Rudyka 4259, VLA | 4x | Russia |  |  | 55.4900 | 123.4700 | Probatova and Sokolovskaya, 1989 |
| *V. longifolia* | Höpke 417, OLD | 4x | Russia | **1.5324** | 6.09 | 55.5520 | 70.6755 | new result |
| *V. longifolia* | ? | 4x | Russia |  |  | 55.8200 | 48.8300 | Meshkova, 1965 |
| *V. longifolia* | Höpke 412, OLD | 4x | Russia | **1.4883** | 11.76 | 56.0342 | 69.1784 | new result |
| *V. longifolia* | Gnutikov536, VLA | 4x | Russia |  |  | 56.9689 | 97.5762 | Chepinoga et al., 2010 |
| *V. longifolia* | Albach S846, OLD | 4x | Russia | **1.6085** | 4.50-5.90 | 60.0000 | 89.0000 | new result |
| *V. longifolia* | Albach S372, OLD | 4x | Russia | **1.6913** | 4.86 | 61.2633 | 128.0722 | new result |
| *V. longifolia* | Danihelka 422, BRNU 619902 | 4x | Russia | **1.8450** | 6.68 | 61.2633 | 128.0722 | new result |
| *V. longifolia* | Albach S371, OLD | 4x | Russia | **1.7117** | 4.56 | 62.5353 | 130.0303 | new result |
| *V. longifolia* | ? | 4x | Russia |  |  | 63.1500 | 108.4300 | Krogulevich and Rostovtseva, 1984 |
| *V. longifolia* | Sokolovskaya 105, VLA | 4x | Russia |  |  | 69.4700 | 49.1200 | Sokolovskaya and Strelkova, 1960 |
| *V. longifolia* | Albach S774, OLD | 4x | Russia | **1.6581** | 2.80-3.70 |  |  | new result |
| *V. longifolia* | Travnicek 1995, OL | 4x | Slovakia |  |  | 47.7964 | 18.1082 | Travnicek, 2004 |
| *V. longifolia* | Albach 1509, OLD | 4x | Slovakia | **1.5871** | 4.51 | 48.2516 | 16.9550 | new result |
| *V. longifolia* | Travnicek 1995, OL | 4x | Slovakia |  |  | 48.3368 | 16.8993 | Travnicek, 2004 |
| *V. longifolia* | Travnicek 1995, OL | 4x | Slovakia |  |  | 48.4443 | 19.7619 | Travnicek, 2004 |
| *V. longifolia* | Travnicek 1994, OL | 4x | Slovakia |  |  | 48.4984 | 21.9421 | Travnicek, 2004 |
| *V. longifolia* | Travnicek 1991, OL | 4x | Slovakia |  |  | 48.5020 | 22.0474 | Travnicek, 2004 |
| *V. longifolia* | Travnicek 1995, OL | 4x | Slovakia |  |  | 48.5134 | 16.9256 | Travnicek, 2004 |
| *V. longifolia* | ? | 4x | Slovakia |  |  | 48.5331 | 16.9990 | Travnicek, 2004 |
| *V. longifolia* | Albach 1516, OLD | 4x | Slovakia | **1.5734** | 4.68 | 48.5346 | 17.0045 | new result |
| *V. longifolia* | Travnicek 1994, OL | 4x | Slovakia |  |  | 48.5639 | 21.9548 | Travnicek, 2004 |
| *V. longifolia* | Majovsky, Murin, SLO | 4x | Slovakia |  |  | 48.6064 | 22.0009 | Murín and Majovsky, 1987 |
| *V. longifolia* | Travnicek 1995, OL | 4x | Slovakia |  |  | 48.6923 | 22.0864 | Travnicek, 2004 |
| *V. longifolia* | Travnicek 1995, OL | 4x | Slovakia |  |  | 48.7252 | 17.0656 | Travnicek, 2004 |
| *V. longifolia* | Albach 1515, OLD | 4x | Slovakia | **1.5098** | 5.43 | 48.8548 | 17.1687 | new result |
| *V. longifolia* | Záborsky s.n., SLO | 4x | Slovakia |  |  | 48.8680 | 17.1960 | Löve, 1980 |
| *V. longifolia* | Höpke 305, OLD | 4x | Ukraine | **1.3392** | 8 | 47.3086 | 37.0783 | new result |
| *V. longifolia* | Höpke 310, OLD | 4x | Ukraine | **1.5872** | 6.62 | 48.7646 | 35.4366 | new result |
| *V. longifolia* | Boiko 21-32, OLD, KW | 4x | Ukraine | **1.4890** | 3.72-4.64 | 49.0455 | 37.4852 | new result |
| *V. longifolia* | Albach 1485, OLD | 4x | Ukraine | **1.5279** | 4.60 | 49.5499 | 27.0774 | new result |
| *V. longifolia* | Albach 1450, OLD | 4x | Ukraine | **1.4800** | 4.44 | 50.3294 | 30.5787 | new result |
| *V. longifolia* | Höpke 286, OLD | 4x | Ukraine | **1.3662** | 7.26 | 50.5322 | 30.5204 | new result |
| *V. longifolia* | Höpke 285, OLD | 4x | Ukraine | **1.4520** | 7.38 | 50.5322 | 30.5204 | new result |
| *V. longifolia* | Höpke 287, OLD | 4x | Ukraine | **1.3850** | 8.08 | 50.5325 | 30.5250 | new result |
| *V. longifolia* | Höpke 281, OLD | 4x | Ukraine | **1.4268** | 3.52 | 50.5335 | 30.5265 | new result |
| *V. longifolia* | Albach 1460, OLD | 4x | Ukraine | **1.5389** | 3.87 | 50.6001 | 30.5858 | new result |
| *V. longifolia* | Albach 1456, OLD | 4x | Ukraine | **1.5003** | 4.88 | 50.7375 | 30.6694 | new result |
| *V. spicata* | Bardy pseu61, WU | 2x | Austria |  |  | 46.8975 | 13.2700 | Bardy et al., 2011 |
| *V. spicata* | Bardy pseu60, WU | 2x | Austria |  |  | 46.9383 | 13.1594 | Bardy et al., 2011 |
| *V. spicata* | Fischer 18.6.1963, W | 2x | Austria |  |  | 47.9539 | 16.7740 | Fischer, 1969 |
| *V. spicata^4^* | Fischer 3.8.1972, WU | 2x | Bosnia-Hercegovina |  |  | 43.7900 | 18.3100 | Fischer, 1974 |
| *V. spicata^1^* | Turrill 3002, TUB | 2x | Bulgaria |  |  | 43.1400 | 27.8800 | Graze, 1933 |
| *V. spicata* | Albach S758, OLD | 2x | France | **0.6978** | 3.55 | 44.9660 | 6.6750 | new result |
| *V. spicata* | Albach S758, OLD | 2x | France | **0.7350** | 2.70 | 44.9660 | 6.6750 | new result |
| *V. spicata* | VSPCA4 | 2x | France | **0.6316** | 9.59 | 47.6298 | 7.5348 | new result |
| *V. spicata* | Albach S492, OLD | 2x | Germany | **0.6637** | 2.79 | 52.4815 | 14.5360 | new result |
| *V. spicata* | Kliphuis 11490, U | 2x | Hungary |  |  | 47.1284 | 19.4017 | Löve, 1977 |
| *V. spicata* | Monti, 10.7.1980, PI | 2x | Italy |  |  | 44.1145 | 10.1409 | Giordani, 1980 |
| *V. spicata* | Höpke 334, OLD | 2x | Kazakhstan | **0.6674** | 6.74 | 51.6984 | 74.4450 | new result |
| *V. spicata* | Höpke 365, OLD | 2x | Kazakhstan | **0.8915** | 8.08 | 51.8327 | 67.9495 | new result |
| *V. spicata* | Höpke 340, OLD | 2x | Kazakhstan | **0.6821** | 6.15 | 52.0289 | 72.7075 | new result |
| *V. spicata* | Höpke 342, OLD | 2x | Kazakhstan | **0.6844** | 8.37 | 52.1542 | 72.7378 | new result |
| *V. spicata* | Höpke 367, OLD | 2x | Kazakhstan | **0.7480** | 7.07 | 52.2333 | 65.1318 | new result |
| *V. spicata* | Höpke 369, OLD | 2x | Kazakhstan | **0.6545** | 5.14 | 52.4528 | 64.0844 | new result |
| *V. spicata* | Höpke 344, OLD | 2x | Kazakhstan | **0.7058** | 6.92 | 52.4798 | 71.9413 | new result |
| *V. spicata* | Höpke 350, OLD | 2x | Kazakhstan | **0.6538** | 5.30 | 52.5607 | 71.3342 | new result |
| *V. spicata* | Höpke 360, OLD | 2x | Kazakhstan | **0.6410** | 7.51 | 52.7259 | 69.0837 | new result |
| *V. spicata* | Höpke 353, OLD | 2x | Kazakhstan | **0.6718** | 7.32 | 52.8325 | 69.7847 | new result |
| *V. spicata* | Höpke 372, OLD | 2x | Kazakhstan | **0.7110** | 7.60 | 53.2528 | 63.4950 | new result |
| *V. spicata* | Höpke 375, OLD | 2x | Kazakhstan | **0.7616** | 7.95 | 53.5938 | 62.4794 | new result |
| *V. spicata* | ? | 2x | Poland |  |  | 50.0046 | 19.8103 | Pogan et al., 1982 |
| *V. spicata* | ? | 2x | Poland |  |  | 50.0351 | 19.8670 | Pogan et al., 1982 |
| *V. spicata* | ? | 2x | Poland |  |  | 50.4176 | 20.6748 | Pogan et al., 1982 |
| *V. spicata* | Albach S376, OLD | 2x | Romania | **0.6549** | 3.78 | 46.6000 | 25.4000 | new result |
| *V. spicata* | Höpke 238, OLD | 2x | Russia | **0.7359** | 7.21 | 46.6290 | 42.4656 | new result |
| *V. spicata* | Kamelin et al., 3.7.1996, ALTB | 2x | Russia | **0.8000** | 10.05 | 50.7750 | 82.0660 | Kosachev et al., 2016 |
| *V. spicata* | Kosachev 12.7.2012, ALTB | 2x | Russia | **0.6740** | 8.80 | 50.9156 | 82.3265 | Kosachev et al., 2016 |
| *V. spicata* | Pfanzelt et al., 1138.1, OLD | 2x | Russia | **0.6125** | 4.99 | 50.9158 | 82.3274 | new result |
| *V. spicata* | Kamelin et al., 1998, ALTB | 2x | Russia | **0.7254** | 7.70-9.40 | 51.0573 | 82.2655 | Kosachev and Albach 2015 |
| *V. spicata* | Kamelin 28.6.1998, ALTB | 2x | Russia | **0.7280** | 8.56 | 51.0832 | 82.2831 | Kosachev et al., 2016 |
| *V. spicata^3^* | Pfanzelt et al., 1146.1, OLD | 2x | Russia | **0.6023** | 5.55 | 51.2527 | 82.5607 | new result |
| *V. spicata* | Kosachev 14.7.2012, ALTB | 2x | Russia | **0.6750** | 6.99 | 51.2948 | 82.4792 | Kosachev et al., 2016 |
| *V. spicata* | Pfanzelt 1144, OLD | 2x | Russia | **0.5492** | 7.59 | 51.2964 | 82.4910 | new result |
| *V. spicata* | Pfanzelt et al., 1141.1, OLD | 2x | Russia | **0.6632** | 4.74 | 51.2964 | 82.4908 | new result |
| *V. spicata* | Pfanzelt et al., 1135b, OLD | 2x | Russia | **0.5781** | 7.14 | 51.3924 | 82.2084 | new result |
| *V. spicata* | Pfanzelt et al., 1135a, OLD | 2x | Russia | **0.5885** | 8.14 | 51.3924 | 82.2084 | new result |
| *V. spicata^5^* | Djaczenko 11.9.1996, ALTB | 2x | Russia | **0.6100** | 11.58 | 51.6659 | 79.7404 | Kosachev et al., 2016 |
| *V. spicata* | Kamelin et al., 1996, ALTB | 2x | Russia | **0.7480** | 10.80-14.60 | 51.7642 | 82.9063 | Kosachev and Albach, 2015 |
| *V. spicata* | Pfanzelt et al., 1127.1, OLD | 2x | Russia | **0.6231** | 6.16 | 51.7684 | 82.1383 | new result |
| *V. spicata* | Kamelin et al., 28.6.1996, ALTB | 2x | Russia | **0.7310** | 9.70 | 51.8329 | 82.9500 | Kosachev et al., 2016 |
| *V. spicata* | ? | 2x | Russia |  |  | 51.9800 | 84.9300 | Rostovtseva 1977 |
| *V. spicata* | Schöngart, Kosachev 20, OLD | 2x | Russia | **0.6047** | 3.98 | 52.5214 | 85.2770 | new result |
| *V. spicata^5^* | Kosachev 8.9.2012, OLD | 2x | Russia | **0.6650** |  | 52.6710 | 82.0707 | Kosachev and Albach, 2015 |
| *V. spicata* | Kamelin et al., 1998, ALTB | 2x | Russia | **0.7380** | 9.52 | 52.7500 | 83.2833 | Kosachev et al., 2016 |
| *V. spicata* | Kamelin et al., 1998, ALTB | 2x | Russia | **0.7717** | >10 | 52.7512 | 83.3950 | Kosachev and Albach, 2015 |
| *V. spicata^5^* | Kharvryuk 10.7.2001, ALTB | 2x | Russia | **0.6960** | >10 | 53.2605 | 83.6791 | Kosachev et al., 2016 |
| *V. spicata* | Kharvryuk 2001, ALTB | 2x | Russia | **0.7552** | 9.70-12.70 | 53.2605 | 83.6791 | Kosachev and Albach, 2015 |
| *V. spicata^3^* | Kosachev kam.2.1, ALTB | 2x | Russia | **0.7330** | >10 | 53.2633 | 83.6700 | new result |
| *V. spicata^3^* | Kosachev Jun. 2000, ALTB | 2x | Russia | **0.7723** | >10 | 53.2633 | 83.6700 | Kosachev et al., 2016 |
| *V. spicata^5^* | Albach S628, OLD | 2x | Russia | **0.6458** | 5.05 | 53.2646 | 83.6774 | new result |
| *V. spicata^5^* | Pfanzelt et al., 1124.3, OLD | 2x | Russia | **0.6123** | 6.71 | 53.2647 | 83.6675 | new result |
| *V. spicata* | Höpke 326, OLD | 2x | Russia | **0.6476** | 5.78 | 53.2664 | 79.5154 | new result |
| *V. spicata^5^* | Pfanzelt et al., 1120, OLD | 2x | Russia | **0.5683** | 7.90 | 53.2666 | 83.6702 | new result |
| *V. spicata^5^* | Pfanzelt et al., 1123.1, OLD | 2x | Russia | **0.5818** | 6.33 | 53.2666 | 83.6702 | new result |
| *V. spicata^5^* | Albach S634, OLD | 2x | Russia | **0.6442** | 4.19 | 53.2769 | 83.7374 | new result |
| *V. spicata* | Höpke 446, OLD | 2x | Russia | **0.7263** | 11 | 53.3403 | 80.9568 | new result |
| *V. spicata* | Höpke 448, OLD | 2x | Russia | **0.7077** | 10.50 | 53.4551 | 81.4138 | new result |
| *V. spicata* | Höpke 444, OLD | 2x | Russia | **0.7708** | 9.85 | 53.4970 | 80.7278 | new result |
| *V. spicata* | BRNU 591078 | 2x | Russia | **0.8249** | 9.41 | 53.5542 | 56.1056 | new result |
| *V. spicata* | Höpke 443, OLD | 2x | Russia | **0.7653** | 12.70 | 53.6385 | 80.9397 | new result |
| *V. spicata* | Höpke 379, OLD | 2x | Russia | **0.7049** | 8.03 | 54.1250 | 61.0388 | new result |
| *V. spicata* | Höpke 440, OLD | 2x | Russia | **0.6791** | 10.19 | 54.3237 | 81.5449 | new result |
| *V. spicata* | Höpke 437, OLD | 2x | Russia | **0.7227** | 12.31 | 54.3350 | 80.6659 | new result |
| *V. spicata* | Höpke 404, OLD | 2x | Russia | **0.7164** | 10.30 | 54.3927 | 62.7376 | new result |
| *V. spicata* | Höpke 435, OLD | 2x | Russia | **0.6534** | 5.07 | 54.6972 | 79.1997 | new result |
| *V. spicata* | Höpke 431, OLD | 2x | Russia | **0.6179** | 6.03 | 54.8897 | 78.3653 | new result |
| *V. spicata* | Höpke 421, OLD | 2x | Russia | **0.6790** | 8.21 | 54.9688 | 72.5621 | new result |
| *V. spicata* | Höpke 424, OLD | 2x | Russia | **0.6320** | 8.40 | 54.9895 | 74.2278 | new result |
| *V. spicata* | Höpke 425, OLD | 2x | Russia | **0.6421** | 6.42 | 55.1252 | 75.5711 | new result |
| *V. spicata^5^* | Höpke 407, OLD | 2x | Russia | **0.6830** | 6.87 | 55.1931 | 64.9575 | new result |
| *V. spicata* | Höpke 411, OLD | 2x | Russia | **0.6999** | 8.44 | 55.2683 | 67.2875 | new result |
| *V. spicata* | Höpke 430, OLD | 2x | Russia | **0.6641** | 6.32 | 55.2727 | 78.2853 | new result |
| *V. spicata* | Höpke 429, OLD | 2x | Russia | **0.6386** | 4.40 | 55.3495 | 76.2579 | new result |
| *V. spicata* | Höpke 409, OLD | 2x | Russia | **0.7771** | 9.72 | 55.4063 | 66.2335 | new result |
| *V. spicata* | Höpke 414, OLD | 2x | Russia | **0.7350** | 9.44 | 56.0342 | 69.1784 | new result |
| *V. spicata* | ?, SARA | 2x | Serbia | **0.7200** | - | 44.1100 | 19.9800 | Pustahija et al., 2013 |
| *V. spicata^3^* | Frajman et al., 11726, WU | 2x | Serbia |  |  | 45.1100 | 19.6681 | Bardy et al., 2011 |
| *V. spicata* | Albach S482, OLD | 2x | Slovakia | **0.8375** | 3.38 | 48.4300 | 17.3000 | new result |
| *V. spicata* | Travnicek 1995, OL | 2x | Slovakia |  |  | 49.0000 | 22.5224 | Travnicek, 2004 |
| *V. spicata* | Delgado et al., SALA 105779 | 2x | Spain |  |  | 42.8991 | -0.7830 |  |
| *V. spicata* | Albach S853, OLD | 2x | Switzerland | **0.7200** | 3.72-5.60 | 46.1055 | 7.0698 | new result |
| *V. spicata* | Favarger Sept. 1952, NEU | 2x | Switzerland |  |  | 46.1147 | 7.0117 | Brandt, 1953 |
| *V. spicata* | Härle S1, TUB | 2x | Switzerland |  |  | 46.2800 | 7.8800 | Graze, 1933 |
| *V. spicata* | Albach S526, OLD | 2x | Switzerland | **0.7854** | 3.16 | 46.7500 | 9.9500 | new result |
| *V. spicata* | Albach S751, OLD | 2x | Switzerland | **0.7942** | 4.27 | 46.7500 | 9.9500 | new result |
| *V. spicata* | ?, NEU | 2x | Switzerland |  |  | 46.7577 | 7.1034 | Brandt, 1953 |
| *V. spicata* | ?, NEU | 2x | Switzerland |  |  | 47.0245 | 6.9438 | Brandt, 1953 |
| *V. spicata* | Ryff s.n., OLD | 2x | Ukraine | **0.6488** | 9.63 | 44.9775 | 35.1554 | new result |
| *V. spicata^6^* | Albach 1463, OLD | 2x | Ukraine | **0.6130** | 5.67 | 48.2472 | 30.1933 | new result |
| *V. spicata^6^* | Höpke 312, OLD | 2x | Ukraine | **0.5808** | 3.97 | 48.7579 | 35.4301 | new result |
| *V. spicata* | Höpke 311, OLD | 2x | Ukraine | **0.5987** | 4.42 | 48.7596 | 35.4333 | new result |
| *V. spicata* | Albach 1476, OLD | 2x | Ukraine | **0.6259** | 4.35 | 48.7911 | 26.6314 | new result |
| *V. spicata* | Albach 1469, OLD | 2x | Ukraine | **0.6367** | 3.83 | 48.7930 | 29.1114 | new result |
| *V. spicata* | Albach 1471, OLD | 2x | Ukraine | **0.6225** | 4.62 | 48.8015 | 26.5983 | new result |
| *V. spicata* | Albach 1484, OLD | 2x | Ukraine | **0.6203** | 4.75 | 48.8172 | 25.8297 | new result |
| *V. spicata* | Höpke 294, OLD | 2x | Ukraine | **0.6045** | 1.93 | 49.3537 | 30.1198 | new result |
| *V. spicata* | Höpke 295, OLD | 2x | Ukraine | **0.5959** | 3.53 | 49.3540 | 30.1206 | new result |
| *V. spicata* | Mosyakin, OLD 2912 | 2x | Ukraine | **0.5819** | 8.20 | 49.6374 | 31.5026 | new result |
| *V. spicata* | Mosyakin, OLD 2913 | 2x | Ukraine | **0.6360** | 6.28 | 49.7169 | 31.5436 | new result |
| *V. spicata^5^* | Mosyakin, OLD 2900 | 2x | Ukraine | **0.5795** | 5.02 | 49.8163 | 31.5826 | new result |
| *V. spicata^5^* | Mosyakin, OLD 2921 | 2x | Ukraine | **0.5949** | 4.91 | 49.8163 | 31.5826 | new result |
| *V. spicata^5^* | Mosyakin, OLD 2922 | 2x | Ukraine | **0.6122** | 6.78 | 49.8163 | 31.5826 | new result |
| *V. spicata^5^* | Albach et al., 1445, OLD | 2x | Ukraine | **0.5963** | 5.00 | 50.2937 | 30.5800 | new result |
| *V. spicata* | Albach 1451, OLD | 2x | Ukraine | **0.6109** | 5.04 | 50.3294 | 30.5787 | new result |
| *V. spicata* | Albach 1449, OLD | 2x | Ukraine | **0.6102** | 4.63 | 50.3299 | 30.5677 | new result |
| *V. spicata* | Albach 1486, OLD | 2x | Ukraine | **0.5951** | 4.97 | 50.3299 | 30.5677 | new result |
| *V. spicata* | Höpke 280, OLD | 2x | Ukraine | **0.5846** | 2.73 | 50.5315 | 30.5236 | new result |
| *V. spicata* | Höpke 288, OLD | 2x | Ukraine | **0.6378** | 8.44 | 50.5325 | 30.5250 | new result |
| *V. spicata* | Höpke 284, OLD | 2x | Ukraine | **0.5945** | 5.80 | 50.5335 | 30.5265 | new result |
| *V. spicata^5^* | Höpke 293, OLD | 2x | Ukraine | **0.6104** | 6.04 | 50.7162 | 30.3840 | new result |
| *V. spicata^6^* | Höpke 292, OLD | 2x | Ukraine | **0.6191** | 7.66 | 50.7162 | 30.3840 | new result |
| *V. spicata^5^* | Albach 1455, OLD | 2x | Ukraine | **0.6156** | 4.61 | 50.7367 | 30.6605 | new result |
| *V. spicata* | Travnicek 1995, OL | 4x | Austria |  |  | 47.7596 | 16.8648 | Travnicek, 2004 |
| *V. spicata* | Bardy pseu63, WU | 4x | Austria |  |  | 47.9667 | 16.1956 | Bardy et al., 2011 |
| *V. spicata* | bardy au20-1, WU | 4x | Austria | **1.4064** | 4.86 | 48.0964 | 16.9597 | new result |
| *V. spicata* | Bardy pseu65, WU | 4x | Austria |  |  | 48.1239 | 16.9342 | Bardy et al., 2011 |
| *V. spicata* | Kriechbaum 19.7.2016, OLD | 4x | Austria | **1.4338** | 6.92 | 48.1534 | 16.9578 | new result |
| *V. spicata* | Härle 01, TUB | 4x | Austria |  |  | 48.3080 | 16.4310 | Graze, 1933 |
| *V. spicata* | Albach S749, OLD | 4x | Austria | **1.4136** | 4.23 | 48.4000 | 15.5500 | new result |
| *V. spicata* | Bardy au27-1, WU | 4x | Austria | **1.4602** | 5.45 | 48.4144 | 15.5000 | new result |
| *V. spicata* | Bardy-Durchhalter, pseu62, WU | 4x | Austria |  |  | 48.7625 | 15.9439 | Bardy et al., 2011 |
| *V. spicata* | No Voucher | 4x | Britain |  |  | 51.4557 | -2.6300 | Walters, 1954 |
| *V. spicata* | No Voucher | 4x | Britain |  |  | 52.5180 | 0.7450 | Walters, 1954 |
| *V. spicata* | Dzhus 130, MSKU | 4x | Byelorussia |  |  | 52.4154 | 24.3396 | Dzhus and Dmitrieva, 2001 |
| *V. spicata* | Dzhus 2634, MSKU | 4x | Byelorussia |  |  | 52.6259 | 29.6064 | Dzhus and Dmitrieva, 2001 |
| *V. spicata^5^* | Dzhus 2836, MSKU | 4x | Byelorussia |  |  | 52.6259 | 29.6064 | Dzhus and Dmitrieva, 2001 |
| *V. spicata* | Dzhus 1794, MSKU | 4x | Byelorussia |  |  | 53.7852 | 27.4098 | Dzhus and Dmitrieva, 2001 |
| *V. spicata* | Dzhus 1891, MSKU | 4x | Byelorussia |  |  | 54.4598 | 26.0956 | Dzhus and Dmitrieva, 2001 |
| *V. spicata* | Dzhus 1856, MSKU | 4x | Byelorussia |  |  | 54.4974 | 26.2602 | Dzhus and Dmitrieva, 2001 |
| *V. spicata* | ? | 4x | Byelorussia |  |  | 54.7715 | 28.2775 | Dmitrieva, 1986 |
| *V. spicata* | Pluhar 1995, OL | 4x | Czech Rep. |  |  | 48.6464 | 16.9313 | Travnicek, 2004 |
| *V. spicata* | Pluhar 1995, OL | 4x | Czech Rep. |  |  | 48.7220 | 16.9392 | Travnicek, 2004 |
| *V. spicata* | Travnicek 1994, OL | 4x | Czech Rep. |  |  | 48.7479 | 16.7800 | Travnicek, 2004 |
| *V. spicata* | Travnicek 1995, OL | 4x | Czech Rep. |  |  | 48.7897 | 16.6308 | Travnicek, 2004 |
| *V. spicata* | Travnicek 1995, OL | 4x | Czech Rep. |  |  | 48.8063 | 16.6469 | Travnicek, 2004 |
| *V. spicata* | Travnicek 1993, OL | 4x | Czech Rep. |  |  | 48.8196 | 16.0105 | Travnicek, 2004 |
| *V. spicata* | Travnicek et al.1994, OL | 4x | Czech Rep. |  |  | 48.8227 | 16.6780 | Travnicek, 2004 |
| *V. spicata* | Travnicek 1999, OL | 4x | Czech Rep. |  |  | 48.8361 | 16.7425 | Travnicek, 2004 |
| *V. spicata* | Travnicek et al.1994, OL | 4x | Czech Rep. |  |  | 48.8498 | 16.6991 | Travnicek, 2004 |
| *V. spicata* | Travnicek 1995, OL | 4x | Czech Rep. |  |  | 48.8703 | 17.1486 | Travnicek, 2004 |
| *V. spicata* | Travnicek 1992, OL | 4x | Czech Rep. |  |  | 48.8765 | 15.8771 | Travnicek, 2004 |
| *V. spicata* | Travnicek 1994, OL | 4x | Czech Rep. |  |  | 49.1022 | 16.2604 | Travnicek, 2004 |
| *V. spicata* | Travnicek 1994, OL | 4x | Czech Rep. |  |  | 49.1064 | 16.1887 | Travnicek, 2004 |
| *V. spicata* | Travnicek 1993, OL | 4x | Czech Rep. |  |  | 49.2396 | 16.7016 | Travnicek, 2004 |
| *V. spicata* | Travnicek 1998, OL | 4x | Czech Rep. |  |  | 49.3409 | 17.0733 | Travnicek, 2004 |
| *V. spicata* | Travnicek 1994, OL | 4x | Czech Rep. |  |  | 49.3834 | 17.1252 | Travnicek, 2004 |
| *V. spicata* | Travnicek 1993, OL | 4x | Czech Rep. |  |  | 49.4616 | 16.9807 | Travnicek, 2004 |
| *V. spicata* | Travnicek 1993, OL | 4x | Czech Rep. |  |  | 49.4675 | 16.9927 | Travnicek, 2004 |
| *V. spicata* | Travnicek 1993, OL | 4x | Czech Rep. |  |  | 49.4910 | 17.0105 | Travnicek, 2004 |
| *V. spicata* | Travnicek 1993, OL | 4x | Czech Rep. |  |  | 49.5552 | 17.0897 | Travnicek, 2004 |
| *V. spicata* | Travnicek 1993, OL | 4x | Czech Rep. |  |  | 49.5932 | 17.0227 | Travnicek, 2004 |
| *V. spicata* | Travnicek 1994, OL | 4x | Czech Rep. |  |  | 49.9501 | 14.1413 | Travnicek, 2004 |
| *V. spicata* | Travnicek 1993, OL | 4x | Czech Rep. |  |  | 50.2753 | 14.2971 | Travnicek, 2004 |
| *V. spicata* | Travnicek 1993, OL | 4x | Czech Rep. |  |  | 50.3347 | 14.6700 | Travnicek, 2004 |
| *V. spicata* | Travnicek 1997, OL | 4x | Czech Rep. |  |  | 50.3880 | 14.2550 | Travnicek, 2004 |
| *V. spicata* | Stepankova 1993, OL | 4x | Czech Rep. |  |  | 50.4861 | 14.5071 | Travnicek, 2004 |
| *V. spicata* | Hrouda 1993, OL | 4x | Czech Rep. |  |  | 50.4913 | 14.4831 | Travnicek, 2004 |
| *V. spicata* | Sadlo 1993, OL | 4x | Czech Rep. |  |  | 50.5311 | 14.4067 | Travnicek, 2004 |
| *V. spicata* | Travnicek 1997, OL | 4x | Czech Rep. |  |  | 50.5499 | 14.0494 | Travnicek, 2004 |
| *V. spicata* | Kukkonen 12881, H | 4x | Finland |  |  | 60.0780 | 19.9946 | Kukkonen, 1986 |
| *V. spicata* | Kukkonen 11310, H | 4x | Finland |  |  | 60.3004 | 23.5167 | Uotila and Pellinen, 1985 |
| *V. spicata* | H | 4x | Finland |  |  | 60.4072 | 22.4768 | Raitanen, 1967 |
| *V. spicata* | Kukkonen s.n., H | 4x | Finland |  |  | 60.4072 | 22.4768 | Kukkonen, 1986 |
| *V. spicata* | Kukkonen 12863, H | 4x | Finland |  |  | 60.4236 | 20.2574 | Kukkonen, 1986 |
| *V. spicata* | Kukkonen 12865, H | 4x | Finland |  |  | 60.4236 | 20.2574 | Kukkonen, 1986 |
| *V. spicata* | Kukkonen 12870, H | 4x | Finland |  |  | 60.4236 | 20.2574 | Kukkonen, 1986 |
| *V. spicata* | Kukkonen 11327, H | 4x | Finland |  |  | 60.4451 | 26.4115 | Kukkonen, 1986 |
| *V. spicata* | Kukkonen 11234, H | 4x | Finland |  |  | 60.5035 | 22.0613 | Kukkonen, 1986 |
| *V. spicata* | Kukkonen 11245, H | 4x | Finland |  |  | 60.5035 | 22.0613 | Kukkonen, 1986 |
| *V. spicata* | Kukkonen 11832, H | 4x | Finland |  |  | 60.5035 | 22.0613 | Kukkonen, 1986 |
| *V. spicata* | Kukkonen 12852, H | 4x | Finland |  |  | 60.5035 | 22.0613 | Uotila and Pellinen, 1985 |
| *V. spicata* | H | 4x | Finland |  |  | 60.5159 | 22.0354 | Raitanen, 1967 |
| *V. spicata* | VSWEST2 | 4x | France | **1.3004** | 8.76 | 47.9600 | 7.2600 | new result |
| *V. spicata* | Bertsch, TUB | 4x | Germany |  |  | 48.0499 | 9.3272 | Huber, 1927 |
| *V. spicata* | Dersch | 4x | Germany |  |  | 49.7875 | 7.9375 | Paule et al., 2016 |
| *V. spicata* | Bardy pseu66, WU | 4x | Hungary |  |  | 46.9047 | 17.5225 | Bardy et al., 2011 |
| *V. spicata^2^* | Bardy hu21-1, WU | 4x | Hungary | **1.5092** | 9.36 | 47.0419 | 17.7122 | new result |
| *V. spicata* | Albach 1551, OLD | 4x | Hungary | **1.4502** | 4.05 | 47.2846 | 17.0400 | new result |
| *V. spicata* | Albach 1551, OLD | 4x | Hungary | **1.4502** | 4.05 | 47.2846 | 17.0400 | new result |
| *V. spicata* | Albach 1552, OLD | 4x | Hungary | **1.4580** | 4.00 | 47.2944 | 17.1046 | new result |
| *V. spicata* | Albach 1552, OLD | 4x | Hungary | **1.4580** | 4.00 | 47.2944 | 17.1046 | new result |
| *V. spicata* | Albach S869, OLD | 4x | Hungary | **1.4960** | 4.22-4.64 | 47.3841 | 18.4353 | new result |
| *V. spicata* | Bardy pseu64, WU | 4x | Hungary |  |  | 47.6769 | 16.6769 | Bardy et al., 2011 |
| *V. spicata* | Härle U7, TUB | 4x | Hungary |  |  | 47.6800 | 21.7400 | Graze, 1933 |
| *V. spicata* | Albach 1545, OLD | 4x | Hungary | **1.4695** | 5.14 | 47.8060 | 19.9922 | new result |
| *V. spicata* | Albach 1520, OLD | 4x | Hungary | **1.3607** | 5.01 | 47.9220 | 20.5117 | new result |
| *V. spicata* | Bardy hu05-1, WU | 4x | Hungary | **1.5272** | 7.57 | 47.9356 | 20.1967 | Bardy et al., 2011 |
| *V. spicata* | Albach 1526.1, OLD | 4x | Hungary | **1.4892** | 3.65 | 47.9568 | 20.5284 | new result |
| *V. spicata* | Albach 1534, OLD | 4x | Hungary | **1.3919** | 5.60 | 48.0567 | 20.4633 | new result |
| *V. spicata^2^* | Albach 1530, OLD | 4x | Hungary | **1.4114** | 5.20 | 48.0905 | 20.4326 | new result |
| *V. spicata* | Albach 1539, OLD | 4x | Hungary | **1.4282** | 5.28 | 48.1307 | 20.6821 | new result |
| *V. spicata* | Engelskjon 1635, O | 4x | Norway |  |  | 59.9700 | 10.4800 | Engelskjon, 1979 |
| *V. spicata* | ? | 4x | Poland |  |  | 50.1508 | 19.7522 | Pogan et al., 1982 |
| *V. spicata* | ? | 4x | Poland |  |  | 50.1529 | 19.7848 | Pogan et al., 1982 |
| *V. spicata* | Albach S542, OLD | 4x | Poland | **1.4375** | 3.65 | 50.8000 | 23.9666 | new result |
| *V. spicata* | Albach S422, OLD | 4x | Poland | **1.6275** | 2.80 | 52.2666 | 20.8666 | new result |
| *V. spicata* | Höpke 269, OLD | 4x | Romania | **1.5831** | 4.29 | 44.9817 | 21.9798 | new result |
| *V. spicata^2^* | Bardy pseu72, WU | 4x | Romania |  |  | 45.6339 | 25.5928 | Bardy et al., 2011 |
| *V. spicata* | Bardy pseu27, WU | 4x | Romania |  |  | 45.8100 | 24.2067 | Bardy et al., 2011 |
| *V. spicata^2^* | Bardy pseu71, WU | 4x | Romania |  |  | 46.8386 | 23.1250 | Bardy et al., 2011 |
| *V. spicata* | Bardy pseu70, WU | 4x | Romania |  |  | 46.9253 | 23.0819 | Bardy et al., 2011 |
| *V. spicata* | Höpke 226, OLD | 4x | Russia | **1.3958** | 5.65 | 47.6012 | 40.3253 | new result |
| *V. spicata* | Höpke 223, OLD | 4x | Russia | **1.4018** | 5.75 | 47.6012 | 40.3253 | new result |
| *V. spicata* | ? | 4x | Russia |  |  | 55.9000 | 48.7300 | Meshkova, 1965 |
| *V. spicata* | Frajman et al., 11719, WU | 4x | Serbia |  |  | 43.6675 | 20.5314 | Bardy et al., 2011 |
| *V. spicata^2^* | Travnicek 1993, OL | 4x | Slovakia |  |  | 48.1649 | 18.9868 | Travnicek, 2004 |
| *V. spicata* | Travnicek 1993, OL | 4x | Slovakia |  |  | 48.1658 | 18.9882 | Travnicek, 2004 |
| *V. spicata^2^* | Travnicek 1994, OL | 4x | Slovakia |  |  | 48.1952 | 19.9065 | Travnicek, 2004 |
| *V. spicata* | Albach 1511, OLD | 4x | Slovakia | **1.4497** | 4.22 | 48.2026 | 17.0061 | new result |
| *V. spicata^2^* | Travnicek 1994, OL | 4x | Slovakia |  |  | 48.2042 | 19.9694 | Travnicek, 2004 |
| *V. spicata* | Palkova, SLO | 4x | Slovakia |  |  | 48.2293 | 17.2231 | Majovsky, 1970 |
| *V. spicata^2^* | Travnicek 1993, OL | 4x | Slovakia |  |  | 48.2922 | 19.8281 | Travnicek, 2004 |
| *V. spicata* | Kusak, OL | 4x | Slovakia |  |  | 48.3445 | 18.5936 | Travnicek, 2004 |
| *V. spicata^2^* | Travnicek 1991, OL | 4x | Slovakia |  |  | 48.3549 | 20.0001 | Travnicek, 2004 |
| *V. spicata^2^* | Travnicek 1994, OL | 4x | Slovakia |  |  | 48.4023 | 18.8783 | Travnicek, 2004 |
| *V. spicata* | Kusak, OL | 4x | Slovakia |  |  | 48.4259 | 21.7829 | Travnicek, 2004 |
| *V. spicata* | Travnicek 1994, OL | 4x | Slovakia |  |  | 48.4782 | 20.4703 | Travnicek, 2004 |
| *V. spicata* | Travnicek 1995, OL | 4x | Slovakia |  |  | 48.4879 | 17.2659 | Travnicek, 2004 |
| *V. spicata* | Travnicek 1991, OL | 4x | Slovakia |  |  | 48.4901 | 20.4809 | Travnicek, 2004 |
| *V. spicata^2^* | Travnicek 1991, OL | 4x | Slovakia |  |  | 48.5243 | 20.0743 | Travnicek, 2004 |
| *V. spicata* | Travnicek 1994, OL | 4x | Slovakia |  |  | 48.5243 | 20.0751 | Travnicek, 2004 |
| *V. spicata* | Tichy 1994, OL | 4x | Slovakia |  |  | 48.5553 | 18.2995 | Travnicek, 2004 |
| *V. spicata* | Travnicek 1994, OL | 4x | Slovakia |  |  | 48.6100 | 18.3821 | Travnicek, 2004 |
| *V. spicata* | Travnicek 1994, OL | 4x | Slovakia |  |  | 48.6108 | 20.8780 | Travnicek, 2004 |
| *V. spicata* | Travnicek 1994, OL | 4x | Slovakia |  |  | 48.6429 | 18.4511 | Travnicek, 2004 |
| *V. spicata* | Travnicek, 1994, OL | 4x | Slovakia |  |  | 48.6888 | 18.4567 | Travnicek, 2004 |
| *V. spicata* | Hadinec 1994, OL | 4x | Slovakia |  |  | 48.6895 | 18.4472 | Travnicek, 2004 |
| *V. spicata* | Travnicek, 1994, OL | 4x | Slovakia |  |  | 48.6947 | 18.3823 | Travnicek, 2004 |
| *V. spicata* | Travnicek, 1995, OL | 4x | Slovakia |  |  | 48.7601 | 20.0611 | Travnicek, 2004 |
| *V. spicata* | Travnicek, 1995, OL | 4x | Slovakia |  |  | 48.9637 | 20.3945 | Travnicek, 2004 |
| *V. spicata* | Travnicek, 1995, OL | 4x | Slovakia |  |  | 49.0072 | 20.7234 | Travnicek, 2004 |
| *V. spicata* | Travnicek, 1995, OL | 4x | Slovakia |  |  | 49.0173 | 20.3846 | Travnicek, 2004 |
| *V. spicata* | Travnicek, 1995, OL | 4x | Slovakia |  |  | 49.1067 | 19.4400 | Travnicek, 2004 |
| *V. spicata* | Lökvist 1274, LD | 4x | Sweden |  |  | 55.3848 | 14.0513 | Lökvist and Hultgard, 1999 |
| *V. spicata* | Lökvist 1374, LD | 4x | Sweden |  |  | 55.3870 | 14.1400 | Lökvist and Hultgard, 1999 |
| *V. spicata* | Lökvist 1303, LD | 4x | Sweden |  |  | 55.5576 | 14.3039 | Lökvist and Hultgard, 1999 |
| *V. spicata* | Lökvist 1281, LD | 4x | Sweden |  |  | 55.7154 | 14.1095 | Lökvist and Hultgard, 1999 |
| *V. spicata* | Lökvist 1448, LD | 4x | Sweden |  |  | 56.1756 | 13.8191 | Lökvist and Hultgard, 1999 |
| *V. spicata* | Lökvist 2303, LD | 4x | Sweden |  |  | 56.3017 | 12.4517 | Lökvist and Hultgard, 1999 |
| *V. spicata* | Lökvist 2619, LD | 4x | Sweden |  |  | 56.4330 | 12.7322 | Lökvist and Hultgard, 1999 |
| *V. spicata* | Lökvist 2609, LD | 4x | Sweden |  |  | 56.4349 | 12.5649 | Lökvist and Hultgard, 1999 |
| *V. spicata* | Lökvist 2557, LD | 4x | Sweden |  |  | 56.5323 | 16.5164 | Lökvist and Hultgard, 1999 |
| *V. spicata* | Lökvist 2475, LD | 4x | Sweden |  |  | 56.6800 | 16.7000 | Lökvist and Hultgard, 1999 |
| *V. spicata* | Lökvist 2574, LD | 4x | Sweden |  |  | 56.7960 | 16.5830 | Lökvist and Hultgard, 1999 |
| *V. spicata* | Lökvist 2492, LD | 4x | Sweden |  |  | 56.9672 | 16.7260 | Lökvist and Hultgard, 1999 |
| *V. spicata* | Lökvist 1351, LD | 4x | Sweden |  |  | 58.3010 | 13.7811 | Lökvist and Hultgard,1999 |
| *V. spicata* | Lökvist 2259, LD | 4x | Sweden |  |  | 58.8077 | 17.6702 | Lökvist and Hultgard,1999 |
| *V. spicata* | Bardy sw01-1, WU | 4x | Sweden | **1.5369** | 7.93 | 59.8939 | 17.6214 | new result |
| *V. spicata^2^* | Höpke 307, OLD | 4x | Ukraine | **1.4367** | 10.04 | 48.7607 | 35.4615 | new result |
| *V. spicata* | Höpke 283, OLD | 4x | Ukraine | **1.3323** | 4.89 | 50.5335 | 30.5265 | new result |
