# Supplementary material for: Comparative Phylogeography of Veronica spicata and V. longifolia (Plantaginaceae) Across Europe: Integrating Hybridization and Polyploidy in Phylogeography
Source: Front Plant Sci. 2021 Feb 1;11:588354. doi: 10.3389/fpls.2020.588354 (PMC7884905; doi:10.3389/fpls.2020.588354)
Supplement: Supplementary file 8 [file Table_3.docx]

| **TABLE S3 \|** Pairwise *F*_ST_ results between species | | | | | |
| --- | --- | --- | --- | --- | --- |
| **Population1** | **Population2** | **P-value** | ***F*_ST_** | **Lower bound CI Limit** | **Upper bound CI Limit** |
| *V. spicata* | *V. longifolia* | 0 | 0.294 | 0.292 | 0.296 |
| *V. spicata* | *× media* | 0 | 0.091 | 0.087 | 0.095 |
| *V. longifolia* | *V. × media* | 0 | 0.047 | 0.044 | 0.05 |
| *V. spicata* | *V. schmidtiana* | 0 | 0.632 | 0.627 | 0.637 |
| *V. longifolia* | *V. schmidtiana* | 0 | 0.625 | 0.62 | 0.629 |
| *V. × media* | *V. schmidtiana* | 0 | 0.768 | 0.761 | 0.773 |
