# Supplementary material for: Comparative Phylogeography of Veronica spicata and V. longifolia (Plantaginaceae) Across Europe: Integrating Hybridization and Polyploidy in Phylogeography
Source: Front Plant Sci. 2021 Feb 1;11:588354. doi: 10.3389/fpls.2020.588354 (PMC7884905; doi:10.3389/fpls.2020.588354)
Supplement: Supplementary file 9 [file Table_4.docx]

Supplement XX Extension of Tab. 4. One-way Anova calculations comparing bioclim variables (Hijmans et al. 2005) of di- (2) and tetraploid (4) *Veronica longifolia* and of diploid (2) and tetraploid (4) *Veronica spicata*. The latter was tested with and without the diploid Siberian samples (2 total and 2 west, respectively). The *V. spicata* mean 4 column is valid for both comparisons. Significant results (p < 0.05) are in bold. Significant results after Bonferroni correction for multiple tests (p < 0.003) have an asterisk.

| bioclim variable^#^ | *V. longifolia* 2 / 4 | | | *V. spicata* 2 total / 4 | | | *V. spicata* 2 west / 4 | |
| --- | --- | --- | --- | --- | --- | --- | --- | --- |
|  | mean 2 (N=40) | mean 4 (N=65) | p-value | mean 2 (N= 74) | mean 4 (N=69) | p-value | mean 2 (N=33) | p-value |
| bio1 | 2.95 | 4.18 | 0.265 | 4.46 | 7.88 | **< 0.001*** | 7.65 | 0.582 |
| bio2 | 8.99 | 9.58 | **0.047** | 9.65 | 8.62 | **< 0.001*** | 8.42 | 0.431 |
| bio3 | 25.20 | 25.61 | 0.629 | 25.36 | 29.00 | **< 0.001*** | 29.10 | 0.894 |
| bio4 | 997.38 | 1096.94 | 0.136 | 1117.09 | 787.96 | **< 0.001*** | 773.50 | 0.551 |
| bio5 | 21.95 | 24.19 | **< 0.001*** | 24.75 | 23.59 | **0.009** | 22.95 | 0.277 |
| bio6 | -14.33 | -14.70 | 0.854 | -14.60 | -6.23 | **< 0.001*** | -6.22 | 0.988 |
| bio7 | 36.28 | 38.89 | 0.185 | 39.35 | 29.81 | **< 0.001*** | 29.17 | 0.383 |
| bio8 | 13.39 | 16.90 | **< 0.001*** | 16.20 | 16.32 | 0.861 | 13.68 | **0.006** |
| bio9 | -5.18 | -7.18 | 0.267 | -7.10 | 0.11 | **< 0.001*** | 0.93 | 0.292 |
| bio10 | 15.27 | 17.26 | **< 0.001*** | 17.72 | 17.40 | 0.365 | 16.92 | 0.332 |
| bio11 | -9.26 | -9.59 | 0.860 | -9.50 | -1.94 | **< 0.001*** | -1.99 | 0.918 |
| bio12 | 568.35 | 516.69 | 0.076 | 576.60 | 633.14 | 0.111 | 806.38 | **< 0.001*** |
| bio13 | 76.63 | 74.45 | 0.479 | 77.95 | 81.72 | 0.272 | 98.34 | **< 0.001*** |
| bio14 | 26.33 | 23.45 | 0.200 | 30.00 | 32.62 | 0.322 | 47.16 | **< 0.001*** |
| bio15 | 39.48 | 42.85 | 0.334 | 36.48 | 32.40 | **0.031** | 27.24 | **0.009** |
| bio16 | 208.80 | 201.48 | 0.410 | 205.54 | 226.54 | **0.043** | 270.53 | **< 0.001*** |
| bio17 | 85.88 | 77.75 | 0.258 | 98.34 | 105.32 | 0.406 | 153.31 | **< 0.001*** |
| bio18 | 190.13 | 199.62 | 0.295 | 198.46 | 219.42 | **0.035** | 254.88 | **0.003** |
| bio19 | 108.88 | 87.02 | **0.015** | 110.24 | 119.78 | 0.321 | 171.34 | **< 0.001*** |

^#^ Definition of the bioclim variabels: bio1 annual mean temperature, bio2 mean diurnal range, bio3 isothermality, bio4 temperature seasonality, bio5 max. temperature of warmest period, bio6 min. temperature of coldest period, bio7 temperature annual range, bio8 mean temperature of wettest quarter, bio9 mean temperature of driest quarter, bio10 mean temperature of warmest quarter, bio11 mean temperature of coldest quarter, bio12 annual precipitation, bio13 precipitation of wettest period, bio14 precipitation of driest period, bio15 precipitation seasonality. bio16 precipitation of wettest quarter, bio17 precipitation of driest quarter, bio18 precipitation of warmest quarter, bio19 precipitation of coldest quarter.
